# Chromium‐Doped NiBP Micro‐Sphere Electrocatalysts for Green Hydrogen Production under Industrial Operational Conditions

**DOI:** 10.1002/smtd.202401939

**Published:** 2025-01-19

**Authors:** Md Ahasan Habib, Shusen Lin, Sumiya Akter Dristy, Mehedi Hasan Joni, Rutuja Mandavkar, Jae‐Hun Jeong, Jihoon Lee

**Affiliations:** ^1^ Department of Electronic Engineering College of Electronics and Information Kwangwoon University Nowon‐gu Seoul 01897 South Korea

**Keywords:** Cr‐doping, HER and OER, high‐current, hydrogen generation, water splitting

## Abstract

Wide spread adaptation of green hydrogen can help to mitigate the serious climate issues, increasing global energy demands and the development of advanced electrocatalysts robust under industrial conditions is one of the key technological challenges. Herein, chromium‐doped nickel‐boride‐phosphide (Cr/NiBP) micro sphere (MS) electrocatalyst is demonstrated via a two‐step hydrothermal approach along with post‐annealing. The Cr/NiBP MS demonstrates low hydrogen evolution reaction and oxygen evaluation reaction over potentials of 78 and 250 mV at 100 mA cm^−2^ in 1 m KOH, out performing most of the reported catalysts. The Cr/NiBP ǁ Cr/NiBP exhibits only 1.54 V at 100 mA cm^−2^ in 1 m KOH and surpasses the benchmark of RuO_2_ (+) ǁ Pt/C (−) up to 2000 mA cm^−2^, which sets it as one of the best bifunctional electrocatalysts. Impressively, it maintains stable performance for over 240 h at 1000 mA cm^−2^ in 6 m KOH at 60°C, demonstrating rapid response, anti‐corrosion resistance, and robust structural integrity to meet the industrial operational conditions. Further, Cr/NiBP ǁ Pt/C exhibits a super‐low cell‐voltage of 2.25 V at 2000 mA cm^−2^. The small amount of Cr atoms incorporation can significantly enhance active sites and intrinsic properties, accelerating water dissociation and rapid intermediate formation.

## Graphical abstract

The bifunctional Cr/NiBP ǁ Cr/NiBP demonstrates high‐performance overall water splitting over benchmark Pt/C ǁ RuO_2_. Coupling with Pt/C, the hybrid configuration of Cr/NiBP ǁ Pt/C shows more improved performance up to 2000 mA cm^−2^ high current density.

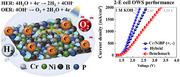

## 1 Introduction

With the threat of environmental issues associated with the excessive consumption of fossil fuels and ever‐increasing energy demands, the exploration of alternative energy resources has become an urgent priority.^[^ ^1^, ^2^ ^]^ Green hydrogen (H_2_) can be considered as one of the most promising sustainable energy alternatives to traditional fossil fuels due to its environmental friendliness, high energy density, and storage advantages.^[^ ^3^, ^4^ ^]^ Water electrolysis can be a technology for producing high‐purity green H_2_,^[^ ^5^ ^]^ and the overall water splitting (OWS) consists of two half‐reactions of hydrogen evolution reaction (HER: 4H_2_O + 4e− → 2H_2_ + 4OH^−^) at the cathode and oxygen evolution reaction (OER: 4OH^−^ → O_2_ + 2H_2_O + 4e−) at the anode.^[^ ^6^, ^7^ ^]^ At present, noble metal‐based Pt/C and RuO_2_ are the benchmark electrocatalysts HER and OER, respectively, however, their high cost and rare earth elemental nature hinder the widespread commercial applications.^[^ ^8^, ^9^ ^]^ Industrial electrolysis typically involves high KOH concentrations, elevated temperatures, and high current densities (HCD) to enhance the catalytic efficiency.^[^ ^10^, ^11^ ^]^ Low HER/OER overpotentials and high durability under HCD are crucial for advanced electrocatalysts.^[^ ^10^, ^11^ ^]^ Especially, superior OER capability is crucial for advanced bi‐functional electrocatalysts, as the four‐electron transfer OER process requires high energy input. The development of advanced electrocatalysts with high‐performance and stable operation under HCD and high alkaline conditions as well as cost‐effectiveness is essential now.

Recently, transition metals (TMs) based compounds such as TM‐sulfides, ‐borides, ‐phosphides, ‐oxides, ‐carbides, etc. have been actively researched as promising electrocatalyst materials due to their superior intrinsic water splitting capabilities and economic advantages.^[^ ^12^ ^]^ Unfilled d‐orbitals of TMs can induce a strong interaction with non‐metallic elements and TM‐based compounds can effectively accelerate the HER/OER reactions by precisely tuning adsorption/desorption characteristics for the reaction intermediates.^[^ ^12^, ^13^ ^]^ Among various TMs, nickel (Ni) can be a superior HCD OER element along with its high electrical conductivity, valance states, and intrinsic properties.^[^ ^14^, ^15^ ^]^ For example, the Ni/NiO porous electrode exhibited efficient OER performance due to its high reaction kinetics and large electrochemical surface area (ECSA) for faster water dissociation.^[^ ^16^ ^]^ At the same time, the selection of the non‐metallic element is equally important for optimizing the electrocatalytic activities based on the synergistic effect of each element. The phosphorus (P) with high electron‐negativity can effectively trap the protons (H ^+^) and weaken H─O─H bonds toward faster H_2_O dissociation.^[^ ^17^ ^]^ For instance, the CoP‐Ni_2_P‐Fe_2_P electrocatalyst demonstrated superior HER due to the effective elemental interaction, high conductivity, and abundant active species.^[^ ^18^ ^]^ Meanwhile, boron (B) as another non‐metallic element can efficiently promote HCD OER through strong electron transfer and orbital hybridization nature.^[^ ^19^, ^20^ ^]^ TM─B bonds can achieve desirable structural configurations and B‐oxide species can offer strong anti‐corrosion capability.^[^ ^21^, ^22^ ^]^ For example, the B‐FeNi exhibited improved OER and surface robustness by obtaining high intrinsic activity, favorable intermediate formation, and electrochemical stability.^[^ ^23^ ^]^ Additionally, metallic atom doping (MAD) into the bulk phase electrocatalysts can further improve the intrinsic properties, electrocatalytic performance, and HCD stability.^[^ ^24^ ^]^ MAD can enlarge ECSA, enhance catalytic efficiency, and alter charge transfer characteristics by introducing new active sites and induing strong electrochemical interactions.^[^ ^25^, ^26^ ^]^ At the same time, the forced incorporation of metallic atoms may introduce crystalline damages, resulting in polycrystalline structures, which can induce better electrocatalytic performances as compared with the single crystal counterparts due to the stable electrochemical nature of polycrystalline phase materials with the increased active species and ECSA.^[^ ^26^ ^]^ Only few percentages of metallic atoms inclusion can be an effective strategy to reduce the energy barriers of HER/OER.^[^ ^27^ ^]^ Among various active metallic dopants, chromium (Cr) can effectively tune HER/OER capabilities due to its high charge transfer kinetics and optimum adsorption/desorption energies of water electrolysis intermediates of H*, OH*, O*, and OOH*, enhancing both HER and OER.^[^ ^28^, ^29^, ^30^ ^]^ To this end, optimizing Cr‐doping in TM‐boride‐phosphide would be a practical approach for developing advanced electrocatalysts with high OWS capabilities under HCD and high alkaline conditions, which has never been explored yet. The Cr‐doped TM‐BP may exhibit enhanced electrochemical capabilities and structural flexibility with abundant active sites and high intrinsic activity.

**Figure 1:**
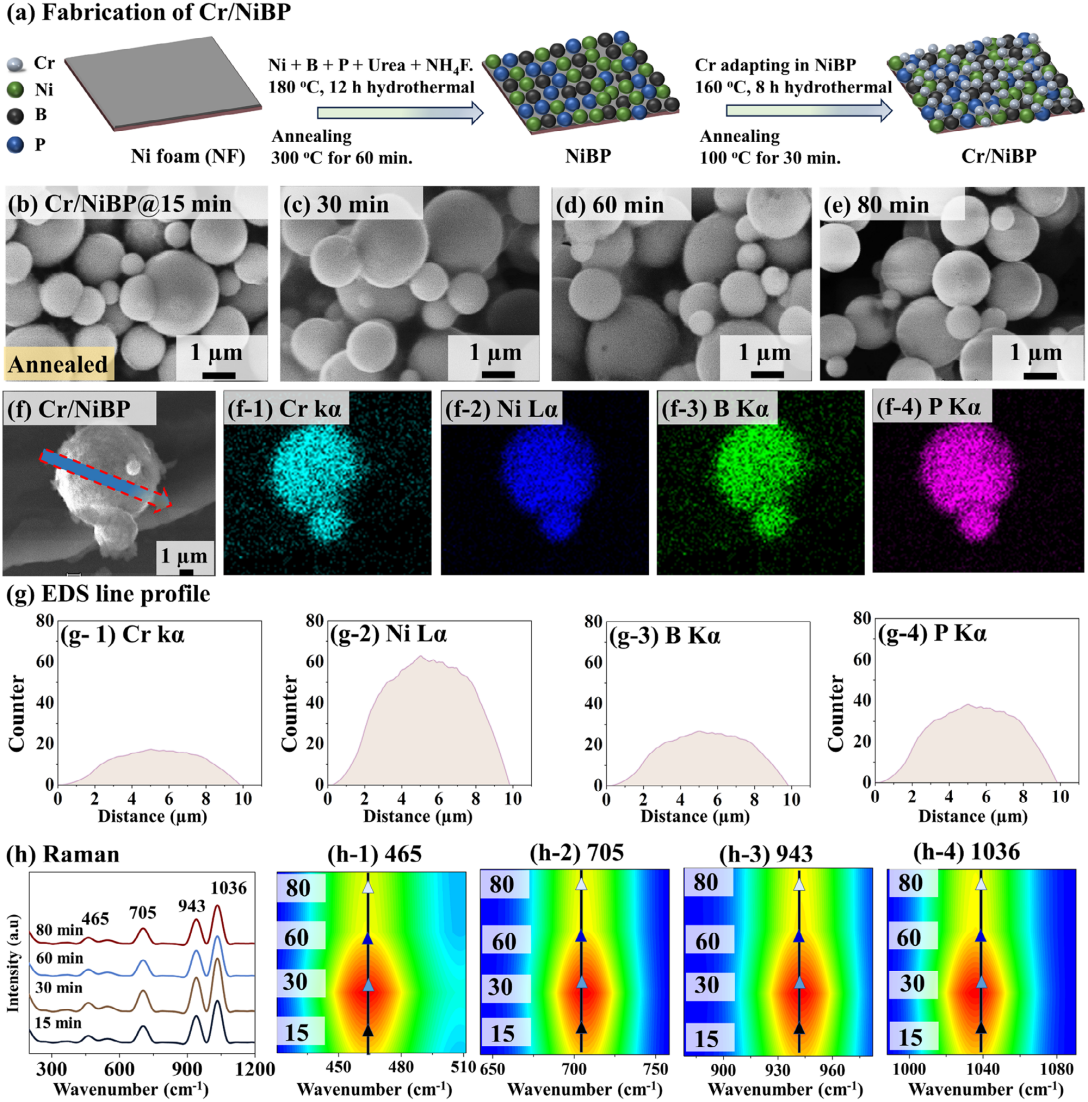
Cr/NiBP micro sphere (MS) electrodes with post‐annealing duration. a) Schematic illustration of the fabrication process. b–e) SEM images. f–f‐4) Top‐view SEM and corresponding elemental phase map. g) EDS line profile. h) Raman spectra. h‐1–h‐4) Contour plots.

In this work, a Cr‐doped NiBP micro sphere (MS) electrocatalyst, namely Cr/NiBP MS, is successfully synthesized using a combined two‐step hydrothermal method toward the accelerated HER/OER for the first time. Initially, the NiBP micro‐spheres are fabricated on a bare nickel foam (NF) as illustrated in **Figure** **1**a ^[^ ^31^ ^]^ and then, various Cr‐doping parameters including concentration, hydrothermal temperature, and duration are symmetrically optimized through the second step hydrothermal reaction. In addition, a double‐step post‐annealing approach is adapted before/after the Cr doping to improve the overall crystallinity. The Cr/NiBP MS exhibits highly comparable HER performance to the Pt/C and significantly higher electrocatalytic OER activity than the RuO_2_, surpassing most of the reported state‐of‐the‐art works. The Cr/NiBP MS demonstrates superior high‐current bifunctional characteristics, achieving a low cell voltage of 2.63 V at HCD of 2000 mA cm^−2^ in 1 m KOH as compared to the benchmark value of 3.33 V. Additionally, Cr/NiBP MS exhibits HER/OER bi‐functional properties, electrocatalytic robustness, and exceptional hybrid characteristics under harsh industrial conditions, making it a promising candidate for large‐scale ultra‐pure green hydrogen production. A small amount of Cr‐doping in the NiBP matrix can induce significantly improved electrocatalytic properties, water dissociation capability, high conductivity, and enlarged ECSA for WS application.

## 2 Synthesis and Electrode Characterizations

The Cr/NiBP micro spherical (MS) electrocatalyst is prepared by a two‐step hydrothermal method as illustrated in Figure 1a and the flow chart of the synthesis process can be found in Figure S1 (Supporting Information). In this work, NiBP is used as a cost‐effective doping template due to promising electrochemical properties, synergistic effects, and structural integrity. For example, Ni‐based material can demonstrate superior OER characteristics due to good water dissociation ability, high reaction adsorbates, and multi‐pole valance state.^[^ ^32^ ^]^

**Figure 2:**
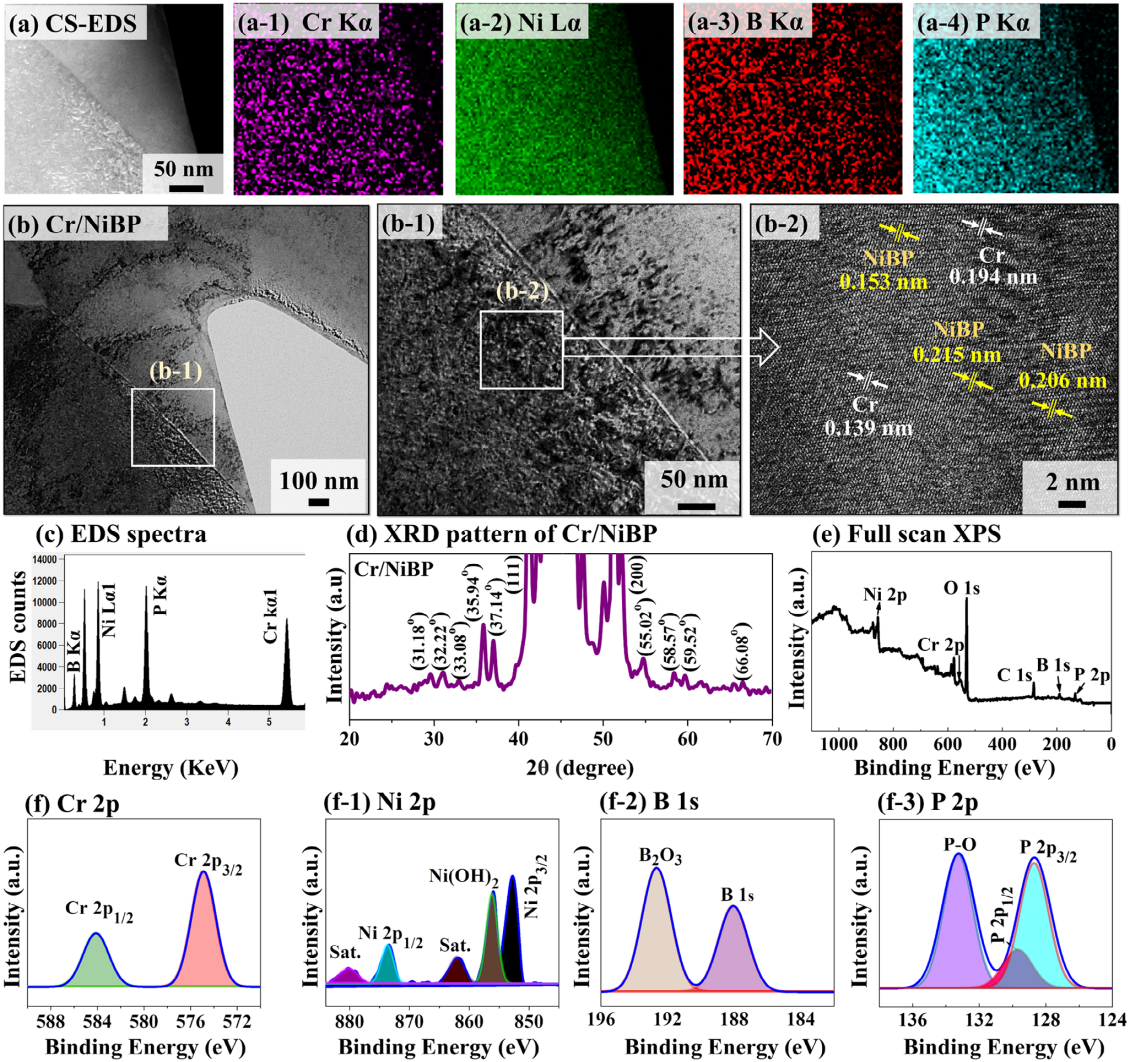
Structural and compositional characterization of the best Cr/NiBP MS electrode (30 min annealed). a–a‐4) EDS cross‐sectional maps. b–b‐2) High‐resolution TEM. c) EDS Spectra. d) XRD pattern. e) Full scan XPS and high‐resolution XPS spectra of f) Cr 2p, f‐1) Ni 2p, f‐2) B 1s and f‐3) P 2p.

**Figure 3:**
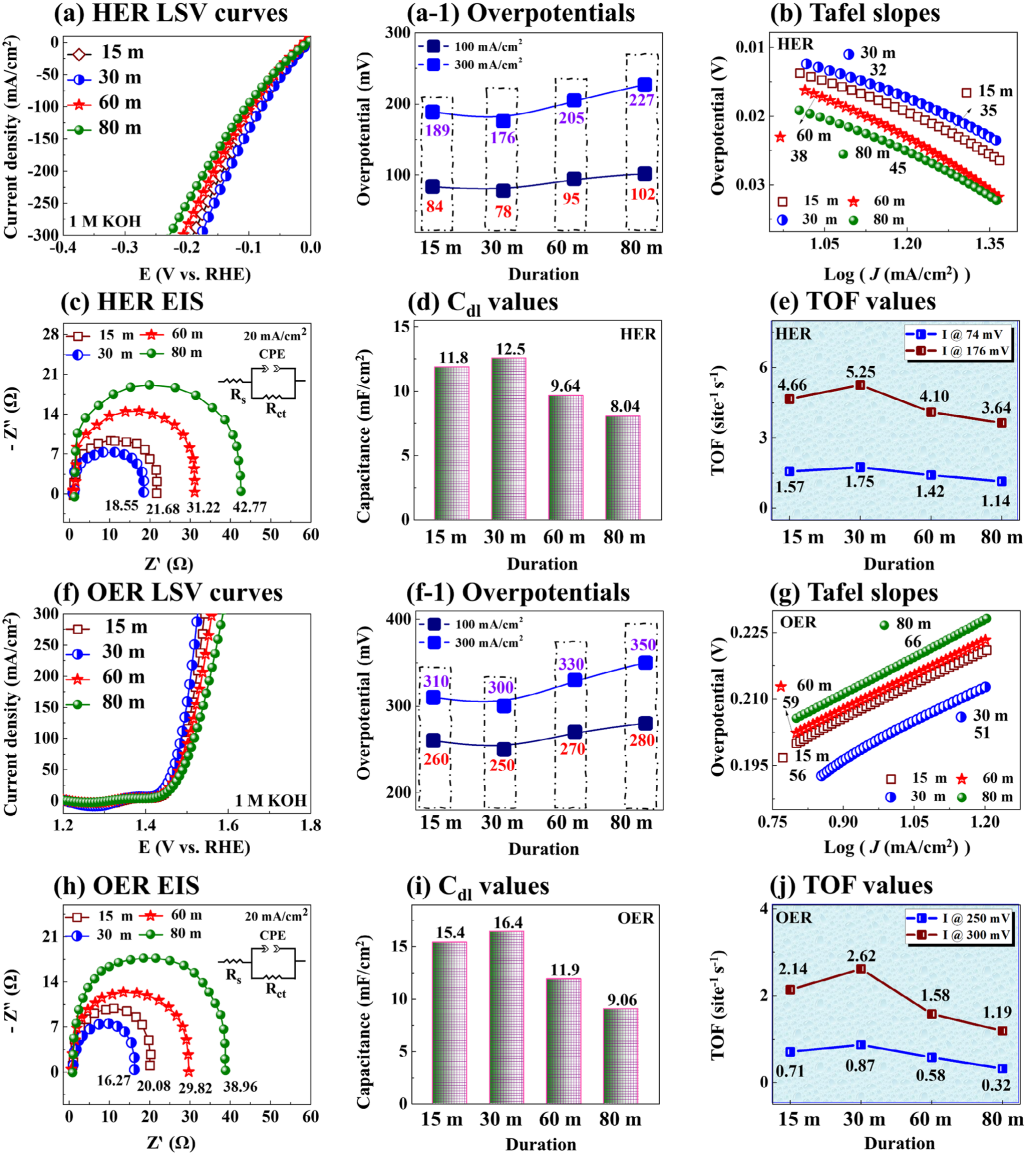
Electrochemical HER and OER properties of Cr/NiBP MS electrocatalysts with post‐annealing duration variation. a,f) HER and OER LSV. a‐1,f‐1) Overpotential comparisons. b,g) Tafel plots. c,h) EIS Nyquist plots with corresponding equivalent circuits. d,i) Double layer capacitance (*C_dl_)* values. e,j) Turnover frequency (TOF) values.

**Figure 4:**
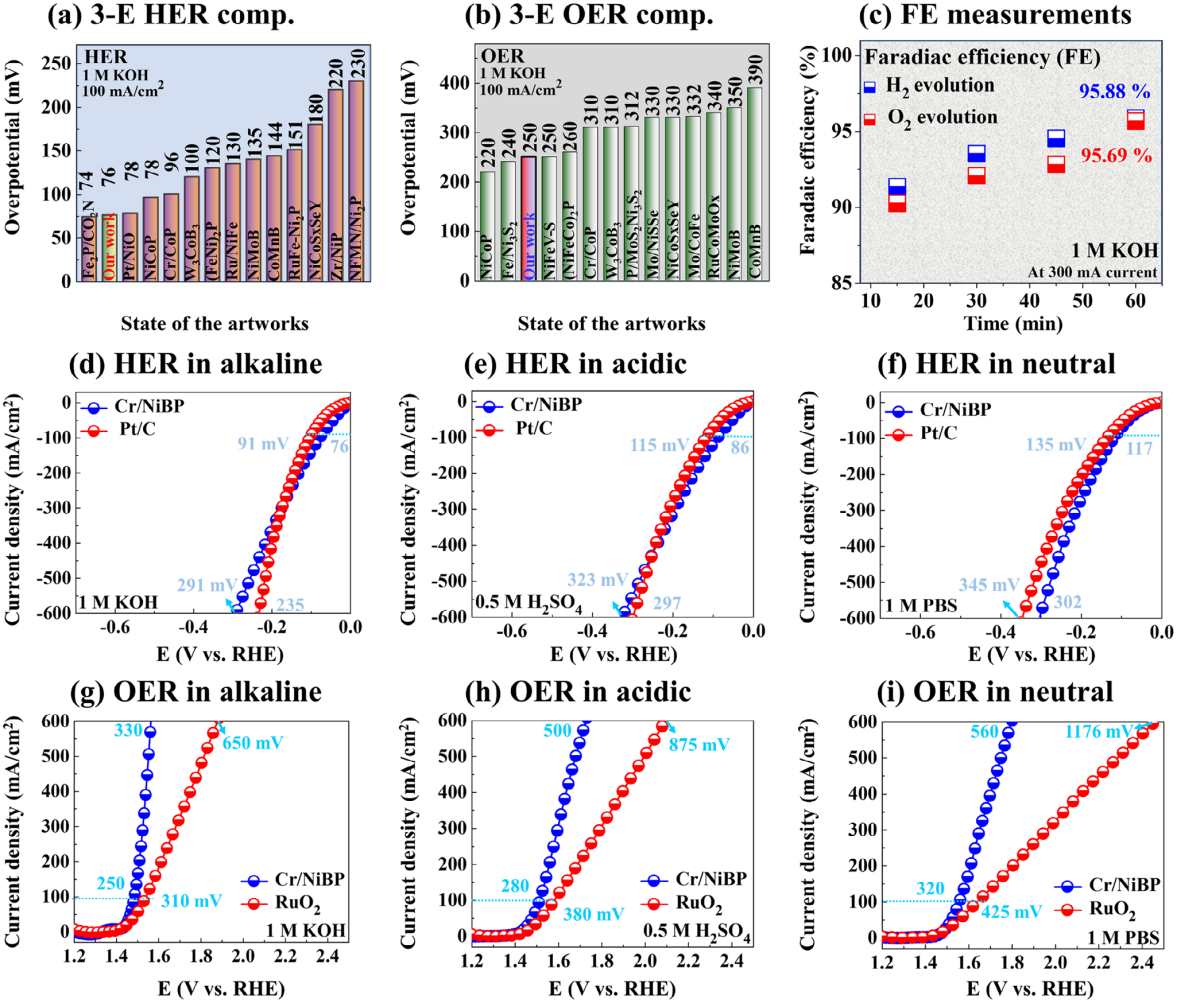
Electrocatalytic HER and OER activity comparisons with reported works, Faradaic efficiency and HER/OER performance in different pH. a,b) Comparisons of HER and OER performance at 100 mA cm^−2^ in 1 m KOH (Related to Tables S1 and S2, Supporting Information). c) Faradaic efficiency measurement. d–g) HER and OER performance of Cr/NiBP as compared to the benchmarks in 1 m KOH, 0.5 m H_2_SO_4_ and 1 m PBS.

The unique electronic properties of B can facilitate strong bonding with deposited atoms while P‐containing electrodes enhance proton adsorption through rapid charge carrier mobility.^[^ ^33^ ^]^ Briefly, NiBP spheres were synthesized on Ni foam (NF) via the first step of hydrothermal reaction. Subsequently, Cr doping was introduced onto the NiBP template through a second‐step hydrothermal reaction toward advanced catalytic efficiency. Before/after post‐annealing was adopted on optimized Cr/NiBP MS to improve crystallinity and conductivity. The detailed characterizations of bare NF, LSV scan rate optimization, electrochemical impedance spectroscopy (EIS) voltage control, benchmarks, and NiBP template were provided in Figures S2–S11 (Supporting Information). The Cr‐doping optimization process was carried out to maximize the HER/OER capability with additional active sites, enlarged ECSA, and intrinsic activity.^[^ ^29^, ^34^ ^]^ The appropriate Cr could play an important role in obtaining advanced activity by optimizing the adsorption/desorption capacity.^[^ ^35^ ^]^ Various Cr‐doping controlling parameters, including concentration, reaction temperature, and duration were systemically evaluated in Figures S12–S21 (Supporting Information). In terms of optimization, the 0.4 mm Cr‐doped NiBP at 160 °C for 8 h demonstrated the best HER/OER. A detailed synthesized process can be found in Text S1.1 (Supporting Information). The annealing duration was controlled between 15 and 80 min at 100 °C on the best Cr/NiBP MS in Figure 1b–e. The surface of Cr/NiBP electrodes with post‐annealing duration variation did not show any specific morphological differences in scanning electron microscopy (SEM) analysis. The vertically aligned spherical cluster morphology of Cr/NiBP could enable rapid electrolyte infiltration into the internal cavity and efficient gas product release, leading to enhanced HER/OER performance. The post‐annealing temperature and duration control‐related data can be found in Figures S22–S29 (Supporting Information). The top‐view energy‐dispersive x‐ray spectroscopy (EDS) mapping demonstrated Cr Kα, Ni Lα, B Kα, and P Kα phases as shown in Figure 1f–f‐4, confirming the successful Cr inclusion. The line scan profile exhibited a uniform distribution of Cr, Ni, B, and P elements throughout the micro sphere as shown in Figure 1g. Additionally, EDS elemental analysis (Figure S25, Supporting Information) confirmed that a relatively low content of Cr (atomic ratio 5.70%) was used here. During forced incorporation of Cr atoms under high pressure and temperature, the Cr/NiBP MS may contain various defects and atomic dislocation in the crystal lattice.^[^ ^12^ ^]^ Optimum annealing can improve the overall crystallinity by reducing various dislocations and defects with atomic diffusion.^[^ ^12^ ^]^ To investigate overall crystallinity, the Raman spectra were performed in Figure 1h. The Cr/NiBP samples demonstrated characteristic peaks at 465, 705, 943, and 1036 cm^−1^. In counterplots, the 30 min annealed electrode exhibited the highest intensity and crystal quality in Figure 1h‐1–h‐4, suggesting the best crystallinity in this set. A more detailed Raman analysis can be found in Text S1.7 (Supporting Information).

**Figure** **2** shows the structural analysis of the Cr/NiBP MS with cross‐sectional (CS) mapping, transmission electron microscopic (TEM), X‐ray diffraction (XRD) pattern, and X‐ray photoelectron spectroscopy (XPS) characterizations. After focused ion beam (FIB) preparation, the CS mapping was conducted on deep microns inside as seen in Figure 2a–a‐4. The presence of Cr, Ni, B, and P atoms is confirmed throughout the nano‐structure. The TEM analysis was performed to observe the internal crystal structure of Cr/NiBP as shown in Figure 2b–b‐2. The high‐resolution TEM image revealed randomly oriented various lattice fringes as presented in Figure 2b‐2. The TEM analysis demonstrated that the initially grown NiBP exhibited a polycrystal nature and then, the Cr atoms were incorporated as nano‐crystal domains. Here, the lattice fringes of 0.153, 0.206, and 0.215 nm can be assigned to NiBP phases. Also, the interplanar distances of 0.194 and 0.139 nm can correspond to Cr phases. This indicated that the polycrystal matrix is mainly composed of many small crystals. The Cr atoms were successfully distributed all over the NiBP matrix under hydrothermal high‐pressure and temperature reactions. Additional TEM analysis of Cr/NiBP from different areas demonstrated consistency with interplanar distances and polycrystal features as shown in Figure S37 (Supporting Information). The specific crystallographic planes could not be identified due to the polycrystalline nature of the Cr/NiBP material with numerous tiny crystals in various orientations.^[^ ^32^ ^]^ Overall, the polycrystal surface of the Cr/NiBP MS likely provides a larger number of active sites and accelerates charge transfer kinetics, which can improve the overall material synergy.^[^ ^36^ ^]^ The EDS spectra of the Cr/NiBP are presented in Figure 2c. Further, the XRD pattern of Cr/NiBP was performed in Figure 2d. The intense peaks observed at ≈43° and 52° can be assigned to (111) and (200) crystallographic planes of NF.^[^ ^37^ ^]^ Further, the XRD pattern showed characteristics peaks at 31.18°, 32.22°, 33.08°, 35.94°, 37.14°, 55.02°, 58.57°, 59.52°, and 66.8°. The formation of many small crystals also indicated the successful preparation of the Cr/NiBP polycrystal structure, which agrees well with TEM analysis. The XRD comparisons between bare NiBP and Cr/NiBP and related PDF cards of close materials system were shown in Figures S31 and S39 (Supporting Information). Notably, after doping, no additional peaks associated with Cr‐related phase formation were observed, indicating successful nanocrystal incorporation and low doping content. This was confirmed by EDS elemental and TEM analysis. The XRD pattern of Cr/NiBP and NiBP was not found in the literature or database due to likely polycrystalline phases. A more XRD‐related analysis on Cr/NiBP can be found in Text S1.8 (Supporting Information). In addition, the polycrystal surface can outperform single crystals with faster reaction kinetics owing to their available active sites, enlarged surface area, and structural stability.^[^ ^38^ ^]^

The XPS analysis is performed on Cr/NiBP MS to investigate the surface chemistry and chemical composition as shown in Figure 2e–f‐3. The full scan survey spectra further demonstrated peaks of Cr, Ni, B, and P elements in Figure 2e. Additionally, the observed C 1s and O 1s peaks were derived from binding energy (BE) calibration and exposure to air. The elemental BEs of nickel (Ni 2p_1/2_), boron (B 1s), phosphorous (P 2p_1/2_), and chromium (Cr 2p_3/2_) were observed at 873.1, 188.1, 130.4, and 574.9 eV as shown in Figure 2f–f‐3. The pristine elemental positions of Ni 2p_1/2_, B 1s, P 2p_1/2_, and Cr 2p_3/2_ can be found at 869.97, 187.3, 130.74, and 574.4 eV in the XPS handbook.^[^ ^39^ ^]^ In the Ni 2p spectrum, the Ni 2p_1/2_ peak was shifted positively by 3.13 eV from pristine as shown in Figure 2f‐1, indicating the donation of electrons. Generally, the specific BEs shifting can reveal bond/compound formation by electrotonic interaction. The Ni 2p_3/2_ peak also shifted positively from 852.7 to 852.8 eV (by 0.1 eV) for supplying electrons. The Ni satellite peaks were observed at 879.8 and 861.8 eV.^[^ ^40^ ^]^ The deconvoluted peak at 856.2 eV is associated with Ni(OH)_2_ species due to the adsorption of hydroxide ions, which can subsequently transform into the higher valance state of Ni^+3^ (NiOOH).^[^ ^41^ ^]^ Further, the B 1s peak was shifted positively by 0.8 eV toward higher BE (from 187.3 to 188.1 eV) in Figure 2f‐2, indicating the supply of electrons. The trivalent boron oxide species (B_2_O_3_) was observed at 192.6 eV.^[^ ^42^ ^]^ In the P 2p spectrum, the P 2p_3/2_ and P 2p_1/2_ peaks were located at 128.78 and 129.60 eV in Figure 2f‐3. The elemental positions of P 2p_3/2_ and P 2p_1/2_ can be found at 129.90 and 130.74 eV. This indicated that the P peaks were shifted negatively by 1.12 and 1.14 eV for receiving electrons. Further, the oxidized P─O species were located at 133.3 eV.^[^ ^43^ ^]^ These results suggest a strong electronic interaction and valance electron transfer between Ni, B, and P. As compared to BE shifts, the Ni and B might have donated electrons to the P atom, facilitating orbital hybridization and NiBP formation. The TEM and XRD analysis also demonstrated that Cr atoms were incorporated separately in the NiBP matrix as a degree of nanocluster. Last, the Cr 2p_3/2_ and Cr 2p_1/2_ peaks were located at 574.9 and 584 eV in the Cr 2p spectrum as illustrated in Figure 2f. The elemental positions of Cr 2p_3/2_ and Cr 2p_1/2_ can be found at 574.4 and 583.6 eV. This revealed that both Cr species were shifted positively by 0.5 and 0.4 eV, suggesting the increased local electron density around the NiBP matrix. The BE of both Cr species is related to the trivalent state, suggesting that metallic Cr can exhibit in the form of Cr^3+^, which is consistent with previous literature.^[^ ^28^ ^]^ The electron‐deficient Cr atoms in the NiBP matrix can work as an oxyphilic site to promote catalytic activity and faster H_2_O dissociation.^[^ ^28^ ^]^ The minor BE shift of Cr species can be attributed to the lattice strain and nano‐cluster formation with free carrier redistribution.^[^ ^32^ ^]^ The lattice strain can enhance intrinsic activity, active site accessibility, and favorable adsorption/dissociation capabilities.^[^ ^44^ ^]^ Overall, the XPS analysis further exhibited that Cr species were successfully incorporated into the NiBP structure. A more detailed XPS‐related discussion can be found in Text S1.9 (Supporting Information).

## 3 Results and Discussion

### 3.1 Electrocatalytic Properties and Mechanism Investigation

**Figure** **3** shows the HER/OER properties of Cr/NiBP electrocatalysts with different post‐annealing durations in 1 m KOH using a three‐electrode system. The linear sweep voltammetry (LSV) curves demonstrated variable performance along with variable annealing duration as illustrated in Figure 3a,f. The 30 min annealed Cr/NiBP demonstrated the lowest HER/OER overpotentials of 176 and 300 mV at 300 mA cm^−2^ as summarized in Figure 3a‐1–f‐1. Here, short annealing durations (up to 30 min) enhanced HER/OER activity, while prolonged annealing increased overpotential due to excessive diffusion energy, potentially disrupting the nanostructure.^[^ ^45^ ^]^ The higher activity of the 30 min annealed Cr/NiBP can be attributed to improved crystallization, which is particularly important for minimizing charge transfer resistance. The NiBP showed 327 and 450 mV overpotentials at 300 mA cm^−2^ for HER and OER (Figure S9a,b, Supporting Information), which are largely reduced by 151 and 150 mV at 300 mA cm^−2^. These results demonstrated significant improvement and successful Cr incorporation. The HER mechanism can be described as H_2_O + e^− ^+ M → M─H*_ads_ + OH^−^ (Volmer step)_,_ M─H*_ads_ + H_2_O + e− → H_2 _+ M + OH^−^ (Heyrovsky step).^[^ ^46^ ^]^ The high coverage of metal‐hydride (M─H) species can be combined as M─H*_ads_ + M─H*_ads_ → H_2_ + 2M (Tafel step), where the H*_ads_ and M represent hydrogen adsorbed and metallic active sites, respectively.^[^ ^46^ ^]^ Further, the OER mechanism can be summarized as 1) M + OH^−^ → M─OH* + e^−^, 2) M─OH* + OH^−^→ M─O* + H_2_O + e^−^, 3) 2M─O* → 2M + O_2_ or M─O* + OH^−^ → M─OOH* + e^−^, 4) M─OOH* + OH^−^ → O_2 _+ H_2_O + M+ e^−^.^[^ ^46^ ^]^ The formation of H*_ads_, *OH, M─O* and OOH* intermediates is important and the bindings of the intermediates should not be too strong or too loose to facilitate rapid reaction kinetics.^[^ ^46^, ^47^, ^48^ ^]^ The abundant active species can accelerate the formation of faster reaction intermediates through enhanced intrinsic activity.^[^ ^46^, ^47^, ^48^ ^]^ The higher catalytic properties of Cr/NiBP can significantly accelerate water dissociation and overall reaction kinetics.^[^ ^49^ ^]^ The Cr nanoclusters can effectively lower the kinetic energy barrier, enabling faster adsorption/dissociation capabilities.^[^ ^47^ ^]^ To get deeper inside on reaction kinetics, the Tafel slopes were obtained from the linear region of the LSV curve using the equation, *η* =* a* + *b* log |j|, where the b and j represent the Tafel slope and current density as shown in Figure 3b,g.^[^ ^45^ ^]^ The 30 min Cr/NiBP exhibited the lowest HER/OER Tafel slope values of 32 and 51 mV dec^−1^, indicating the fastest reaction kinetics. This indicates that the rate‐determining step (RDS) of HER is accelerated by the Volmer–Heyrovsky.^[^ ^50^, ^51^ ^]^ The Cr incorporation into NiBP lattice is expected to accelerate the H_2_O dissociation, improving HER kinetics.^[^ ^52^ ^]^ On the other hand, efficient OER kinetics hinge on the facile cleavage of M─OH* bonds. The Cr nanoclusters can strengthen the interaction with OH─ and O intermediates, facilitating the RDS of OER, specifically the O* to OOH* conversion.^[^ ^52^ ^]^ Also, minor oxidation was observed in the OER turnover region in Figure 3f, which can be attributed to the transformation of metallic species (M^+2^ to M^+3^) as M─OH* + OH^−^→ M─OOH* + H_2_O + e− redox conversion.^[^ ^53^ ^]^ The M─OOH species can serve as reactive sites, enhance OH^−^ adsorption, and lower the kinetic barrier.^[^ ^53^, ^54^ ^]^ The newly formed M─OOH at the lower oxidation region can promote OER and act as a protection layer to mitigate corrosion.^[^ ^53^, ^54^ ^]^ To get further insights into the OER mechanism, the OER CV profile was performed in Figure S36 (Supporting Information) and a related discussion can be found in Text S1.10 (Supporting Information). The electronic interaction of Cr offers speedy diffusion of both HER/OER intermediates for advanced catalytic activity.^[^ ^49^, ^52^ ^]^

Further, the EIS was employed to analyze the charge transfer resistance with the corresponding equivalent circuit diagram as presented in Figure 3c,h. The 30 min Cr/NiBP MS demonstrated the lowest resistance (*R*_ct_) values of 18.55 and 16.27 Ω for HER/OER, indicating the highest electrical conductivity and efficient ion mobility. Recent studies suggest that Cr dopant can significantly improve electron transfer efficiency due to the presence of a high spin state (according to Hund's rule spin quantum number *S* = 3/2), thereby enhancing intrinsic conductivity.^[^ ^55^ ^]^ To investigate the electrochemical active surface area (ECSA) of the electrodes, the double‐layer capacitance (*C*_dl_) values were calculated using cyclic voltammetry (CV) curves with different scan rates as shown in Figure 3d,i. The 30 min sample exhibited the highest *C*_dl_ values of 12.55 and 16.45 mF cm^−2^ for HER/OER, suggesting the largest ECSA and better electrolyte permeation. The related CV curves (*C*_dl_ and ECSA) can be found in Figures S26–S28 (Supporting Information). In addition, the 30 min Cr/NiBP also demonstrated the largest HER/OER ECSA values of 78.12 and 102.50 cm^2^ in Figure S29a,b (Supporting Information), exhibiting numerous active sites for effective interaction.^[^ ^37^, ^56^ ^]^ The high ECSA of Cr/NiBP can facilitate water dissociation and bubble release during the electrocatalytic process, thereby enhancing catalytic performance and stability.^[^ ^55^ ^]^ The promising properties of Cr/NiBP can be attributed to effective Cr nanocrystal incorporation, active sites, and balanced adsorption/desorption characteristics, collectively improving the water‐splitting efficiency.^[^ ^52^, ^55^ ^]^ The Cr doping effect on NiBP was systematically investigated using Raman spectra, XRD, EIS, *C*_dl_, ECSA, LSV, TOF, and ECSA‐normalized LSV in Figures S30–S35 (Supporting Information). The Cr/NiBP demonstrated improved crystal quality with post‐annealing in Raman and XRD analysis. After Cr doping, the enlarged ECSA of Cr/NiBP demonstrated more availability of electroactive sites. Further, the Cr/NiBP exhibited higher TOF values than bare NiBP, suggesting faster H_2_/O_2_ generation kinetics with more efficient reactive sites. The normalized HER/OER LSV activity of Cr/NiBP exhibited still much better performance than bare NiBP, indicating enhanced intrinsic activity. These analyses clearly demonstrated that the optimized conductivity, electrochemical surface area, and performance of Cr/NiBP were largely improved over bare NiBP, further verifying the successful incorporation of Cr atoms. A more detailed discussion about the Cr‐doping effect can be found in Text S1.13 (Supporting Information).

To evaluate the intrinsic activity and H_2_/O_2_ generation rate, the turnover frequency (TOF) was calculated as seen in Figure 3e,j. The 30 min Cr/NiBP MS showed the highest TOF values of 5.25 and 2.62 site^−1 ^s^−1^ for HER/OER. The high TOF values indicated exceptional intrinsic properties due to more reactive sites and large ECSA.^[^ ^57^, ^58^ ^]^ The measured loading amount of Cr/NiBP MS was 0.20 mg cm^−2^. The detailed calculation of active sites and TOF‐related can be found in Text S1.11 (Supporting Information). Finally, the HER/OER performance was compared with the state‐of‐the‐art reported works in 1 m KOH as seen in **Figure** **4**a,b with Tables S1,S2 (Supporting Information). The Cr/NiBP demonstrated highly comparable performance and ranked the second/third best HER/OER electrocatalyst at 100 mA cm^−2^, standing as one of the promising candidates for advanced electrolysis. Further, the faradaic efficiency (FE) measurement of Cr/NiBP was performed using the gas‐displacement method in 1 m KOH in Figure 4c. The Cr/NiBP exhibited 95.88 and 95.69% efficiency for HER/OER. The digital photography of the water‐gas displacement method is provided in Figure S45 (Supporting Information). The experimentally produced H_2_/O_2_ ratio (remained 2:1 at different duration points) closely matched the theoretically estimated values in Figure S46 (Supporting Information), indicating an efficient conversion rate without obvious side reactions for practical applications. More details about the FE calculation can be found in Text S1.12 (Supporting Information). Overall, the 30‐min annealed Cr/NiBP demonstrated the best electrocatalytic properties in this set. The efficient HER/OER properties of Cr/NiBP can be attributed to microstructure morphology, abundant accessible active sites, and intrinsic activity.^[^ ^34^ ^]^ The improved conductivity results from the Cr atoms interaction and effective post‐annealing toward higher crystallinity.^[^ ^45^, ^55^ ^]^ The Cr‐doping in the NiBP lattice effectively reduced charge transfer resistance and optimized the water dissociation capacity by lowering the energy barrier, leading to a faster HER/OER process.^[^ ^30^, ^59^ ^]^ The summarized HER/OER properties of Cr/NiBP are provided in TableS3 (Supporting Information).

### 3.2 Different pH HER/OER Activity Test and Stability

The HER/OER performances of Cr/NiBP MS were evaluated in various pH conditions: alkaline (1 m KOH, pH ≈ 14), acidic (0.5 m H_2_SO_4_, pH ≈ 0), and neutral (1 m PBS, pH ≈ 7.4) as seen in Figure 4d–i. The performances were compared to the HER/OER benchmark of Pt/C and RuO_2_, respectively. A detailed benchmark fabrication process and characterizations can be found in Text S1.5 (Supporting Information). The Cr/NiBP showed 291, 323, and 302 mV overpotentials at 600 mA cm^−2^ respectively in 1 m KOH, 0.5 m H_2_SO_4_, and 1 m PBS as compared to the Pt/C values of 235, 297, and 345 mV in Figure 4d–f. The HER activity of Cr/NiBP is highly competitive with that of the benchmark Pt/C in alkaline and acidic media, demonstrating superior performance in neutral water. On the other hand, the Cr/NiBP exhibited significantly higher OER than RuO_2_ in all pH solutions as seen in Figure 4g–i. Interestingly, the Cr/NiBP showed super‐low overpotentials of 330, 500, and 560 mV at 600 mA cm^−2^ respectively in 1 m KOH, 0.5 m H_2_SO_4_, and 1 m PBS as compared to the RuO_2_ values of 650, 875, and 1176 mV. Overall, the HER/OER activity trends can be summarized as alkaline > acidic > neutral. Further, the Cr/NiBP exhibited the highest HER/OER TOF values of 10.51 and 5.25 site^−1 ^s^−1^ at 600 mA cm^−2^ in 1 m KOH among all pH solutions in Figure S40 (Supporting Information). The performance is comparatively superior in 1 m KOH due to the higher ion mobility and less corrosive nature, which can facilitate rapid charge transport and accelerate water dissociation.^[^ ^60^ ^]^ Also, the industry generally uses alkaline water for large‐scale H_2_ production.^[^ ^60^ ^]^ The high protons (H^+^) concentration from acidic water can affect surface morphology and active species of TM‐based electrocatalysts.^[^ ^45^, ^60^ ^]^ However, the Cr/NiBP exhibited promising activity in 0.5 m H_2_SO_4_ due to electrochemical properties and anti‐corrosion robustness.^[^ ^45^, ^60^ ^]^ Additionally, the water oxidation process is comparatively slower in neutral media due to the charge transfer limitation, high kinetic barrier, and heat generation.^[^ ^45^, ^60^ ^]^ The pH of natural seawater (≈7.4) is nearly similar to 1 m PBS. Furthermore, the electrodes exhibiting superior HER/OER in neutral media are relatively rare. Neutral water electrolysis is very crucial to obtain carbon neutrality and H_2_ generation. Thus, the efficient activity of Cr/NiBP can be attributed to the electron‐enriched surface, exposed active sites, and adaptability.^[^ ^45^, ^60^ ^]^ Apart from superior HER/OER, catalytic stability is a crucial parameter in assessing the suitability of materials for practical application. During operation, a large number of gas bubbles can accumulate on the electrocatalyst surface, hindering spontaneous reactions. The HER/OER chronoamperometry (CA) operation of Cr/NiBP exhibited stable currents and rapid response at various applied voltages in 1 m KOH as shown in Figure S41 (Supporting Information), confirming the release of efficient gas bubbles. Additionally, the HER/OER LSV curves were measured after 2000 cycles CV in 1 m KOH over 20 h in Figure S42 (Supporting Information), where the Cr/NiBP exhibited nearly the same performance with no substantial change. Finally, the Cr/NiBP demonstrated long‐term HER/OER durability at 600 mA cm^−2^ in 1 m KOH for 200 h (over 8 days) of continuous H_2_/O_2_ production as shown in Figure S43 (Supporting Information). The HER and OER activity comparison between Cr/NiBP and bare NF were provided in Figure S44 (Supporting Information). Overall, the Cr/NiBP exhibited exceptional HER/OER, repeatability, and stability due to its unique material properties, intrinsic activity, and corrosion resistance.

### 3.3 Overall Water Splitting (OWS) Performance

**Figure 5:**
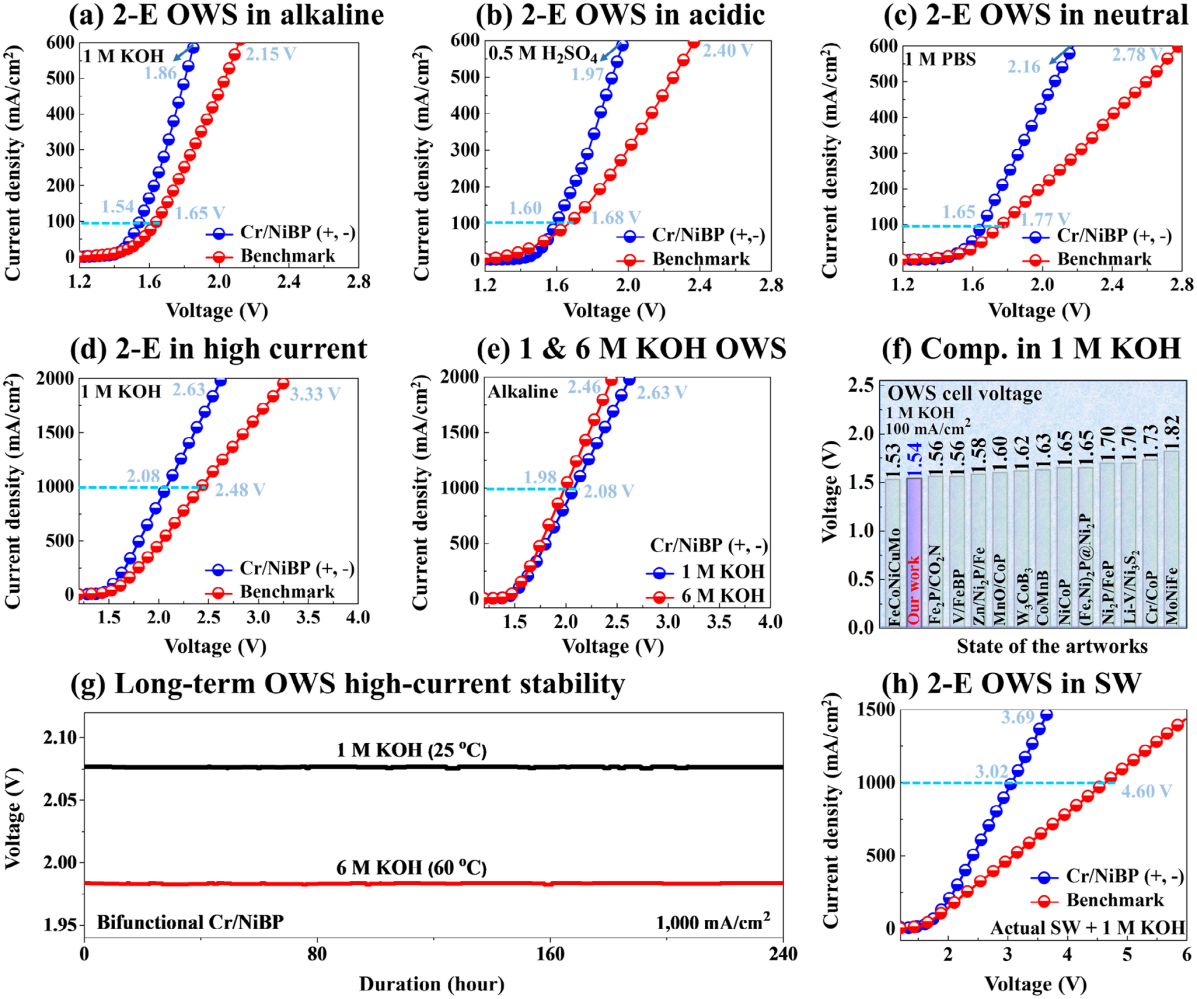
Two electrodes (2‐E) bifunctional Cr/NiBP (+) ǁ Cr/NiBP (‐) under various conditions. a–c) 2‐E performance of Cr/NiBP (+, −) as compared to the benchmarks RuO_2_ (+) ǁ Pt/C (−) in 1 m KOH, 0.5 m H_2_SO_4_, and 1 m PBS. d) High current OWS in 1 m KOH. e) 2‐E high current comparisons in 1 and 6 m KOH. e) 2‐E comparison with the reported electrodes (Related to Table S6, Supporting Information). f) Long‐term stability at 1000 mA cm^−2^ in 1 and 6 m KOH. g) 2‐E OWS in real seawater (SW) + 1 m KOH.

**Figure** **5** shows the two‐electrode (2‐E) OWS application of the Cr/NiBP bifunctional system. The Cr/NiBP is used as both anode and cathode (Cr/NiBP (+) ǁ Cr/NiBP (−)) and compared with the benchmark of RuO_2_ (+) ǁ Pt/C (−). The Cr/NiBP (+, −) surpassed the benchmark system of RuO_2_ (+) ǁ Pt/C (−) in all pH media as illustrated in Figure 5a,c. The Cr/NiBP (+, −) demonstrated low cell voltages of 1.86, 1.97, and 2.16 V at 600 mA cm^−2^ respectively in 1 m KOH, 0.5 m H_2_SO_4_ and 1 m PBS as compared with the benchmark of 2.15, 2.40, and 2.78 V. The different pH performances of Cr/NiBP are summarized in Table S4 (Supporting Information). The high OER capability of Cr/NiBP plays a key role in obtaining significantly enhanced OWS. Additionally, the neutral media performance of Cr/NiBP is the second best at 100 mA cm^−2^ in 1 m PBS as compared to the state‐of‐the‐art works in Table S5 (Supporting Information). Generally, the industry prefers high‐current electrolysis for large‐scale H_2_ production. The LSV curves exceeded up to 2000 mA cm^−2^ high current density as shown in Figure 5d.

The Cr/NiBP (+, −) exhibited a low cell voltage of 2.63 V at 2000 mA cm^−2^ in 1 M KOH, which is significantly better than the benchmark value of 3.33 V. Alongside high current operations, the industry employs a high concentration of alkaline solution and elevated temperatures to boost production rates. Impressively, the Cr/NiBP exhibited an ultra‐low cell voltage of 2.46 V at 2000 mA cm^−2^ in 6 m KOH at 60 °C as seen in Figure 5e, demonstrating its strong potential for industrial applications. The accelerated WS capabilities of Cr/NiBP can be attributed to higher charge transfer rates and favorable reaction thermodynamics.^[^ ^61^, ^62^ ^]^ The high concentration of alkaline water (6 m KOH) provides more ions, leading to improved ionic conductivity, while the elevated temperatures supply the energy to overcome activation barriers.^[^ ^61^, ^62^ ^]^ The Cr/NiBP exhibited highly comparable and even better OWS efficiency than other previously reported catalysts in Figure 5f and Table S6 (Supporting Information). After successful Cr doping, the 2‐E polarization curves clearly demonstrated that the cell voltage of Cr/NiBP was largely reduced (≈1040 mV at 2000 mA cm^−2^) as shown in Figure S47 (Supporting Information). The multi‐step long‐term stability of Cr/NiBP (+, −) was evaluated at 1000 mA cm^−2^ in 1 and 6 m KOH at 60 °C over 240 h (over 10 days) in Figure 5g. The stable operation in harsh conditions indicated that Cr/NiBP has the potential and strong anti‐corrosion ability to fulfill industrial requirements. Additionally, the Cr/NiBP (+, −) exhibited stable steady‐state currents at different applied voltages (Figure S48, Supporting Information), indicating efficient gas bubble release. After 2000 CV cycles, the 2‐E LSV curves exhibited nearly identical current densities in both 1 and 6 m KOH (Figure S49, Supporting Information), indicating excellent repeatability. An additional stability test can be found in Figure S50 (Supporting Information). The high‐current stability of Cr/NiBP is compared with state‐of‐the‐art works in Table S7 (Supporting Information). The promising stability of the Cr/NiBP catalyst can be attributed to its robust morphology, strong Cr‐NiBP interactions, and large ECSA.^[^ ^63^ ^]^

Inspired by its superior performance in neutral media (1 m PBS), the 2‐E WS was further evaluated in alkaline natural seawater (SW + 1 m KOH) in Figure 5h. The Cr/NiBP exhibited significantly higher seawater performance up to 1500 mA cm^−2^ as compared to the benchmark, indicating promising natural water electrolysis capability. It achieved high current densities of 1000 and 1500 mA cm^−2^ at applied voltages of 3.02 and 3.69 V, respectively. The observed seawater performance was lower than deionized (DI) water. The seawater was alkalized with 1 m KOH to improve conductivity and faster reaction. Seawater splitting is particularly challenging due to the presence of various ions (Ca^2^⁺, Mg^2^⁺, Na⁺, Cl⁻, F⁻, and Br⁻), salts (e.g., NaCl ≈ 0.5 m), and contaminants such as dust, bacteria, and viruses.^[^ ^64^ ^]^ These impurities can lead to the loss of active species, competitive side reactions, and decreased performance of TM‐based electrocatalysts.^[^ ^64^ ^]^ The insoluble white perception observed during electrolysis, which may be due to the formation of Ca(OH)_2_/Mg(OH)_2_.^[^ ^64^ ^]^ Similarly, the Cr/NiBP (+, −) also showed superior performance as compared to the benchmark in bare seawater (SW) and alkaline river water (RW + 1 m KOH) as shown in Figure S51 (Supporting Information). Additionally, the Cr/NiBP exhibited stable multi‐step operation at current densities of 100 and 500 mA cm^−2^ in SW + 1 m KOH for over 24 h in Figure S52 (Supporting Information), demonstrating strong corrosion resistance and surface stability against various ions, chemicals and biological substances.

### 3.4 Hybrid Electrolysis and Post‐Stability Characteristics

**Figure 6:**
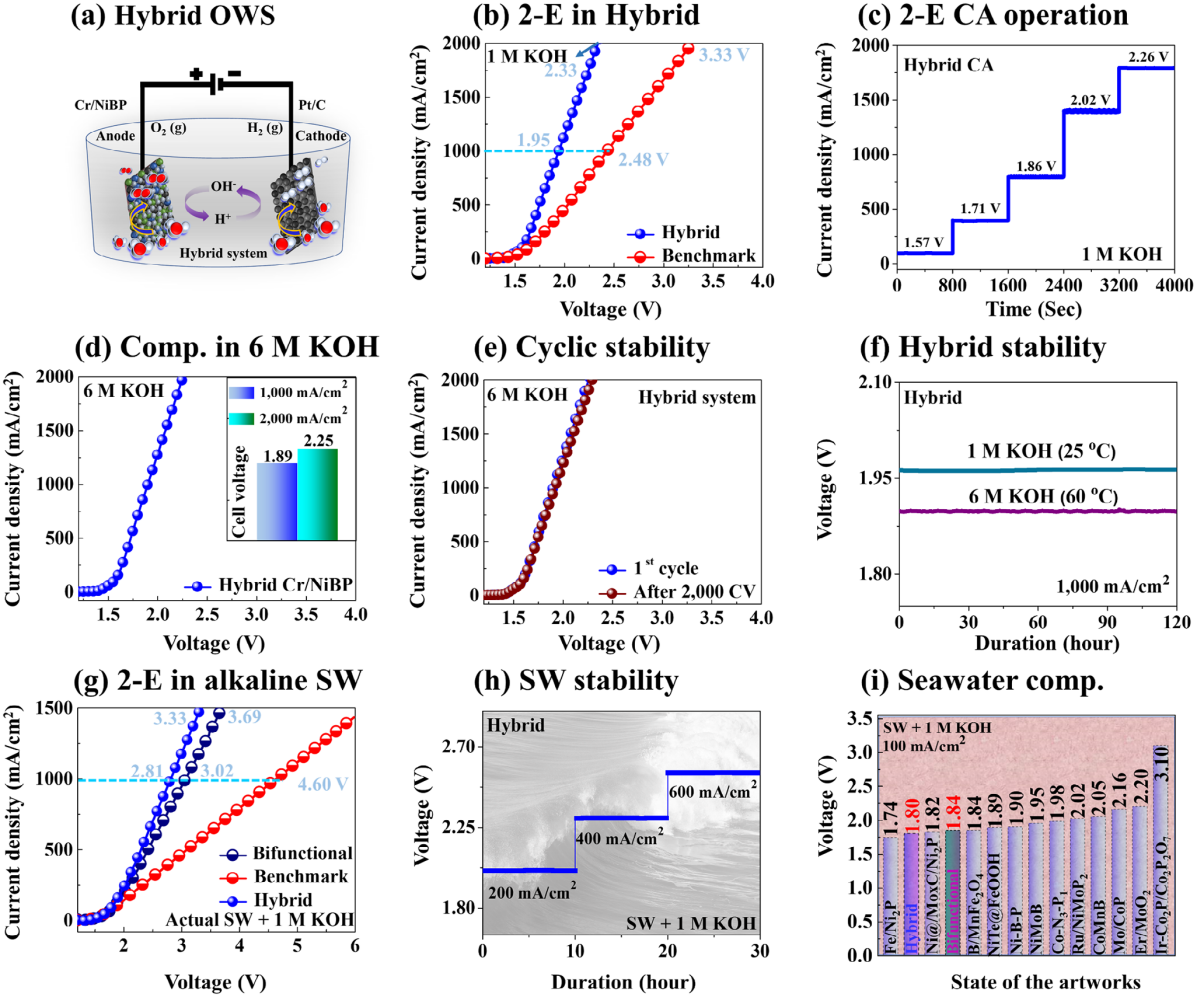
2‐E performance hybrid system of Cr/NiBP (+) ǁ Pt/C (‐) as compared to the benchmarks in alkaline and seawater conditions. a) Schematic diagram of the hybrid system. b) 2‐E hybrid in 1 m KOH. c) Hybrid CA in 1 m KOH. d) Cyclic repeatability. e) 2‐E OWS comparisons in 6 m KOH. f) Hybrid stability in 1 and 6 m KOH. g) 2‐E OWS performance in alkaline mixed actual seawater (SW + 1 m KOH). h) SW stability. i) Comparison of our electrode system with the reported works in SW + 1 m KOH (Related to Table S10, Supporting Information).

**Figure** **6** shows the 2‐E hybrid OWS performance utilizing Cr/NiBP as anode (+) and Pt/C as cathode (−) for advanced industrial‐scale H_2_ production. The Cr/NiBP exhibited ≈320 mV lower overpotential at 600 mA cm^−^^2^ than RuO_2_, which is the bottleneck of OWS. The Cr/NiBP was adapted as an anode to replace RuO_2_ for hybrid system construction as illustrated in Figure 6a. Initially, the hybrid configuration of Cr/NiBP (+) ǁ Pt/C (−) showed largely improved activity and outperformed the benchmark of RuO_2_ (+) ǁ Pt/C (−) in all pH media in Figure S53 (Supporting Information). Coupling with Pt/C, the hybrid demonstrated cell voltages of 1.78, 2.01, and 2.14 V at 600 mA cm^−2^ respectively in 1 m KOH, 0.5 m H_2_SO_4_, and 1 m PBS, which is lower than bifunctional Cr/NiBP (1.86, 1.97, and 2.16 V) and benchmark (2.15, 2.40, and 2.78 V) as summarized in Table S8 (Supporting Information). The hybrid exhibited significantly lower cell voltages as compared to the others, suggesting reduced energy consumption. Further, the 2‐E LSV curve increased up to 2000 mA cm^−2^ high current density in Figure 6b. The hybrid exhibited a super‐low cell voltage of 2.33 V at 2000 mA cm^−2^ in 1 m KOH, outperforming the benchmark value of 3.33 V. This result highlights the efficient replacement of RuO_2_ as an anode material. The hybrid showed stable *V–t* curve response at 1.57, 1.71, 1.86, 2.02, and 2.26 V in 1 m KOH in Figure 6c. This stability is consistent with the LSV currents as seen in Figure S54 (Supporting Information). Nevertheless, the hybrid demonstrated ultra‐low cell voltages of 1.89 and 2.25 V respectively at 1000 and 2000 mA cm^−2^ in 6 m KOH + 60 °C in Figure 6d. This compares to the 2.46 V cell voltage achieved by the bifunctional Cr/NiBP ǁ Cr/NiBP configuration. The Cr/NiBP material system exhibits superior catalytic performance, as evidenced by its comparable activity of the bifunctional in high‐concentration alkaline electrolytes, although the hybrid works better in industrial conditions. The summarized high‐current performances can be found in Table S9 (Supporting Information). Further, the hybrid LSV curves demonstrated negligible potential shift after 2000 CV cycles over 20 h as seen in Figure 6e and Figure S55 (Supporting Information), indicating excellent cyclic repeatability. The multi‐step long‐term stability of the hybrid system was also evaluated at 1000 mA cm^−2^ in both 1 and 6 m KOH (60 °C) in Figure 6f, which exhibited almost no change in potential even after 120 h. These results confirm the potential of Cr/NiBP as a promising, noble‐metal‐free, and cost‐effective alternative to RuO_2_. Finally, the hybrid performance was investigated in SW + 1 m KOH as shown in Figure 6g and it showed relatively higher activity than benchmark/bifunctional. The hybrid required only 3.33 V to achieve 1500 mA cm^−2^ current density, indicating the advanced capability. The hybrid showed reaction stability at 200, 400, and 600 mA cm^−2^ in SW + 1 m KOH for 30 h as shown in Figure 6h, suggesting strong robustness for seawater. Additionally, the hybrid also outperformed the benchmark in SW and RW + 1 m KOH as shown in Figure S56 (Supporting Information). Finally, the seawater performance was compared with the state‐of‐the‐art works in SW + 1 m KOH as seen in Figure 6i and Table S10 (Supporting Information). The Cr/NiBP configuration demonstrated highly comparable activity, indicating one of the best multi‐functional electrocatalysts.

Further, the post‐stability physical and electrochemical situ characterizations were conducted after 1000 mA cm^−2^ stability for 30 h using SEM, Raman, XRD, XPS, and LSV as shown in Figures S57–S62 (Supporting Information). In this work, the Cr/NiBP exhibits advanced anodic OER characteristics. All the post‐stability physical characterizations were investigated on anodic Cr/NiBP. After stability, the Cr/NiBP maintained its initial structure with surface roughness as shown in Figure S57a,b (Supporting Information), indicating morphological stability. The roughness likely arises from bubble formation and oxide growth at the electrode–electrolyte interface.^[^ ^32^, ^33^ ^]^ Further, the Raman comparison revealed reduced peak intensity and an additional peak formation at ≈570 cm^−1^ as seen in Figure S58 (Supporting Information). Raman analysis confirmed the formation of the oxyhydroxide (NiOOH) phase at the anode, resulting from increased valence state and chemical bond transitions, which can act as an active site and corrosion resist layer (amorphous).^[^ ^65^ ^]^ The XRD of Cr/NiBP further exhibited the disappearance of several diffraction peaks in Figure S59 (Supporting Information). This is likely due to the local crystal phase transformation, adsorption of O‐containing intermediates (M─O, M─OH, M─OOH), amorphous oxide layer formation, and continuous reactions in the alkaline environment.^[^ ^33^, ^65^ ^]^ This chemically inert oxide layer can act as a physical barrier, effectively preventing further oxidation and corrosion of the deposited materials.^[^ ^33^, ^65^ ^]^ The full scan XPS profile confirmed the existence of all active elements Cr, Ni, B, and P after stability in Figure S60 (Supporting Information). In addition, the high‐resolution XPS spectra of Cr 2p_3/2_, Ni 2p_1/2_, B 1s, and P 2p_1/2_ maintain their original structure in Figure S61a,d (Supporting Information), while the Ni 2p spectra exhibit evident increases in peak areas associated with the inevitable formation of NiOOH. Overall, partial surface oxidation during redox reactions led to decreased elemental peak intensities and increased oxide peaks.^[^ ^33^, ^65^ ^]^ Finally, the post‐stability HER, OER, and OWS of Cr/NiBP (electrode serve as anode: OER, cathode: HER) showed nearly identical performance in Figure S62a,d (Supporting Information), confirming the electrochemical robustness. Therefore, the superior stability of the Cr/NiBP electrode can be attributed to strong interactions between active components, protection layer formation, and enhanced corrosion resistance.

## 4 Conclusion

In summary, we have successfully introduced a novel and cost‐effective approach to preparing Cr/NiBP as an efficient and stable electrocatalyst for overall water splitting. After Cr nanocluster incorporation, the HER, OER, and OWS capability of Cr/NiBP MS are greatly improved. The Cr/NiBP exhibited low HER/OER overpotentials of 76 and 250 mV at 100 mA cm^−2^ in 1 m KOH with robust stability, positioning it among the top state‐of‐the‐art electrocatalysts. The superior performance of the Cr/NiBP electrode can be attributed to improved conductivity, abundant active sites, and large ECSA. The synergistic effect of the multi‐component electrode materials optimized the adsorption/desorption rates, leading to enhanced formation of reaction intermediates and accelerated water dissociation kinetics. The bifunctional application of Cr/NiBP as both anode and cathode required very low voltage to achieve high current under various conditions. Remarkably, the Cr/NiBP demonstrated super‐low turnover cell voltages of 1.54 V at 100 mA cm^−2^ in 1 m KOH and outperformed the benchmark of RuO_2_ (+) ǁ Pt/C (−) up to 2000 mA cm^−2^. Moreover, it maintained long‐term stability at 1000 mA cm^−2^ in both 1 and 6 m KOH (60 °C) harsh industrial conditions over 240 h with strong anti‐corrosion properties. Along with the superior OER, the Cr/NiBP hybrid system exhibited ultra‐low cell voltage of 2.25 V at 2000 mA cm^−2^ in 6 m KOH at 60 °C and operated at 1000 mA cm^−2^ over 120 h, confirming a cost‐effective alternative of RuO_2_. This work provides new insights and clear guidance for better design of highly efficient electrode materials to replace traditional benchmarks.

## Conflict of Interest

The authors declare no conflict of interest.

## Author Contributions

M.A.H. dealt with the investigation, data curation, visualization, and methodology, and wrote the original draft. S.L. dealt with the investigation and data curation. S.A.D. dealt with the investigation and data curation. M.H.J. dealt with the investigation and data curation. R.M. dealt with the investigation and data curation. J.‐H.J. dealt with the supervision, methodology, and investigation. J.L. dealt with the conceptualization, supervision, and funding acquisition and did the review and editing.

## Supporting information



Supporting Information

## Data Availability

The data that support the findings of this study are available from the corresponding author upon reasonable request.

## References

[1] G. Zhao , W. Ma , X. Wang , Y. Xing , S. Hao , X. Xu , Adv. Powder Mater. 2022, 1, 100008.

[2] X. Xu , K. Guo , J. Sun , X. Yu , X. Miao , W. Lu , L. Jiao , Adv. Funct. Mater. 2024, 34, 2400397.

[3] X. Zhou , Y. Mo , F. Yu , L. Liao , X. Yong , F. Zhang , D. Li , Q. Zhou , T. Sheng , H. Zhou , Adv. Funct. Mater. 2022, 33, 2209465.

[4] L. Zhu , H. Zhang , A. Zhang , T. Tian , Y. Shen , M. Wu , N. Li , H. Tang , Adv. Powder Mater. 2024, 3, 100203.

[5] X. Li , L. Zhao , J. Yu , X. Liu , X. Zhang , H. Liu , W. Zhou , Nano‐Micro Lett. 2020, 12, 131. 10.1007/s40820-020-00469-3 PMC7770753 34138146

[6] S. L. D. Nicole , Y. Li , W. Xie , G. Wang , J. M. Lee , Small 2023, 19, 2206844. 10.1002/smll.202206844 36642855

[7] Y. Han , J. Wang , Y. Liu , T. Li , T. Wang , X. Li , X. Ye , G. Li , J. Li , W. Hu , Y. Deng , Carbon Neutralization 2024, 3, 172.

[8] K. Wang , Y. Guo , Z. Chen , D. Wu , S. Zhang , B. Yang , J. Zhang , InfoMat 2022, 4, e12251.

[9] M. Chen , N. Kitiphatpiboon , C. Feng , A. Abudula , Y. Ma , G. Guan , eScience 2023, 3, 100111.

[10] Y. Li , X. Yu , J. Gao , Y. Ma , Chem. Eng. J. 2023, 470, 144373.

[11] Y. Dong , Z. Deng , H. Zhang , G. Liu , X. Wang , Nano Lett. 2023, 23, 9087. 37747850 10.1021/acs.nanolett.3c02940

[12] R. Mandavkar , A. Habib , S. Lin , R. Kulkarni , S. Burse , J. Jeong , J. Lee , Appl. Mater. Today 2022, 29, 101579.

[13] N. Wang , L. Ji , Y. Zhai , J. Colloid Interface Sci. 2024, 669, 53. 38705112 10.1016/j.jcis.2024.04.197

[14] Z. Li , X. Wu , X. Jiang , B. Shen , Z. Teng , D. Sun , G. Fu , Y. Tang , Adv. Powder Mater. 2022, 1, 100020.

[15] L. Li , L. Zhang , L. Gou , S. Wei , X. Hou , L. Wu , Chem. Eng. J. 2023, 454, 140292.

[16] D. Li , L. Ding , Q. Zhao , F. Yang , S. Zhang , Appl. Energy 2024, 356, 122369.

[17] L. Kumar , B. Antil , A. Kumar , M. R. Das , O. López‐Estrada , S. Siahrostami , S. Deka , ACS Appl. Mater. Interfaces 2023, 15, 54446. 37970629 10.1021/acsami.3c11947

[18] K. Chang , D. T. Tran , J. Wang , K. Dong , S. Prabhakaran , D. H. Kim , N. H. Kim , J. H. Lee , Appl. Catal., B 2023, 338, 123016.

[19] S. Lin , M. A. Habib , R. Mandavkar , R. Kulkarni , S. Burse , Y. U. Chung , C. Liu , Z. Wang , S. Lin , J. H. Jeong , J. Lee , Adv. Sustainable Syst. 2022, 6, 2200213.

[20] X. Lv , S. Wan , T. Mou , X. Han , Y. Zhang , Z. Wang , X. Tao , Adv. Funct. Mater. 2023, 33, 2205161.

[21] K. Bhunia , M. Chandra , S. K Sharma , D. Pradhan , S. J. Kim , Coord. Chem. Rev. 2023, 478, 214956.

[22] S. Perumal , J. Seo , Int. J. Hydrogen Energy 2023, 48, 22009.

[23] W. Hao , D. Yao , Q. Xu , R. Wang , C. Zhang , Y. Guo , R. Sun , M. Huang , Z. Chen , Appl. Catal., B 2021, 292, 120188.

[24] R. Qin , L. Chi , C. Han , W. Wang , Y. Li , C. Xie , L. Zhao , X. Lang , Q. Jiang , J. Colloid Interface Sci. 2024, 664, 13. 38458051 10.1016/j.jcis.2024.02.204

[25] D. Li , Z. Li , R. Zou , G. Shi , Y. Huang , W. Yang , W. Yang , C. Liu , X. Peng , Appl. Catal., B 2022, 307, 121170.

[26] H. Chu , P. Feng , B. Jin , G. Ye , S. Cui , M. Zheng , G. X. Zhang , M. Yang , Chem. Eng. J. 2022, 433, 133523.

[27] A. Sivanantham , H. Lee , S. W. Hwang , H. U. Lee , S. B. Cho , B. Ahn , I. S. Cho , Adv. Funct. Mater. 2023, 33, 2301153.

[28] X. Mu , K. Wang , K. Lv , B. Feng , X. Yu , L. Li , X. Zhang , X. Yang , Z. Lu , ACS Appl. Mater. Interfaces 2023, 15, 16552. 36960922 10.1021/acsami.2c18799

[29] Q. Wang , R. He , F. Yang , X. Tian , H. Sui , L. Feng , Chem. Eng. J. 2023, 456, 141056.

[30] Q. Zhou , G. Song , J. Zou , S. Luo , A. Meng , Z. Li , Int. J. Hydrogen Energy 2023, 48, 15921.

[31] M. Ahasan Habib , R. Mandavkar , S. Lin , S. Burse , T. Khalid , M. Hasan Joni , J. H. Jeong , J. Lee , Chem. Eng. J. 2023, 462, 142177.

[32] M. A. Habib , S. Lin , M. H. Joni , S. A. Dristy , R. Mandavkar , J.‐H. Jeong , J. Lee , J. Energy Chem. 2025, 100, 397.

[33] S. Lin , R. Mandavkar , M. A. Habib , S. A. Dristy , M. H. Joni , J.‐H. Jeong , J. Lee , J. Colloid Interface Sci. 2025, 677, 587. 39116558 10.1016/j.jcis.2024.08.009

[34] W. Li , Y. Jiang , Y. Li , Q. Gao , W. Shen , Y. Jiang , R. He , M. Li , Chem. Eng. J. 2021, 425, 130651.

[35] Y. Wu , X. Tao , Y. Qing , H. Xu , F. Yang , S. Luo , C. Tian , M. Liu , X. Lu , Adv. Mater. 2019, 31, 1900178. 10.1002/adma.201900178 30791164

[36] R. Müller , J. Fuhrmann , M. Landstorfer , J. Electrochem. Soc. 2020, 167, 106512.

[37] X. Ding , R. Jiang , J. Wu , M. Xing , Z. Qiao , X. Zeng , S. Wang , D. Cao , Adv. Funct. Mater. 2023, 33, 2306786.

[38] J. Liu , G. Qian , T. Yu , J. Chen , C. Zhu , Y. Li , J. He , L. Luo , S. Yin , Chem. Eng. J. 2022, 431, 134247.

[39] D. Briggs , in Handbook of Adhesion, 2nd ed., Wiley, Hoboken 2005, Ch22.

[40] S. Yang , J. Y. Zhu , X. N. Chen , M. J. Huang , S. H. Cai , J. Y. Han , J. S Li , Appl. Catal., B 2022, 304, 120914.

[41] Y. Wang , G. Qian , Q. Xu , H. Zhang , F. Shen , L. Luo , S. Yin , Appl. Catal., B 2021, 286, 119881.

[42] S. Perumal , M. K. A. Mohammed , M. Govindasamy , A. A. Alothman , M. Ouladsmane , R. Ganesan , Int. J. Hydrogen Energy 2024, 54, 652.

[43] B. Zhang , F. Yang , X. Liu , N. Wu , S. Che , Y. Li , Appl. Catal., B 2021, 298, 120494.

[44] Y. Chen , J. Wang , Z. Yu , Y. Hou , R. Jiang , M. Wang , J. Huang , J. Chen , Y. Zhang , H. Zhu , Appl. Catal., B 2022, 307, 121151.

[45] M. A. Habib , R. Mandavkar , S. Burse , S. Lin , R. Kulkarni , C. S. Patil , J. H. Jeong , J. Lee , Mater. Today Energy 2022, 26, 101021.

[46] J. Y. Wang , T. Ouyang , N. Li , T. Ma , Z. Q. Liu , Sci. Bull. 2018, 63, 1130. 10.1016/j.scib.2018.07.008 36658993

[47] N. Yao , P. Li , Z. Zhou , Y. Zhao , G. Cheng , S. Chen , W. Luo , Adv. Energy Mater. 2019, 9, 902449.

[48] L. Zhang , H. Zhao , S. Xu , Q. Liu , T. Li , Y. Luo , S. Gao , X. Shi , A. M. Asiri , X. Sun , Small Struct. 2021, 2, 2000048.

[49] M. Lao , P. Li , Y. Jiang , H. Pan , S. X. Dou , W. Sun , Nano Energy 2022, 98, 107231.

[50] I. Pathak , A. Muthurasu , D. Acharya , K. Chhetri , B. Dahal , Y. R. Rosyara , T. Kim , T. H. Ko , H. Y. Kim , J. Mater. Chem. A 2024, 12, 17544.

[51] L. C. Zhang , H. Chen , G. R. Hou , L. Z. Zhang , Q. L. Li , Y. K. Wu , M. Xu , S. J. Bao , Chem. Commun. 2019, 56, 257. 10.1039/c9cc08032e 31803880

[52] L. Jia , G. Du , D. Han , Y. Wang , W. Zhao , S. Chen , Q. Su , J. Colloid Interface Sci. 2024, 653, 246. 37716304 10.1016/j.jcis.2023.09.041

[53] Y. Yu , J. Zhou , Z. Sun , Adv. Funct. Mater. 2020, 30, 2000570.

[54] L. Zhang , L. Li , J. Liang , X. Fan , X. He , J. Chen , J. Li , Z. Li , Z. Cai , S. Sun , D. Zheng , Y. Luo , H. Yan , Q. Liu , A. A. Alshehri , X. Guo , X. Sun , B. Ying , Inorg. Chem. Front. 2023, 10, 2766.

[55] Y. Li , Z. Zhu , Y. L. Zhong , Y. Jin , P. Saha , Q. Cheng , J. Power Sources 2024, 614, 234969.

[56] C. L. Huang , Y. G. Lin , C. L. Chiang , C. K. Peng , D. Senthil Raja , C. T. Hsieh , Y. A. Chen , S. Q. Chang , Y. X. Yeh , S. Y. Lu , Appl. Catal., B 2023, 320, 122016.

[57] X. Wang , H. Yao , C. Zhang , C. Li , K. Tong , M. Gu , Z. Cao , M. Huang , H. Jiang , Adv. Funct. Mater. 2023, 33, 2210728.

[58] X. Hu , G. Luo , X. Guo , Q. Zhao , R. Wang , G. Huang , B. Jiang , C. Xu , F. Pan , Sci. Bull. 2021, 66, 708. 10.1016/j.scib.2020.11.009 36654446

[59] S. Meng , S. Sun , Y. Liu , Y. Lu , M. Chen , J. Colloid Interface Sci. 2022, 624, 433. 35667205 10.1016/j.jcis.2022.04.141

[60] M. Arif , G. Yasin , M. Shakeel , M. A. Mushtaq , W. Ye , X. Fang , S. Ji , D. Yan , J. Energy Chem. 2021, 58, 237.

[61] J. T. Ren , L. Chen , H. Y. Wang , W. W. Tian , X. L. Song , Q. H. Kong , Z. Y. Yuan , ACS Catal. 2023, 13, 9792.

[62] J. N. Hausmann , B. Traynor , R. J. Myers , M. Driess , P. W. Menezes , ACS Energy Lett. 2021, 6, 3567.

[63] Q. N. Ha , N. Susanto Gultom , C. H. Yeh , D. H. Kuo , Chem. Eng. J. 2023, 472, 144931.

[64] F. Zhang , Y. Liu , F. Yu , H. Pang , X. Zhou , D. Li , W. Ma , Q. Zhou , Y. Mo , H. Zhou , ACS Nano 2023, 17, 1681. 10.1021/acsnano.2c11844 36594437

[65] N. S. Gultom , T. S. Chen , M. Z. Silitonga , D. H. Kuo , Appl. Catal., B 2023, 322, 122103.

