# Supplementary material for: Chromium‐Doped NiBP Micro‐Sphere Electrocatalysts for Green Hydrogen Production under Industrial Operational Conditions
Source: Small Methods. 2025 Jan 19;9(7):2401939. doi: 10.1002/smtd.202401939 (PMC12285639; doi:10.1002/smtd.202401939)
Supplement: Supplementary file 1 — Supporting Information [file SMTD-9-2401939-s001.docx]

**Supporting Information**

**Chromium Doped NiBP Micro-sphere Electrocatalysts for Green Hydrogen Production Under Industrial Operational Conditions**

Md Ahasan Habib, Shusen Lin, Sumiya Akter Dristy, Mehedi Hasan Joni, Rutuja Mandavkar, Jae-Hun Jeong^*^ and Jihoon Lee^**^

*Department of Electronic Engineering, College of Electronics and Information, Kwangwoon University, Nowon-gu Seoul, 01897, South Korea.*

* Correspondence e-mail: myloveofjh@gmail.com (J. Jeong)^*^, jihoonlee@kw.ac.kr (J. Lee)^**^

**Supplementary table of contents**

**S-1. Detailed supplementary experimental section**

**S-2. Optimization of Cr/NiBP micro sphere electrode**

**S-3. Analysis on the best Cr/NiBP electrode**

**S-4. Supplementary tables**

**S-5. SI Reference**

Contents

[**S-1. Detailed experimental supplementary** 6](#_Toc185892045)

[**1.1. Synthesis of Cr/NiBP micro sphere (MS) electrocatalyst** 6](#_Toc185892046)

[**1.2. Electrochemical characterizations** 9](#_Toc185892047)

[**1.3. Physical characterizations** 12](#_Toc185892048)

[**S-1.4. Materials** 13](#_Toc185892049)

[**S-1.5. Fabrication of HER and OER benchmark electrodes** 13](#_Toc185892050)

[**S-1.6. NiBP micro sphere (MS) base electrode characterizations** 15](#_Toc185892051)

[**S-1.7. Raman analysis of Cr/NiBP** 17](#_Toc185892052)

[**S-1.8. X-ray diffraction (XRD) analysis of Cr/NiBP** 18](#_Toc185892053)

[**S-1.9. X-ray photoelectron spectroscopy (XPS) of Cr/NiBP** 19](#_Toc185892054)

[**S-1.10. Cyclic CV in the OER range of Cr/NiBP** 21](#_Toc185892055)

[**S-1.11. TOF calculation of Cr/NiBP MS** 22](#_Toc185892056)

[**S-1.12. Faradaic efficiency calculation of Cr/NiBP MS** 25](#_Toc185892057)

[**S-1.13. Cr doping effect** 27](#_Toc185892058)

[**S-2.1. Substrate and other necessary** 29](#_Toc185892059)

[**S-2.1.1. Block diagram of Cr/NiBP fabrication steps** 29](#_Toc185892060)

[**S-2.1.2. Characterization of bare Ni foam (NF)** 30](#_Toc185892061)

[**S-2.1.3. Scan rate variations on Cr/NiBP** 31](#_Toc185892062)

[**S-2.1.4. EIS applied voltage variations on Cr/NiBP** 32](#_Toc185892063)

[**S-2.1.5. HER benchmark electrode: Pt/C characterization** 33](#_Toc185892064)

[**S-2.1.6. OER benchmark electrode: RuO_2_ characterization** 34](#_Toc185892065)

[**S-2.2. NiBP base-electrode analysis** 35](#_Toc185892066)

[**S-2.2.1. NiBP base-electrode: SEM & EDS** 35](#_Toc185892067)

[**S-2.2.2. NiBP base-electrode: Raman & XRD** 36](#_Toc185892068)

[**S-2.2.3. NiBP base-electrode: HER/OER & EIS** 37](#_Toc185892069)

[**S-2.2.4. NiBP base-electrode: CV curves & current plot** 38](#_Toc185892070)

[**S-2.2.5. NiBP base-electrode: C_dl_ values & ECSA** 39](#_Toc185892071)

[**S-2.3. Optimization of Cr doping by hydrothermal reaction** 40](#_Toc185892072)

[**S-2.3.1. Cr doping: Cr concentration control** 40](#_Toc185892073)

[**S-2.3.1. Cr doping: Cr concentration control** 41](#_Toc185892074)

[**S-2.3.2. Cr doping: Reaction temperature control (at 12 hours)** 42](#_Toc185892075)

[**S-2.3.2. Cr doping: Reaction temperature control** 43](#_Toc185892076)

[**S-2.3.3. Cr doping: Reaction duration control (at 160 ^o^C)** 44](#_Toc185892077)

[**S-2.3.3. Cr doping: Reaction duration control (at 160 ^o^C)** 45](#_Toc185892078)

[**S-2.3.4. Cr doping: Reaction temperature control (for 8 h)** 46](#_Toc185892079)

[**S-2.3.4. Cr doping: Reaction temperature control (for 8 h)** 47](#_Toc185892080)

[**S-2.3.5. Cr doping: Cr con. control (at 160 ^o^C for 8 h)** 48](#_Toc185892081)

[**S-2.3.5. Cr doping: Cr con. control (at 160 ^o^C for 8 h)** 49](#_Toc185892082)

[**S-2.3.6. Post-annealing temperature optimization: SEM** 50](#_Toc185892083)

[**S-2.3.6. Post-annealing temperature optimization: LSV** 51](#_Toc185892084)

[**S-2.3.7. Post-annealing duration optimization: SEM** 52](#_Toc185892085)

[**S-2.3.7. Post-annealing duration optimization: EDS** 53](#_Toc185892086)

[**S-2.3.7. Post-annealing duration optimization: HER CV** 54](#_Toc185892087)

[**S-2.3.7. Post-annealing duration optimization: OER CV** 55](#_Toc185892088)

[**S-2.3.7. Post-annealing duration optimization: C_dl_** 56](#_Toc185892089)

[**S-2.3.7. Post-annealing duration optimization: ECSA** 57](#_Toc185892090)

[**S-2.4. NiBP and Cr/NiBP comparison** 58](#_Toc185892091)

[**S-2.4.1. NiBP and Cr/NiBP comparison: Raman** 58](#_Toc185892092)

[**S-2.4.2. NiBP and Cr/NiBP comparison: XRD** 59](#_Toc185892093)

[**S-2.4.3. NiBP and Cr/NiBP comparison: EIS** 60](#_Toc185892094)

[**S-2.4.4. NiBP and Cr/NiBP comparison: C_dl_ & ECSA** 61](#_Toc185892095)

[**S-2.4.5. NiBP and Cr/NiBP comparison: HER & OER** 62](#_Toc185892096)

[**S-2.4.6. NiBP and Cr/NiBP comparison: ECSA-normalized LSV** 63](#_Toc185892097)

[**S-3. Analysis on Cr/NiBP electrode** 64](#_Toc185892098)

[**S-3.1. Structural analysis on Cr/NiBP** 64](#_Toc185892099)

[**S-3.1.1. CV in OER: oxidation peak formation** 64](#_Toc185892100)

[**S-3.1.2. TEM analysis on Cr/NiBP** 65](#_Toc185892101)

[**S-3.1.3. EDS analysis on Cr/NiBP** 66](#_Toc185892102)

[**S-3.1.4. XRD pattern analysis on Cr/NiBP** 67](#_Toc185892103)

[**S-3.2. 3-E electrochemical analysis on Cr/NiBP** 68](#_Toc185892104)

[**S-3.2.1. TOF of Cr/NiBP in different pH solutions** 68](#_Toc185892105)

[**S-3.2.2. HER/OER CA performance & LSV comparison of Cr/NiBP** 69](#_Toc185892106)

[**S-3.2.3. HER/OER repeatability test of Cr/NiBP** 70](#_Toc185892107)

[**S-3.2.4. HER/OER stability test of Cr/NiBP** 71](#_Toc185892108)

[**S-3.2.5. HER/OER comparison of Cr/NiBP & NF** 72](#_Toc185892109)

[**S-3.2.6. Water–gas displacement for faradic efficiency** 73](#_Toc185892110)

[**S-3.2.7. Faradaic efficiency of Cr/NiBP electrode** 74](#_Toc185892111)

[**S-3.3. 2-E electrochemical analysis on bifunctional Cr/NiBP ǁ Cr/NiBP** 75](#_Toc185892112)

[**S-3.3.1. 2-E comparison on Cr/NiBP and NiBP** 75](#_Toc185892113)

[**S-3.3.2. 2-E CA and LSV comparison on Cr/NiBP** 76](#_Toc185892114)

[**S-3.3.3. 2-E repeatability test of bifunctional Cr/NiBP** 77](#_Toc185892115)

[**S-3.3.4. 2-E stability at industrial condition** 78](#_Toc185892116)

[**S-3.3.5. 2-E natural water performance of Cr/NiBP** 79](#_Toc185892117)

[**S-3.3.6. 2-E seawater stability of Cr/NiBP** 80](#_Toc185892118)

[**S-3.4. 2-E electrochemical analysis on hybrid (Cr/NiBP ǁ Pt/C) system** 81](#_Toc185892119)

[**S-3.4.1. 2-E OWS of hybrid system in different pH** 81](#_Toc185892120)

[**S-3.4.2. Hybrid CA and LSV comparison** 82](#_Toc185892121)

[**S-3.4.3. Hybrid repeatability in 6 M KOH** 83](#_Toc185892122)

[**S-3.4.4. Hybrid LSV performance in sea and river waters** 84](#_Toc185892123)

[**S-3.5. Before/after stability analysis on Cr/NiBP** 85](#_Toc185892124)

[**S-3.5.1. Before/post stability test: SEM** 85](#_Toc185892125)

[**S-3.5.2. Before/post stability test: Raman analysis** 86](#_Toc185892126)

[**S-3.5.3. Before/post stability test: XRD** 87](#_Toc185892127)

[**S-3.5.4. Before/post stability test: full scan XPS** 88](#_Toc185892128)

[**S-3.5.5. After stability: high-resolution XPS** 89](#_Toc185892129)

[**S-3.5.6. Before/post stability test: LSV** 90](#_Toc185892130)

[**S-4. Supplementary tables (Table S1- S10)** 91](#_Toc185892131)

[**S-5. SI references :** 101](#_Toc185892132)

**S-1. Detailed experimental supplementary**

**1.1. Synthesis of Cr/NiBP micro sphere (MS) electrocatalyst**

Figure S1 shows the schematic illustration of Cr/NiBP micro spherical (MS) electrocatalyst step-by-step synthesis process. First, NiBP micro-spherical (MS) template was prepared by a hydrothermal approach as seen in step 1. Then, the first-step annealing was applied to the NiBP MS electrocatalyst in step 2. Further, Cr-doping was achieved by an optimized second-step hydrothermal approach on NiBP as shown in step 3. Various doping parameters such as Cr doping concentration, doping reaction duration and doping temperature were systematically evaluated as specified in SI section S-2.3. Then, the second-step annealing was adapted on Cr/NiBP MS electrocatalyst to improve the crystallinity and electrochemical properties in step 4.

For the synthesis of NiBP MS, NiCl_2_^.^6H_2_O (1 mM) as Ni, H_3_BO_3_ (3.6 mM) as B**,** NaH_2_PO_2_.H_2_O (8.4 mM) as P along with CH_4_N_2_O (10 mM) and NH_4_F (10 mM) were adapted as precursors ^[1]^. The precursors were dissolved in 30 mL DI water under magnetic stirring to form a clear solution. This solution was transferred to a Teflon-lined hydrothermal reactor containing bare nickel foam (NF) and heated at 180 °C for 12 hours to synthesize NiBP/NF. The possible chemical reactions of NiBP template fabrication can be described as below:

NiCl_2_.6H_2_O + H_3_BO_3 +_ NaH_2_PO_2_∙H_2_O + CH_4_N_2_O + NH_4_F + n.H_2_O → Mixed solution (1)

NiCl_2_.6H_2_O → Ni^2+^ + 2Cl^-^ + 6H_2_O (1.1)

H_3_BO_3_  → B(OH)_3_ → B^3+^ + 3OH**^−^** (1.2)

NaH_2_PO_2_∙H_2_O → PH_3_ + Na_2_HPO_4_ + 2H_2_O →

PH_3_ + Na_2_HPO_4_ + 2H_2_O → P^3^**^−^** + HPO_4_^2-^ + 3H^+^ + 2Na^+^ + 2H_2_O (1.3)

CH_4_N_2_O + H_2_O → 2NH_3_ + CO_2_ ↑ → NH_3_ + H_2_O → NH_4_^+^ + OH^-^  (1.4)

NH_4_F → NH_4_^+^ + F^-^ (1.5)

Ni^2+^ + B^3+^ **+** P^3^**^−^** + +2Cl^-^ **+** 3H^+^ + 2Na^+^ + HPO_4_^2-^ + 2NH_4_^+^ + F**^−^** + 4OH**^−^** + 8H_2_O

→ NiBP structure + 2Cl**^−^ +**3H^+^ +2Na^+^ + HPO_4_^2^**^−^** + 3NH_4_^+^ + F**^−^** + 3OH**^−^** + 8H_2_O (2)

The mixed precursors for the hydrothermal reaction are listed in Eq. (1). The ionic states of all chemicals are shown in Eqs. 1.1 – 1.5. In brief, the NiCl_2_.6H_2_O can decompose to form Ni^2+^ for taking part in the reaction, while the byproduct Cl^-^ can increase solution conductivity in Eq. 1.1. The H_3_BO_3_ can dissociate into trivalent ionic state B^+3^ and hydroxyl (OH^-^) ions as seen in Eq. 1.2. The P^3−^ and HPO_4_^2−^ ions from P containing precursor can directly react with other elements as shown in Eq. 1.3 ^[2]^. The proton (H^+^) can generate H_2_ gas bubbles, contributing to the formation of a spherical morphology. Urea (CH_4_N_2_O) and ammonium fluoride (NH_4_F) were commonly adapted as morphology-controlling agents ^[3,4]^. The presence of NH_4_^+^ functional groups can facilitate bond formation and act as a crystallizing agent as seen in Eqs. 1.4 - 1.5 ^[4]^. Also, the electronegative fluoride (F^-^) can regulate the solution's conductivity and activate NF substrates. Finally, the overall synthesis of the NiBP template can be summarized by Eq. 2. After the synthesis reaction was completed under elevated temperature and pressure hydrothermal conditions, the prepared NiBP was washed with deionized water and air-dried. Before doping, the NiBP was annealed at 300 °C for 60 minutes. A detailed characterization of the NiBP electrode is provided in Section S-2.2. The morphological, elemental and electrochemical properties of the NiBP template were extensively characterized (Figs. S7 - S11) and discussed in SI text S-1.6. The NiBP sphere can be an excellent electro-active template for metal doping due to its multi-component synergistic effect and intrinsic HER/OER properties. For example, the Ni-based electrode can exhibit faster OER reaction intermediate formation with suitable valance states ^[5]^. The multi-bonding capabilities of boron (B) can allow for chemically functionalized configurations near metallic sites and offer preservation of active sites with strong anti-corrosion resistance ^[6,7]^. The B can alter the d-band center of transition metals to enhance HER/OER ^[8]^. The presence of P can synergistically enhance charge transfer capabilities and accelerate H_2_O dissociation towards improved HER ^[9]^. Also, oxidized P species can improve structural flexibility for longer stability.

The post-growth doping with metallic atoms, such as Ru, Mo, Cr, V, Co, etc. can significantly enhance performance by modifying electrochemical properties, increasing the number of active sites and shortening ion/electron diffusion pathways ^[10,11]^. Metal atom doping (MAD) can accelerate the decomposition of water into OH* and H*, facilitating the formation of reaction intermediates ^[12]^. A small amount of active metal inclusion can significantly improve catalytic performance and stability ^[13]^. Recently, chromium (Cr) has been considered an affordable dopant, catalytically active transition metal with electron-donating properties, low electronegativity and favorable charge transfer characteristics ^[14]^. On the other hand, Cr is known as a corrosion inhibitor, which can enhance long-term stability ^[15]^. The incorporation of Cr into the NiBP matrix can be a promising approach to enhance electrochemical performance. The relatively low electronegativity (lower than Ni 1.91) of Cr within the NiBP matrix may lead to a more polarized charge distribution ^[15]^. The polarization effect can optimize the binding energy for hydrogen intermediates, boosting HER performance ^[15]^. Additionally, creating more active sites within the NiBP structure can increase the electrochemically active surface area and enhance the adsorption of oxygen-containing species, leading to improved OER performance ^[14,16]^.

Once NiBP is ready, various Cr-doping (CrN_3_O_9_.9H_2_O as Cr) parameters concentration, reaction temperature and reaction duration were systematically optimized using 2^nd^ step hydrothermal reaction as shown in section S-2.3 with Figs. S12 – S21. Through systematic experimentation and parameter optimization, we identified optimal conditions for enhanced HER and OER. Among the optimized Cr/NiBP samples, the 0.4 mM Cr-doped sample prepared at 160 ^o^C for 8 hours in the 2^nd^ step hydrothermal reaction was determined to exhibit superior toward HER and OER. After Cr-doping, the Cr/NiBP was introduced 2^nd^ step post-annealing at 100 ^o^C for 30 min. The Cr atoms diffuse into the NiBP host lattice under elevated temperature and pressure, which can introduce various structural defects, dislocations and atomic mismatches within the crystal lattice. Annealing can significantly enhance the crystal quality through atomic diffusions and the reduction of various defects ^[17,18]^. Improved crystal quality can contribute to enhanced conductivity and superior performance in both HER/OER ^[18]^. The post-annealing temperature and duration control parameters can be found in section S-2.4.

**1.2. Electrochemical characterizations**

The electrochemical performance was assessed using an electrochemical analyzer workstation (Wizmac, South Korea). In the standard three-electrode configuration, the freshly prepared samples, graphite rod and Ag/AgCl were employed as working, counter and reference electrodes respectively for all HER/OER characterization. All LSV potentials are transformed to the reversible hydrogen electrode (RHE) scale. The HER potential window was selected between – 0.6 and 0.2 V vs RHE and the OER potential window was chosen between 1.1 and 2.2 V vs RHE, respectively. The Tafel slope was determined from linear-sweep voltammetry (LSV) curves using the Tafel equation, η = a + b log|j|, where b and j denoted the Tafel slope and current density, respectively. Various chronoamperometry (CA) at different applied potentials, LSV repeatability and long-term chronopotentiometry (CP) were conducted to evaluate the stability of Cr/NiBP. The long-term HER/OER stability test was performed using CP operation at 600 mA/cm^2^ for 200 hours in 1 M KOH. The LSV performance was plotted as received without iR drop correction. Generally, the iR compensation can enhance performance by mitigating the effects of solution resistance ^[19]^, which was not adapted in this work. All LSV electrochemical measurements were conducted in a standard three-electrode configuration. The samples were analyzed using a fixed cell setup. The measurement errors were minimized as much as possible to ensure consistent results. The HER/OER performances were evaluated in various pH electrolytes such as alkaline (1 M KOH), acidic (0.5 M H_2_SO_4_) and neutral (1 M PBS) water_,_ respectively.

To investigate the influence of scan rates, LSV measurements were performed at varying scan rates ranging from 2 ~ 10 mV/s as shown in Fig. S3. According to the Randles-Sevcik equation, the diffusion layer typically decreases in size at higher scan rates, leading to a correspondingly higher current response ^[20]^:

Peak current value, $i_{P}=0.446nFAC^{o}\left( \frac{nFvD_{o}}{RT} \right)^{1/2}$ (3)

Here, *n* is the number of transferred electrons in the redox process, F (C mol^−1^) is the faradaic constant

and *A* (cm^2^) is the active surface of the electrode. Also, the *D_o_* (cm^2^ s^−1^) is the chemical diffusion coefficient, *R* (JK^−1^mol^−1^) is the ideal gas constant and *T* (K) is the temperature. The peak current values (*i_P_*) can exhibit an exponential relationship with the square root of scan rate *v* (mV/s). Consequently, the higher scan rates gradually lead to increased overpotentials. On the other hand, an excessively high scan rate can lead to excessively large double-layer capacitance, which can negatively impact performance ^[19]^. The 5 mV/s scan rate applied sample exhibited the highest HER/OER performance as shown in Figs. S3(a) – S3(b). The scan rate over 5 mV/s resulted in higher overpotential. Further, the 5 mV/s exhibited the highest kinetics and lowest slope values as shown in Figs. 3(a-1) – 3(b-1). Therefore, the 5 mV s^-1^ scan rate was commonly adopted for all LSV measurements.

Electrochemical impedance spectroscopy (EIS) was recorded at a current density of 20 mA/cm^2^ over a frequency range of 100 kHz to 0.1 Hz with 5 mV amplitude to evaluate the charge transfer resistance. EIS measurements were conducted with applied voltage variation at fixed current to evaluate (R_ct_) values as depicted in Fig. S4. The semicircular arc observed in the EIS curves corresponds to the charge transfer resistance (R_ct_), which reflects the rate of electron transfer under specific conditions ^[21]^. Higher applied voltages resulted in lower R_ct_ values as shown in Figs. S4(a) – S4(b). Higher-performing samples may exhibit low R_ct_ values at fixed applied voltage. To obtain a more accurate representation of the overall R_ct_ trend among different samples, EIS measurements were conducted at 20 mA/cm^2^ current density corresponding voltage. In the equivalent circuit model for EIS tests, R_s_, R_ct_ and CPE represent the solution resistance, charge transfer resistance and constant phase element (CPE), respectively. The CPE is used to represent the double-layer capacitance of solid electrode in the real-world situation ^[22]^. Cyclic voltammetry (CV) measurements were conducted at scan rates ranging from 40 to 180 mV/s within the potential window of 0.1 and 0.3 V vs. RHE for HER and 1.04 and 1.14 V for OER, respectively. The electrochemical double-layer capacitance (C_dl_) was obtained from the mid-range of CV curves in the non-faradaic region by plotting the slope, calculated as *J* = (*J_a_ - J_c_*) / 2 ^[23]^. The electrochemical surface area (ECSA) values were calculated from the C_dl_ values using the following equation: ECSA = $\frac{C_{dl}}{C_{s}} \times Sg$, where specific capacitance C_s_ = 0.04 mF/cm^2^ was commonly adapted from previous literature ^[24]^. The exposed active geometric surface area of the working electrode (Sg) was (0.5 cm * 0.5 cm) 0.25 cm^2^. The ECSA for each electrocatalyst was estimated from the electrochemical double-layer capacitance. While ECSA is a useful parameter for assessing intrinsic activity, it may not accurately reflect the true actual surface area or does not apply to practical applications ^[25]^. Nevertheless, ECSA can serve as a useful metric for comparing the relative number of active sites between different samples, and the measured values ​​can be influenced by experimental conditions ^[25,26]^. For intrinsic activity comparison, the before/after Cr doping HER and OER LSV curves were normalized ECSA-normalized. To exclude the influence of varying surface areas, normalize the current obtained from the LSV curve by dividing it by the ECSA ^[27,28]^. Detailed information regarding [turnover frequency](https://www.sciencedirect.com/topics/chemistry/turnover-frequency) (TOF) and faradaic efficiency (FE) measurements can be found in SI texts S-1.11 and S-1.12.

The two-electrode (2-E) performances were assessed in various pH electrolytes, including 1 M KOH, 0.5 M H_2_SO_4_ and 1 M PBS for overall water splitting (OWS). For the OWS system, the 2-E configurations were configured with anode (+) and cathode (-), such as bifunctional: Cr/NiBP (+) ǁ Cr/NiBP (-), hybrid: Cr/NiBP (+) ǁ Pt/C (-) and benchmark: RuO_2_ (+) ǁ Pt/C (-). High-current OWS performance was evaluated in 6 M KOH solution at 60 ^o^C elevated temperature for industrial applications. For natural water application, the 2-E OWS was performed in natural bare seawater (SW), alkaline river (RW + 1 M KOH) and alkaline seawater (SW + 1 M KOH). Multiple chronoamperometry (CA), chronopotentiometry (CP) and repeatability tests were conducted in various OWS operational conditions to evaluate the 2-E stability as specified in the related sections. The 2-E LSV repeatability was performed after 2,000 cycles of CV at a scan rate of 100 mV/s over 20 hours. The long-term stability test of both Cr/NiBP bifunctional and hybrid configurations was evaluated at 1,000 mA/cm^2^ high current density in both 1 M and 6 M KOH (60 ^o^C) over 240 and 120 hours, respectively, to meet industrial requirements.

**1.3. Physical characterizations**

Morphological and elemental investigations were performed using a scanning electronic microscope (SEM, COXEM, South Korea) and energy-dispersive X-ray spectroscope (EDS, Thermo Fisher, United States). For accurate quantitative EDS analysis, Cr/NiBP electrocatalyst particles were transferred from the nickel foam (NF) substrate to a carbon substrate. The interplanar lattice distance and crystal phase of the Cr/NiBP electrode were examined using transmission electron microscopy (TEM, JEM-2100F, JEOL, Japan). Multiple TEM measurements were performed from different regions to ensure the reproducibility and consistency of the observed interplanar distances. High-resolution TEM (HR-TEM) elemental mapping was performed on deep micron-scale regions of the material. The Raman measurements on various Cr/NiBP MS electrodes were performed by a NOST system (Nostoptiks, South Korea), which is composed of a 532 nm laser, spectrograph (ANDOR SR-500, United Kingdom), charge-coupled device (CCD) and other lenses. X-ray diffraction (XRD, D8 Advance, Bruker, the USA) was adapted to analyze the crystal structure and phase formation with a scanning rate of 2⁰ /min by the Cu Kα radiation (λ = 1.5406 Å). X-ray photoelectron spectroscopy (XPS, Thermo Fisher Scientific, USA) was conducted to investigate the chemical state and electronic structure of Cr/NiBP MS electrocatalyst at 1.5 KV under < 10−8 torr with the X-ray spot size of ~ 10 µm.

**S-1.4. Materials**

High porosity Ni foam (NF) was adapted as a substrate for Cr/NiBP MS electrocatalyst fabrication. The bare NF was chosen as substrate due to its affordable, conductivity and electro-favorable surface. The NF (3*2 cm) was pre-treated with 6 M HCl (hydrochloric acids) diluted solution and ultrasonicated for 20 min to remove potential impurities and oxides absorbed. The treated NF was washed with deionized (DI) water for 10 minutes to remove the acidic residuals and then dried in ambient. The morphological, elemental characterizations and electrochemical performance of bare NF are shown in Fig. S2. The pure nickel foam demonstrated exceptionally low electrochemical HER/OER activity in 1.0 M KOH, 0.5 M H_2_SO_4_ and 1.0 M PBS in Figs. S2(d) – S2(e). Nickel (Ⅱ) chloride hexahydrate (NiCl_2_.6H_2_O), boric acid (H_3_BO_3_), sodium hypophosphite monohydrate (NaH_2_PO_2_.H_2_O), urea (CH_4_N_2_O) and ammonium fluoride (NH_4_F) were used for NiBP fabrication. Chromium(ⅡI) nitrate nonahydrate (CrN_3_O_9_.9H_2_O) as a Cr doping precursor was systematically adopted to enhance electrocatalytic properties and performance. The HER/OER and 2-E OWS measurements were conducted in potassium hydroxide (KOH), sulfuric acid (H_2_SO_4_) and phosphate-buffered saline (PBS) electrolytes. The different pH ranges are adapted as 1 M KOH (pH ~ 14) alkaline, 0.5 M H_2_SO_4_ (pH ~ 0) acidic and 1 M PBS (pH~ 7.4) neutral water. All chemicals were analytical grades purchased from Sigma-Aldrich and used without further treatment. Deionized water (DI) was used throughout the experiments. For natural water applications, actual seawater (SW) and river water (RW) were adapted. SW and RW were collected from the Yellow Sea in Inchon and the Han River in Seoul, South Korea, which were used after simple filtration.

**S-1.5. Fabrication of HER and OER benchmark electrodes**

Pt/C (HER) and RuO_2_ (OER) benchmark electrodes were fabricated on nickel foam (NF) substrate ^[24]^. For the synthesis of Pt/C, 20 mg of Pt/C and 60 μL of 5% Nafion (117 solutions, Sigma-Aldrich) were combined in a mixture of both 5 ml ethanol and DI water (50:50). The prepared mixed solution was ultrasonically treated for 30 min to obtain a clear mixture. The Pt/C was deposited on bare NF by immersing it in a mixed solution for 30 min ^[29]^. Further, the Pt/C electrode was dried in the ambient. The morphological, elemental and electrochemical analysis of the Pt/C benchmark can be found in Fig. S5. The Pt/C HER benchmark demonstrated 91 and 235 mV overpotentials at 100 and 600 mA/cm^2^ current density in 1 M KOH as shown in Figs. S5(c) – S5(d). The RuO_2_ electrode was synthesized using the same fabrication process by dispersing 40 mg of RuO_2_ and 60 μL of 5 Wt % Nafion (117 solutions, Aldrich) in 50:50 ethanol and DI water (5 ml of both) ^[29]^. Further, the mixed solution was ultrasonically treated for 30 min. Further, the bare Ni foam was immersed in a mixed solution for 30 min and was dried in the ambience. The morphological, elemental and electrochemical analysis of the RuO_2_ benchmark can be found in Fig. S6. The RuO_2_ demonstrated 310 and 650 mV overpotential at 100 and 600 mA/cm^2^ current density in 1 M KOH as shown in Figs. S6(c) – S6(d). A RuO_2_(+) ǁ Pt/C (-) system was used as the benchmark to compare with Cr/NiBP bifunctional and hybrid systems under various operational conditions.

**HER and OER TOF of Pt/C and RuO_2_:** Turnover frequency (TOF) is used to indicate the intrinsic activity parameters, which can measure the number of reactant molecules converted to product molecules per active site per unit time ^[30]^. The TOF values of Pt/C and RuO_2_ can be obtained based on the following equation ^[31,32]^:

**Active sites and HER TOF values:**

$=\frac{EDS \%}{100} \times amount of loading per area\times Molecular mass\times\left( \frac{Avogadro Number}{Per concentration (mmol)} \right)$

$$=\frac{53.83}{100} \times0.4\frac{\mathrm{mg}}{\mathrm{cm}^{2}}\times\frac{1 mmol}{195.078 mg}\times\left( \frac{6.022 \times{10}^{23}\mathrm{mmol}}{1 mmol} \right)$$

${=6.64\times10}^{17} Active sites.{cm}^{-2}$ per mole

The molecular mass of Pt is 195.078 mg/mol, where 1 AMU is 1 g/mol. The average weight percentage (% ) of Pt (53.83 %) was determined from the EDS quantitative analysis in Fig. S5(b). Assuming that all Pt atoms are catalytically active for redox reaction. The loading amount per area of the Pt/C benchmark was 0.4 mg/cm^2^  by weighing the bare NF (0.0432) and sample after the synthesis (Pt/C: 0.0436 gm).

In the HER LSV curve, the Pt/C exhibited 290.75 mA/cm^2^ current density at 176 mV fixed overpotential in 1 M KOH as seen in Fig. 5(c).

**Pt/C TOF values =** $\frac{3.12 \times{10}^{15}\times290.75}{{6.64\times10}^{17}}$

= 1.36 site^-1^ s^-1^

**Active sites and OER TOF values:**

$=\frac{EDS \%}{100} \times amount of loading per area\times Molecular mass\times\left( \frac{Avogadro Number}{Per concentration (mmol)} \right)$

$$=\frac{61.63}{100} \times0.4\frac{\mathrm{mg}}{\mathrm{cm}^{2}}\times\frac{1 mmol}{101.07 mg}\times\left( \frac{6.022 \times{10}^{23}\mathrm{mmol}}{1 mmol} \right)$$

${=1.46\times10}^{18} Active sites.{cm}^{-2}$ per mole

The molecular mass of Ru is 101.07 mg/mol, where 1 AMU is 1 g/mol. The average weight percentage (%) of Ru (55.1 %) was determined from the EDS spectra in Fig. S6(b). Assuming that all Ru atoms are catalytically active for redox reaction. The loading amount per area was 0.4 mg/cm^2^  by weighing the bare NF (0.0432) and sample after the synthesis (RuO_2_: 0.0436 gm).

In OER LSV curves, the RuO_2_ demonstrated 113.86 mA/cm^2^ current density at 300 mV fixed overpotential in 1 M KOH as seen in Fig. 6(c).

**RuO_2_ TOF values** = $\frac{1.56 \times{10}^{15}\times113.86}{{=1.46\times10}^{18}}$

= 0.1216 site^-1^ s^-1^

The Pt/C and RuO_2_ benchmark electrodes demonstrated 1.36 and 0.1216 site^-1^ s^-1^ TOF values. A more detailed TOF calculation can be found in supplementary text S-1.11.

**S-1.6. NiBP micro sphere (MS) base electrode characterizations**

The NiBP electrode was served as a doping template in this work. The NiBP MS demonstrated vertically grown spherical cluster morphology on NF substrate as shown in Figs. S7(a) – S7(a-1). The interconnected spherical particles of NiBP can work as conductive networks. The EDS measurement has confirmed the presence of Ni, B and P with qualitative analysis in Fig. S7(b). Further, the Raman and XRD analysis was conducted on the NiBP electrode as shown in Fig. S8. The Raman analysis demonstrated characteristic peaks at 465, 705, 943 and 1036 cm^-1^ as seen in Fig. S8(a). The sharp Raman peaks demonstrated good crystal quality. Further, the XRD pattern of NiBP exhibited multiple diffraction peaks as shown in Fig. S8(b) – S8(b-1). The intense peaks around ~ 43^o^ and 52^o^ can belong to (111) and (200) planes of Ni foam (NF) ^[33]^. The bare NiBP demonstrated diffraction peaks ~ 21.62, 31.18, 32.22, 33.08, 35.94, 37.14, 55.02, 58.57, 59.52 and 66.81^o^ as seen in Fig. S8(b). The zoom view showed 36.46, 37.50, 41.81, 42.87, 43.70, 46.77, 47.43, 48.26, 50.75 and 52.87 ^o^  as presented in Fig. S8(b-1). The XRD references or JCPDS card was not found in the literature or database for NiBP. However, the randomly oriented multiple diffraction peaks indicated a polycrystal nature of the NiBP template ^[1,2]^. Previous studies have shown that polycrystalline surfaces can outperform single crystals in HER and OER, offering more active sites, larger electrochemical surface area and improved intrinsic properties ^[2,34]^. The HER and OER electrochemical performance of NiBP are shown in Figs. S9(a) – S9(b). The NiBP demonstrated overpotential values of 327 and 510 mV at 300 mA/cm^2^ for HER and OER in 1 M KOH. Further, the electrochemical impedance spectroscopy (EIS) measurement was conducted on NiBP as shown in Figs. S9(c) – S9(d). The NiBP showed charge transfer resistance (R_ct_) values of 32.30 and 28.90 Ω for HER and OER, indicating low resistance and favorable conductivity ^[35]^. Further, the HER/OER double-layer capacitance (C_dl_) values are evaluated from cyclic voltammetry (CV) curves as seen in Figs. S10 – S11. The NiBP demonstrated HER/OER C_dl_ values of 5.60 and 8.36 mF/cm^2^ as shown in Figs. S11(a) – S11(b), indicating efficient electrochemical surface area (ECSA) ^[36]^. In addition, the ECSA values are calculated from C_dl_ values as shown in Figs. S11(c) – S11(d). The NiBP showed ECSA values of 35 and 52.25 cm^2^ for HER and OER, indicating high intrinsic activity ^[35]^. In summary, the favorable physical and electrochemical properties of NiBP make it a promising template for doping.

**S-1.7.** **Raman analysis of Cr/NiBP**

Figure S30 shows the Raman analysis on Cr/NiBP and bare NiBP electrocatalysts. Generally, Raman spectroscopy is an analytical technique to determine crystal quality and crystallinity by analyzing molecular vibration and interaction. After Cr incorporation, the Raman spectra of Cr/NiBP maintained initial peaks as seen in Figs. S30(a). The characteristic peaks observed at 465, 705, 943 and 1036 cm^‑1^. Previous studies have also shown that post-doped electrodes retain their Raman peak ^[37–39]^. In addition, the NiBP electrode was initially fabricated using a first-step hydrothermal reaction. Further, Cr-doping was introduced into the NiBP matrix via an optimized 2^nd^ step hydrothermal process. Post-annealing can minimize defects and disordered atomic arrangements within the crystal lattice through inter-atomic diffusions ^[40]^. Hydrothermally grown samples may exhibit various defects and atomic dislocations in the crystal lattice ^[40]^. Optimized annealing can reduce various defects and redistribution of atomic arrangements ^[40]^. The Raman’s peak intensity of Cr/NiBP was largely increased as compared to bare NiBP, indicating improved crystallinity. The higher crystal quality can help to enhance the conductivity and ion mobility ^[41,42]^. In previous works, the binary composition Ni-B electrode exhibited intense peaks at 168, 195, 463 and 809 cm^-1 [43]^. The Ni-P catalyst showed intense peaks around 1338 and 1580 cm^-1 [44]^. In addition, the single crystal nickel oxide (Ni-O) electrode showed different peaks at 670, 720, 890, 1110 and 1522 cm^-1^ with (100), (110) and (111) different crystal phases ^[45]^. Further, the Raman vibration mode of the nickel hydroxide phase (Ni(OH)_2_ was found at ~ 580 cm^-1^ [ 36]. The O-Ni-O and Ni-O-H bending vibrations were found at 654 and 465 cm^−1^ for Ni-based Ni/Ni(NiOH)_2_ electrocatalyst ^[47]^. The phosphate (- PO_4_) vibrational characteristics were found at 560 and 945 cm^−1^ in the literature ^[48]^. The peak overserved at 1036 cm^−1^ might correspond to active oxygen (O-O) bonds, particularly those involving metal oxides or oxygen-containing compounds ^[30]^. Overall, The Raman spectra of similar materials exhibited distinct responses. Raman references or JCPDS cards were not available for Cr/NiBP and NiBP in the literature and database. Consequently, a unique Raman signal can indicate the successful formation of the Cr/NiBP electrocatalyst.

The before/after stability Raman spectra comparison of the Cr/NiBP MS electrode is presented in Fig. S58. The stability test was conducted at 1,000 mA/cm^2^ in 1 M KOH for 30 hours duration. The post-stability Raman spectra maintained the initial characteristics peak. Additionally, a new peak emerged at approximately 570 cm⁻¹ as depicted in Figure S58(a). Additional peak formation can be attributed to high valance state transformation and nickel oxyhydroxide (NiOOH) species formation ^[46]^. Generally, the Ni^+2^ species can transfer to higher valance species of Ni^3+^ (NiOOH) during the redox process ^[37,49]^. However, the overall intensity of the Raman peaks decreased, likely due to surface oxidation and the adsorption of metal oxide (M-O) and metal hydroxide (M-OH) species ^[50]^. The NiOOH formation was observed in the OER CV (Fig. S36). Additionally, post-stability XPS analysis confirmed surface oxidation.

**S-1.8. X-ray diffraction (XRD) analysis of Cr/NiBP**

The X-ray diffraction (XRD) pattern of Cr/NiBP and bare NiBP are shown in Fig. S31. Generally, XRD is a useful technique for determining a material's crystal structure, crystalline phases and crystallinity. The Cr/NiBP MS and bare NiBP electrocatalyst exhibited the same diffraction peaks as shown in Figs. S31(a) – S31(b), indicating that Cr incorporation did not alter the initial NiBP phase structure. The full scan XPS demonstrated 31.18, 32.22, 33.08, 35.94, 37.14, 55.02, 58.57, 59.52 and 66.8^o^. The zoom-in-view showed 36.06, 37.07, 39.59, 42.37, 46.87, 47.67, 50.02, 53.77 and 54.84^o^ in Figs. S31(a-1) – S31(b-1). The observed intense diffraction peaks at ~ 43^o^ and 52^o^ can be attributed to (111) and (200) planes of Ni foam (NF) substrate ^[33]^. Recent studies have also suggested that polycrystal structures are advantageous for achieving favorable electrocatalytic properties and enhanced stability ^[51–53]^. However, the XRD diffraction peak intensity was increased along with combined doping and annealing, suggesting improved crystal quality and crystallinity. Improved crystallinity can lead to higher conductivity and faster redox reactions ^[54]^. After Cr-doping, XRD analysis revealed no additional phases, indicating the preservation of the host lattice structure and the successful incorporation of Cr nanoclusters ^[55,56]^. The absence of new phases was likely due to the low Cr content, as easily found by EDS analysis ^[56]^. It is also suggested that Cr atoms were dispersed or integrated into very small clusters, below the detection of XRD ^[57,58]^. Furthermore, the presence of Cr was confirmed by XPS analysis, which is consistent with previous studies ^[14,56]^. As previously discussed, post-annealing treatment can enhance crystal quality and crystallinity through atomic diffusion ^[17,59]^. The Cr/NiBP MS showed randomly oriented tinny diffraction peaks throughout the 2θ range, indicating the polycrystal structure. Also, a recent study suggested that polycrystal can perform better than single crystal due to unique more active sites and enlarged electrochemical surface area, which can contribute to rapid charge transfer, higher electrochemical kinetics and stability ^[60–62]^.

Figure S39 shows the XRD pattern analysis of the Cr/NiBP MS catalyst with the PDF cards of Ni_3_B, NiB, NiP and NiP_3_. In addition, the Cr/NiBP and NiBP compositions are not available in the international crystal structure database and literature. The Ni_3_B (ICSD ID: 9040), NiB (ICSD ID: 10730), NiP (ICSD ID: 10892) and NiP_3_(ICSD ID: 8624) crystal phases are shown in Figs. S39(c) – S39(f). All closely related materials exhibited distinct diffraction patterns as compared to the Cr/NiBP MS electrocatalyst, and their overall patterns did not match. Therefore, the unique XRD pattern could suggest the formation of a polycrystalline Cr/NiBP electrocatalyst.

**S-1.9. X-ray photoelectron spectroscopy (XPS) of Cr/NiBP**

X-ray photoelectron spectroscopy (XPS) was conducted on Cr/NiBP as illustrated in Figs. 2(e) – 2(f-3). The full-scan XPS survey spectrum demonstrated the co-existence of Cr, Ni, B and P elements as shown in Fig. 2(e). The observed C 1s and O 1s peaks can be attributed to binding energy (BE) calibration and exposure to air. The high-resolution XPS spectra of Cr 2p, Ni 2p, B 1s and P 2p species are presented in Figs. 2(f) – 2(f-3). In the Cr 2p spectrum, the Cr 2p_3/2_ and Cr 2p_1/2_ peaks were observed at 574.9 and 584 eV BEs as shown in Fig. 2(f). The corresponding Cr elemental peaks can be found at 574.4 and 583.6 eV in the standard XPS handbook ^[63]^. For comparison, the elemental peak positions of all elements are considered from the standard XPS handbook ^[63]^. The binding energies (BEs) of Cr 2p_3/2_ and Cr 2p_1/2_ peaks were shifted by 0.5 and 0.4 eV positively from pristine, suggesting increased electron density around the NiBP matrix. The binding energy of Cr 2p_3/2_ and Cr 2p_1/2_ can be attributed to the higher valance state of Cr^3+^, which is consistent with previous work ^[14,16,64]^. Trivalent Cr metallic species can also exist as Cr_2_O_3_ (chromium(III) oxide) and Cr(OH)_3_ (chromium(III) hydroxide) in low concentrations during partial oxidation ^[65]^. In the previous study, the Cr-doped Cu/CoO electrode showed Cr 2p_3/2_ and Cr 2p_1/2_ intense peaks at 576.6 and 586.4 eV with positive BE ^[16]^. The presence of high-valance state Cr can facilitate faster reaction kinetics and water dissociation ^[16,66]^. Here, the Cr atoms were incorporated into the NiBP matrix with a minor BE shift ^[67]^. The minor BE shift might result from changes in the charge distribution, which is caused by the lattice strain/compressive behaviors within the crystal ^[68]^. The TEM analysis in Figs. 2(b) – 2(b-2) showed nanocluster incorporation of Cr separately around the NiBP matrix. The nanoclusters can be composed of Cr atoms with zero valance state. The zero-valent Cr and Cr 2p_3/2_ were found at 574.2 and 574.4 eV in the database, which may overlap due to remarkably close BE in XPS spectra.

The Ni 2p spectrum demonstrated Ni 2p_1/2_, Ni 2p_3/2_ and Ni(OH)_2_ peaks respectively at 873.1, 852.8 and 856.2 eV BEs as shown in Fig. 2(f-1). The pristine Ni 2p_1/2_and Ni 2p_3/2_ peaks can be found at 869.97 and 852.7 eV. The Ni 2p_1/2_and Ni 2p_3/2_ peaks were shifted positively by 3.1 and 0.2 eV, indicating electron donation. In the previous study, the Ni 2p_1/2_and Ni 2p_3/2_ characteristics peaks were found at 874.5 and 853.1 eV respectively for the Cr/NiSe-N catalyst ^[57]^. Further, the Ni(OH)_2_ peak was observed at 856.2 eV from adsorption of OH^-^ ions. The Ni(OH)_2_ and Ni-OOH species were found at 855.6 and 855.8 eV respectively, with very similar BEs in the database ^[69]^. The Ni^2+^ state can be oxidized into the higher valance state of Ni^3+ [70]^. The Ni satellite peaks appeared at 879.8 and 861.8 eV ^[71]^. In the study, the satellite peaks were found at 879.8 and 861.1 eV for quasi-amorphous metallic nickel (QAMN) catalyst ^[72]^. In the B spectrum, the B 1s and trivalent boron oxide (B_2_O_3_) peaks were observed at 188.1 and 192.6 eV as seen in Fig. 2(f-2). The B 1s state can be found at 187.3 eV in the database. In the BE comparison, the B 1s peak was shifted positively by 0.8 eV, implying the donations of electrons. The Cu-Co-B catalyst exhibited B 1s and B_2_O_3_ peaks at 188.2 and 191.7 eV in the previous work ^[73]^. In the P 2p spectrum, the P 2p_3/2_, P 2p_1/2_and P-O oxide states were observed at 128.78, 129.60 and 133.3 eV respectively as shown in Fig. 2(f-3). The elemental peaks of 2p_3/2_and P 2p_1/2_were found at 129.90 and 130.74 eV. In comparison, the 2p_3/2_and P 2p_1/2_ peaks were shifted negatively by 1.12 and 1.14 eV, indicating the acceptance of electrons. The Fe-P/CoNi-S catalyst showed 2p_3/2_and P 2p_1/2_ states at 129.6 and 131.4 eV ^[74]^. The P-O species was observed at 133.3 eV ^[74]^. Phosphorus oxide species PO_4_ and PO_3_ can be found at 133.2 and 134.6 eV, which are close to BE differences. The P-O peak was found at 134.3 eV for Fe-P/CoNi-S catalyst in the literature ^[74]^. Overall, the Ni and B elements supplied electrons to P for electronic interaction and NiBP formation. Then, the Cr was incorporated into the NiBP matrix as a nanocrystal phase.

**S-1.10. Cyclic CV in the OER range of Cr/NiBP**

Figure S36 shows the OER cyclic voltammetry (CV) scan profile of the Cr/NiBP (30 min-annealed) electrode in 1 M KOH. The Cr/NiBP electrocatalyst showed minor oxidation around the turnover region in the OER LSV curve due to the high valance state transformation of metallic species in Fig. 4(f). The OER reaction pathways are described with (i), (ii) and (iii) steps as illustrated in Fig. S36(a). The anodic and cathodic redox peaks were identified as A_p_ and C_p_, respectively. The CV profile demonstrated both oxidative and reductive cycles in zoom-in-view as shown in Fig. S36(a-1). In the first step reaction, the initial potential region (i) can be described as M + OH^-^  → M-OH _ad_, where the metallic site (M) begins to adsorb hydroxide ions to form M-OH _ad_ species ^[75]^. In the middle portion of step (ii), the M-OH _ad_ intermediate again adsorbed ^-^OH ions as M-OH _ad_ + OH^-^  → M-OOH _ad_ + e- ^[75]^. In step (iii), the O_2_ finally was generated and released as OH^-^  + M-OOH _ad_ → M+ O_2_ + H_2_O ^[76]^. The formation of oxyhydroxide (M-OOH) species indicates a high-valence state transformation of metallic species. Specifically, nickel species can transform from Ni(OH)_2_ (Ni^2+^) to NiOOH (Ni^3+^) ^[76]^. The NiOOH phase may facilitate the rate-limiting step in the OER process and effectively reduce overpotential, accelerating O-O bond formation ^[75]^. The anodic oxidation peak (A_p_) was observed at ~ 1.43 V and the cathodic reduction peak (C_p_) appeared at ~ 1.33 V. Further, the NiOOH phase formation was also confirmed by post-stability analysis. It indicated that Cr/NiBP undergoes surface reconstruction during OER operations, which leads to a reduction in overpotential and improved structural stability ^[77,78]^. The NiOOH phase formation may not only enhance performance but also act as a protective oxide layer of active species, improving stability ^[77,79]^. Furthermore, the high-valence Cr species (Cr^4+^/Cr^6+^) can facilitate the oxidation process and reduce the free energy of oxygen-containing intermediates ^[80]^. It can also stabilize active sites at a high valance state ^[81]^. Previous studies also suggested that the Cr atoms can influence the adsorption energies of O* and *OH intermediates, which is advantageous for OER process ^[82]^.

**S-1.11. TOF calculation of Cr/NiBP MS**

Turnover frequency (TOF) is defined as the number of converted target products (H_2_ or O_2_) generated per site per unit of time ^[83]^. The HER/OER TOF calculation of Cr/NiBP electrocatalysts can be obtained as shown in eq. (4) ^[31,84]^:

**General TOF:**

TOF **=** $\frac{\frac{Total number of H_{2}\mathrm{or}O_{2} \mathrm{turnover}}{Geometric area {(cm}^{2})} \times Current density}{\frac{Number of active sites}{Geometric area {(cm}^{2})}}$ (4)

**Total H_2_ turnover:**

= $\left( j\frac{\mathrm{mA}}{\mathrm{cm}^{2}} \right)\left( \frac{1\frac{C}{s}}{1000 mA} \right)\left( \frac{1 \frac{\mathrm{mol}}{e}}{96485 C} \right)\left( \frac{1 mol H_{2}}{2 \frac{\mathrm{mol}}{e}} \right)\left( \frac{6.022 \times{10}^{23}\mathrm{molecules}H_{2}}{1 mol H_{2}} \right)$

= $3.12 \times{10}^{15} \left( \frac{\frac{H_{2}}{s}}{\mathrm{cm}^{2}} \right) per (\frac{\mathrm{mA}}{\mathrm{cm}^{2}})$

**Total O_2_ turnover:**

= $\left( j\frac{\mathrm{mA}}{\mathrm{cm}^{2}} \right)\left( \frac{1\frac{C}{s}}{1000 mA} \right)\left( \frac{1 \frac{\mathrm{mol}}{e}}{96485 C} \right)\left( \frac{1 mol O_{2}}{4 \frac{\mathrm{mol}}{e}} \right)\left( \frac{6.022 \times{10}^{23}\mathrm{molecules}O_{2}}{1 mol O_{2}} \right)$

= $1.56 \times{10}^{15} \left( \frac{\frac{O_{2}}{s}}{\mathrm{cm}^{2}} \right) per (\frac{\mathrm{mA}}{\mathrm{cm}^{2}})$

**Active sites of Cr/ NiBP:**

$=\frac{EDS \%}{100} \times amonut of loading per area\times Molecular mass\times\left( \frac{Avogadro Number}{Per concentration (mmol)} \right)$ (5)

$=\frac{7.80}{100} \times0.20\frac{\mathrm{mg}}{\mathrm{cm}^{2}}\times\frac{1 mmol}{51.99 mg} \times\frac{57.96}{100} \times0.20\frac{\mathrm{mg}}{\mathrm{cm}^{2}}\times\frac{1 mmol}{58.69 mg}\times\left( \frac{6.022 \times{10}^{23}\mathrm{mmol}}{2 mmol} \right)$

${=1.78\times10}^{17} Active sites.{cm}^{-2}$ per mole

The active site of the Cr/NiBP catalyst can be calculated using eq. (5). The average weight percentages of metallic Cr and Ni were determined to be 7.80 and 57.96 (wt. %), respectively, from the EDS analysis of Cr/NiBP electrocatalysts in Figs. S25(a) – S25(d). The average loading material of Cr/NiBP was 0.20 mg/cm^2^ (bare Ni substrate 0.0436 gm and after sample preparation 0.04340 gm). The molecular mass of Cr and Ni are 51.99 and 58.69 mg/mmol, where 1 AMU is 1 gm/mol.

**HER TOF values:**

HER TOF of best Cr/NiBP (30 m) **=** $\frac{3.12 \times{10}^{15}\times300}{{1.78\times10}^{17}}$

= 5.25 site^-1^ s^-1^

**OER TOF values:**

OER TOF of best Cr/NiBP (30 m) **=** $\frac{1.56 \times{10}^{15}\times300}{{1.78\times10}^{17}}$

= 2.62 site^-1^ s^-1^

The HER/OER TOF of Cr/NiBP (30 m) electrocatalyst can be calculated as above. The annealed Cr/NiBP electrodes identified as 15 m, 30 m, 60 m and 80 m demonstrated 89.9/265.96, 100/300, 81.32/234.12 and 65.38/208.34 mA/cm^2^ HER current density at 78/174 mV fixed overpotential in 1 M KOH. Similarly, the 15 m, 30 m, 60 m, and 120 m annealed Cr/NiBP electrodes demonstrated 81.41/244.29, 100/300, 67.28/181.25 and 36.76/136.07 mA/cm^2^ OER current density 250/300 mV at fixed overpotential in 1 M KOH. The HER/OER TOF values of the corresponding Cr/NiBP post-annealing duration variation electrodes are summarized in Figs. 3(e) and 3(j). The 30 m annealed Cr/NiBP demonstrated the highest HER/OER TOF values of 5.25 and 2.62 site^-1^ s^-1^ at 174 and 300 mV fixed overpotential.

**Different pH TOF values:**

The different pH TOF calculations of Cr/NiBP MS were conducted at high current density following the same process based on LSV curves in Figs. 4(d) – 4(i). The Cr/NiBP demonstrated 600, 577.25 and 537.10 mA/cm^2^ HER current density at 291 mV fixed overpotential in 1 M KOH, 0.5 M H_2_SO_4_ and 1 M PBS. Similarly, the Cr/NiBP demonstrated 600, 220.21 and 110.21 mA/cm^2^ OER current density at 330 mV fixed overpotential in 1 M KOH, 0.5 M H_2_SO_4_ and 1 M PBS. The calculated different pH TOF values of Cr/NiBP are provided in Fig. S40.

**TOF values of bare NiBP:** The average weight percentages of metallic Ni were determined to be 57.96 (wt. %) from NiBP particles (without substrate) EDS elemental analysis and 58.69 mg/mmol molecular mass, where 1 AMU is 1 gm/mol. The average loading amount of NiBP was 0.18 mg/cm^2^.

Metallic active sites

$=\frac{EDS \%}{100} \times amonut of loading per area\times Molecular mass\times\left( \frac{Avogadro Number}{Per concentration (mmol)} \right)$

$=\frac{58.84}{100} \times0.18\frac{\mathrm{mg}}{\mathrm{cm}^{2}}\times\frac{1 mmol}{58.69 mg}\times\left( \frac{6.022 \times{10}^{23}\mathrm{mmol}}{1 mmol} \right)$

${=1.08\times10}^{17} Active sites.{cm}^{-2}$ per mole

The NiBP demonstrated 109.64 and 29.16 mA/cm^2^ HER/OER current density at fixed overpotentials of 176 and 300 mV in 1 M KOH. The TOF values of NiBP can be calculated as below:

HER TOF **=** $\frac{3.12 \times{10}^{15}\times109.64}{{1.08\times10}^{17}}$

= 3.16 site^-1^ s^-1^

OER TOF **=** $\frac{1.56 \times{10}^{15}\times29.16}{{1.08\times10}^{17}}$

= 0.42 site^-1^ s^-1^

The bare NiBP exhibited TOF values of 3.16 and 0.42 site^-1^ s^-1^ at 176 and 300 mV overpotential in 1 M KOH. The TOF values were compared with Cr/NiBP under similar conditions in Figs. S34(c) – S34(d).

**S-1.12. Faradaic efficiency calculation of Cr/NiBP MS**

Faradic efficiency (FE) is defined as the ratio between experimentally detected gas products (H_2_/O_2_) and theoretically calculated amounts. The FE of the Cr/NiBP catalyst can be calculated as shown in Eq. (6) ^[85]^:

$$Faradaic efficiecy=\frac{Practically produce mol of H_{2}/ O_{2} gas}{Theoretically Produce mol of H_{2}/ O_{2} gas}\times100 (6)$$

The theoretical value of H_2_ or O_2_ can be calculated by using Faraday’s law as in Eq. (7)

$$n=\frac{I\times t}{z\times F} (7)$$

where n defines the amount of theoretically produced product (H_2_ or O_2_) in mole, I is the current in ampere, t is the time in seconds and z is the number of electrons transferred (for H_2_ (z = 2) and O_2_ (z = 4)) and Faraday constant (F) = 96,485 C mol^-1^. The theoretical values of H_2_ and O_2_ production can be calculated at 300 mA current for 15 ~ 60 minutes duration.

The theoretical value of H_2_ generation: n = 1.39, 2.79, 4.19 and 5.59 mmol respectively during 15, 30, 45 and 60 minutes of observation.

The theoretical value of O_2_ generation: n = 0.699, 1.39, 2.09 and 2.79 mmol respectively during 15, 30, 45 and 60 minutes of observation.

The generated gas was collected using the water-gas displacement method as seen in Fig. S45. The numbers of H_2_ or O_2_  molecules can be calculated using the following ideal gas eq.:

$$PV=nRT (8)$$

where the V (L) is the volume of collected gas. T is the temperature in kelvin, R is the ideal gas constant (0.0821 L.atm.mol-1 K-1) and P is the atmospheric pressure (~1 atm). This unit for R offers a convenient relationship between experimentally measured gas volume (in liters), simplifies calculations and aligns well with common volume units ^[86]^.

**The number of H_2_ in water-gas displacement:**

The volume of H_2_ gas collected was 0.0311, 0.02385, 0.09688 and 0.131 L respectively for 15, 30, 45 and 60 minutes.

For 60 minutes duration,

$$\left( 1 atm \right) \left( 0.131 L \right)=n\left( 0.0821\frac{\mathrm{atm}}{\mathrm{mol}K} \right)\left( 298K \right)$$

$n=$ 5.35 mmol

**The number of O_2_ in water-gas displacement:**

The volume of O_2_ gas collected was 0.0154, 0.313, 0.0474 and 0.0653 L respectively for 15, 30, 45 and 60 minutes.

For 60 minutes duration,

$$\left( 1 atm \right) \left( 0.0653 L \right)=n\left( 0.0821\frac{\mathrm{atm}}{\mathrm{mol}K} \right)\left( 298K \right)$$

$n=$ 2.67 mmol

A similar process was followed to calculate other observation durations following Eq. (8). The theoretical/practical values comparisons were presented in Figs. S46. The summarized HER/OER faradaic efficiency measurement of Cr/NiBP can be found in Fig. 4(c). The Cr/NiBP demonstrated HER/OER faradaic efficiency of 95.88 and 95.69 % in 1 M KOH for 60 min duration. The high FE of Cr/NiBP demonstrated extremely low energy consumption, suggesting its suitability for practical applications.

**S-1.13. Cr doping effect**

The effects of Cr doping on NiBP base electrode were analyzed using various characterizations, including Raman, XRD, EIS, C_dl_ values, ECSA and LSV performance as shown in Figs. S30 – S35. The Raman and XRD analysis of Cr/NiBP and NiBP were illustrated in Figs. S30 and S31. The Cr/NiBP demonstrated improved crystal quality and crystallinity in both analyses. More details can be found in supplementary texts S-1.7 and S-1.8. The electrochemical characterizations before/after Cr doping are shown in Figs. S32 – S35. The electrochemical impedance spectroscopy (EIS) of NiBP and Cr/NiBP was conducted in 1 M KOH as shown in Fig. S32. The Cr/NiBP showed HER/OER low charge transfer resistance (R_ct_) values of 18.55 and 16.27 Ω as compared to bare NiBP ( 32.30 and 28.90 Ω ) in Figs. S32(a) – S32(b), indicating improved conductivity with Cr doping. The comparison of double-layer capacitance (C_dl_) and electrochemical surface area (ECSA) values is shown in Fig. S33. The C_dl_ value of Cr/NiBP significantly increased from 5.60 to 12.5 mF/cm^2^ for HER and from 8.36 to 16.4 mF/cm^2^ for OER as shown in Figs. S33(a) – S33(b), suggesting additional active sites with Cr. The Cr/NiBP MS electrocatalyst demonstrated significantly larger HER and OER ECSA of 78.12 and 102.50 cm^2^ as compared to bare NiBP (35 and 52.25 cm^2^) in Figs. S33(c) – S33(d). Overall, the Cr-doping enlarged ECSA values by 2.2 and 1.96 times for HER and OER, implying a greater number of sites available for the reaction. The Cr/NiBP demonstrated significantly low overpotential and high LSV performance over bare NiBP as specified in Figs. S34(a) – S34(b). The Cr/NiBP showed low overpotentials of 176/300 mV at 300 mA/cm^2^ as compared to bare NiBP values of 327/510 mV for HER/OER in 1 M KOH. After Cr-doping, the HER/OER overpotential was reduced by 151 and 210 mV at 300 mA/cm^2^, suggesting successful Cr incorporation. The superior performance of Cr/NiBP is attributed to improved conductivity, increased active site density and expanded surface area ^[14,87]^. Higher TOF values generally indicate more efficient active sites and faster reaction kinetics. Furthermore, TOF values were compared for NiBP and Cr/NiBP at fixed overpotentials of 176 mV and 300 mV in 1 M KOH as shown in Figs. S34(c) – S34(d). The Cr/NiBP demonstrated superior HER/OER TOF values of 5.25 and 2.62 site^-1^s^-1^ as compared to NiBP (3.16 and 0.42 site^-1^s^-1^), indicating higher H_2_/O_2_ generation rate due to a greater number of catalytically active sites derived from both Cr and Ni. To assess the intrinsic HER and OER activities, the LSV data were normalized by ECSA as shown in Fig. S35. The results indicate that Cr/NiBP exhibits superior activity, attributable to both its large ECSA and higher intrinsic activity. Additionally, the HER/OER performance of Cr/NiBP was compared to bare Ni foam (NF) in Fig. S44. Finally, the bifunctional 2-E overall water splitting (OWS) performance of Cr/NiBP and NiBP was compared in 1 M KOH in Fig. S47. Using both anode and cathode, the Cr/NiBP (+, -) surpassed the NiBP (+, -) system with large potential differences in 1 M KOH as shown in Fig. S47(a). The Cr/NiBP demonstrated low cell voltages of 2.08 and 2.63 V at 1,000 and 2,000 mA/cm^2^ as compared to the bare NiBP values of 2.90 and 3.62 V as summarized in Fig. S47(b).

**S-2. Optimizations of Cr/NiBP micro sphere electrode**

**S-2.1. Substrate and other necessary**

**S-2.1.1. Block diagram of Cr/NiBP fabrication steps**

**
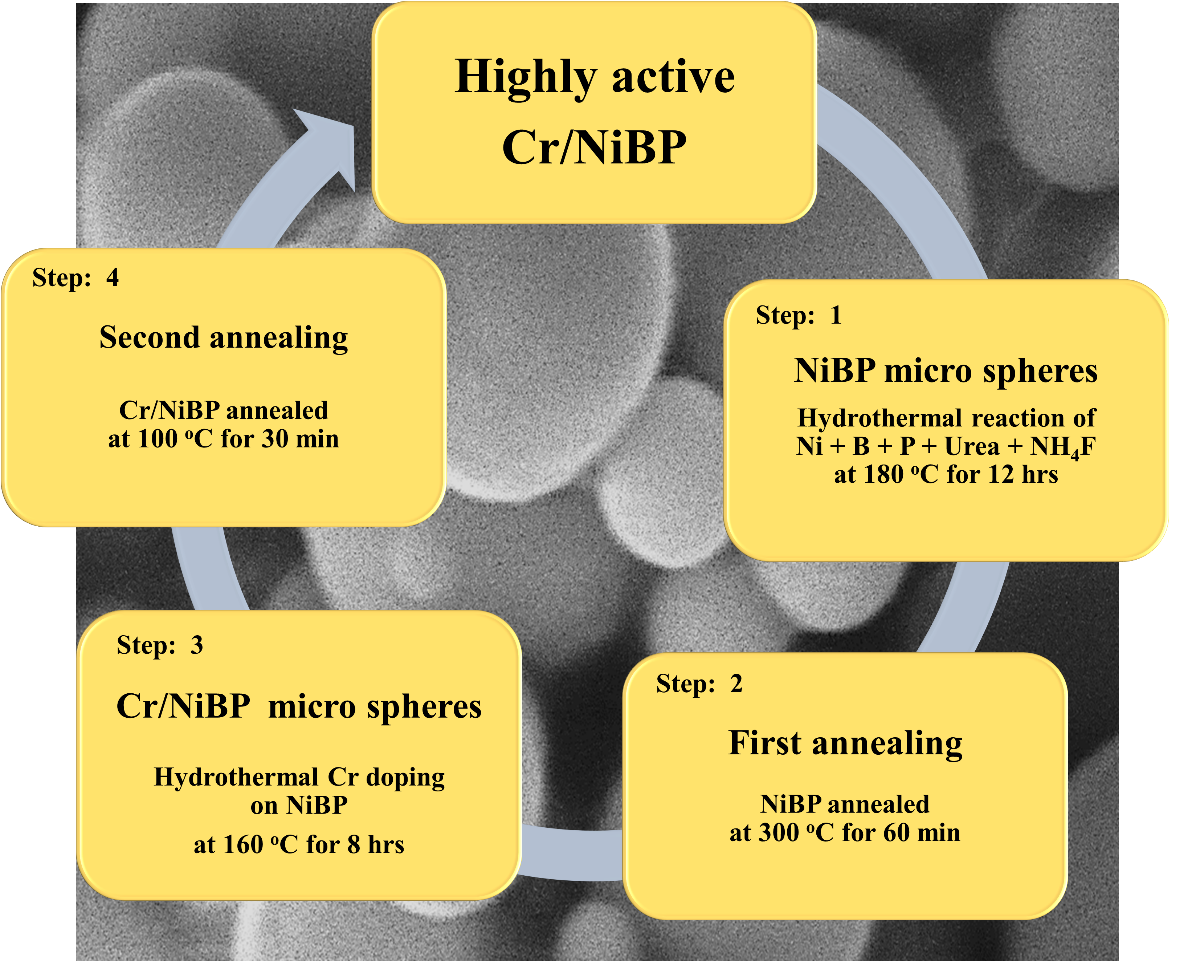
**

**Figure S1.** Schematic illustration of the step-by-step synthesis process of Cr/NiBP micro spherical electrocatalyst. Firstly, NiBP micro sphere (MS) template was synthesized by the hydrothermal reaction at 180 ^o^C for 12 hrs with the precursors of 1 mM Ni, 3.6 mM B**,** 8.4 mM P, 10 mM urea and 10 mM NH_4_F ^[17]^. Further, the Cr-doping was adapted on NiBP MSs through a hydrothermal doping approach with the systematic optimization process in step 3. More details on the Cr/NiBP fabrication process can be found in the supplementary information (SI) text S-1.1. Synthesis of Cr/NiBP electrocatalysts.

**S-2.1.2. Characterization of bare Ni foam (NF)**


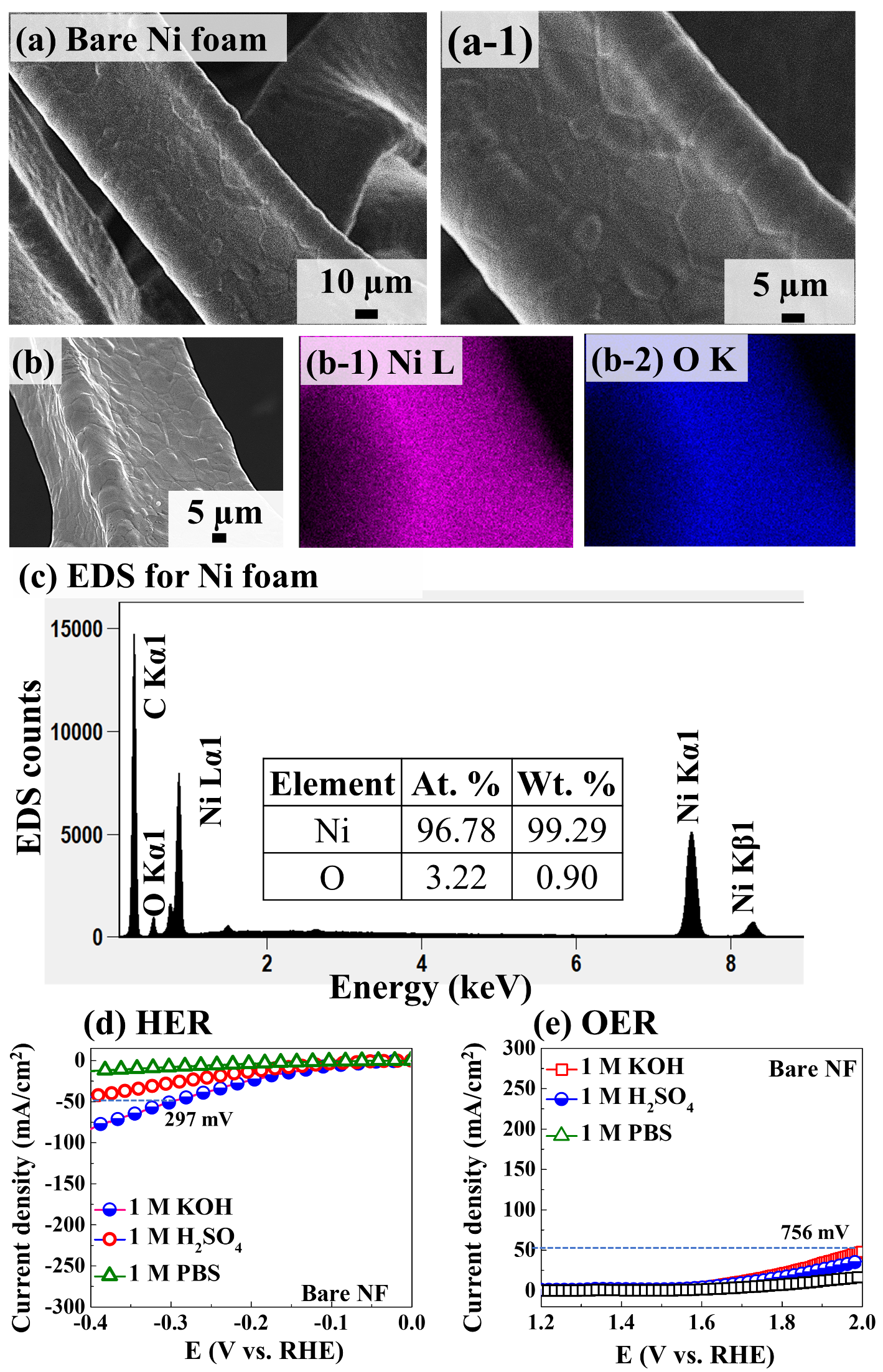


**Figure S2**. Morphological and elemental analysis of 3-D porous bare Ni foam (NF) substrate. (a) & (a-1) SEM images. (b) - (b-2) EDS phase maps. (c) EDS spectrum with corresponding elemental %. (d) & (e) HER and OER performance of bare NF in 1 M KOH, 0.5 M H_2_SO_4_ & 1 M PBS solutions.

**S-2.1.3. Scan rate variations on Cr/NiBP**


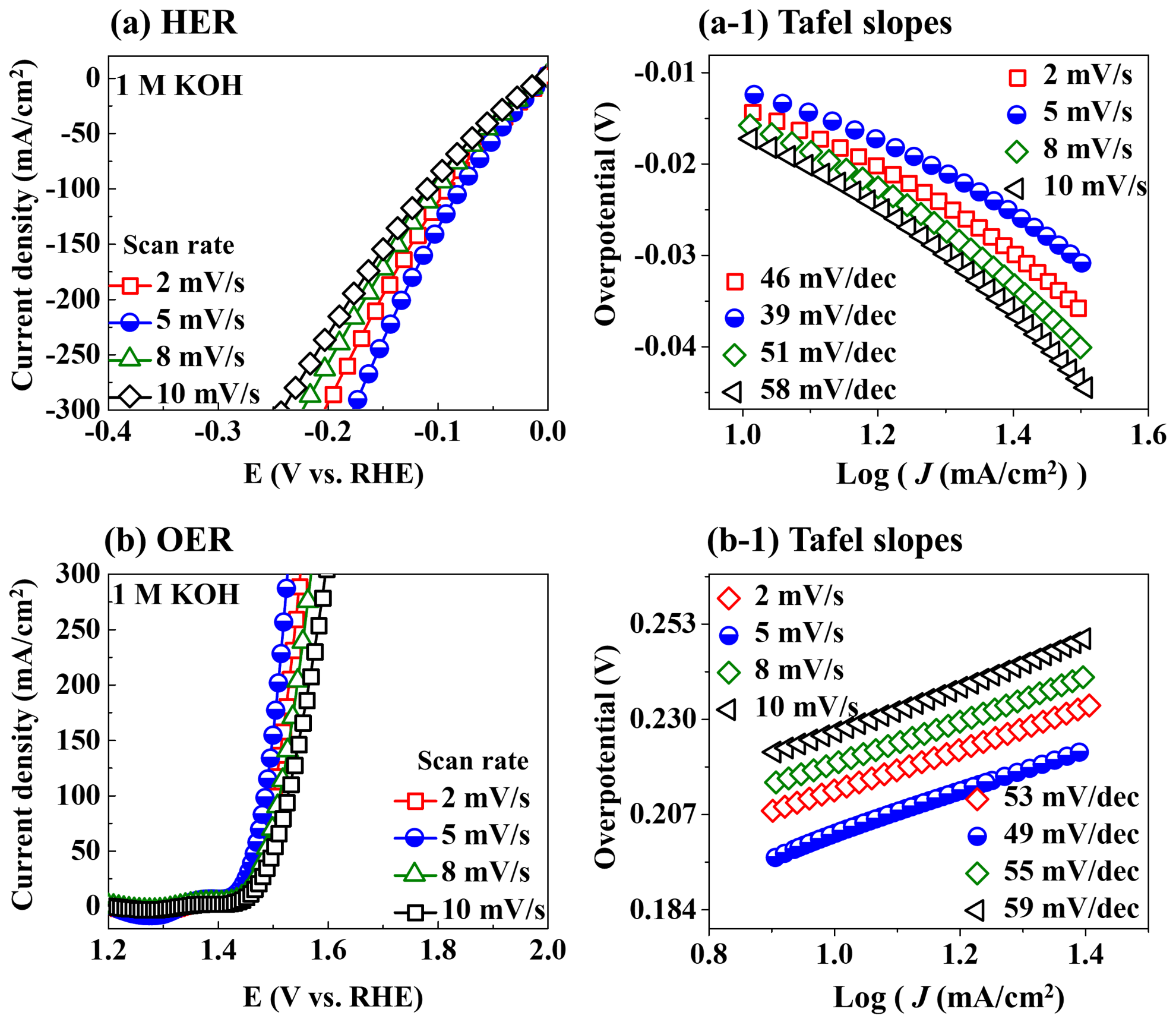


**Figure S3.** LSV scan rates variation in HER/OER activity of Cr/NiBP MSs with corresponding Tafel slopes. (a) & (b) HER/OER LSV curves in different scan rates between 2 ~ 10 mV/s. (a-1) & (b-1) Corresponding Tafel slopes. The obtained overpotential and slope values vary at different scan rates as seen above. More details can be found in SI text S-1.2 Electrochemical Characterizations.

**S-2.1.4. EIS applied voltage variations on Cr/NiBP**


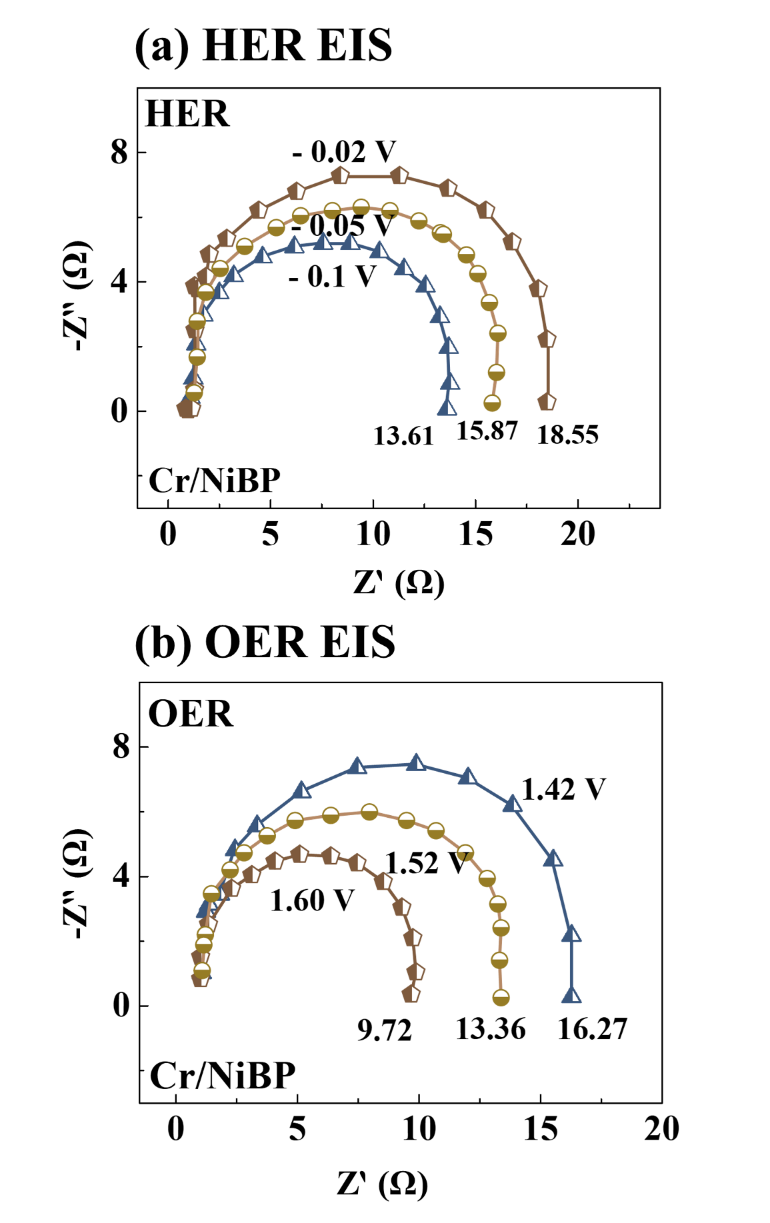


**Figure S4.** (a) & (b) HER and OER electrochemical impedance spectroscopy (EIS) plots with different applied voltages on Cr/NiBP electrocatalyst. The charge transfer resistance (Rct) values varied based on the applied voltages. More detailed descriptions can be found in SI text S-1.2 Electrochemical characterizations.

**S-2.1.5. HER benchmark electrode: Pt/C characterization**


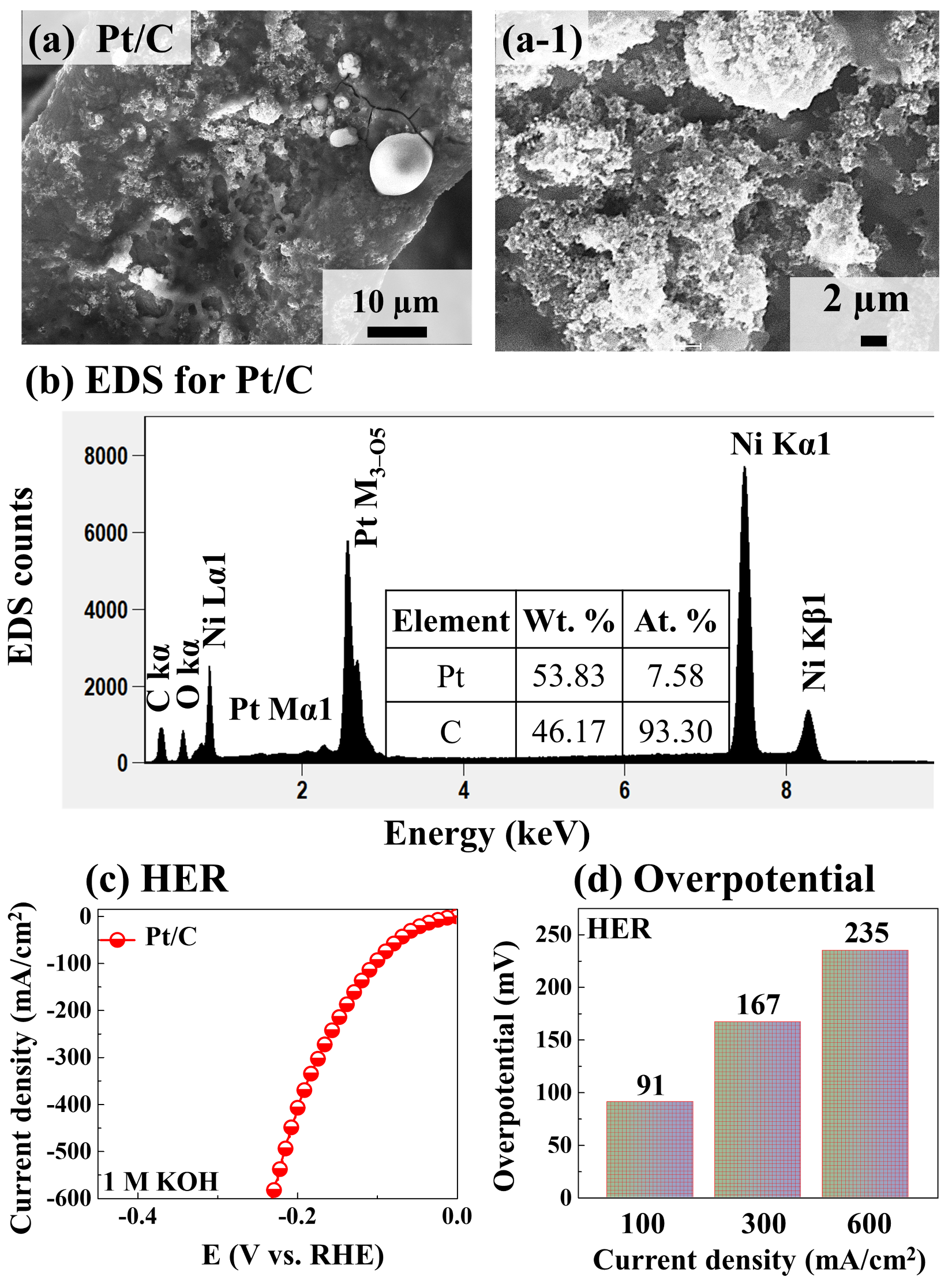


**Figure S5.** (a) & (a-1) SEM images of Pt/C benchmark electrode. (b) EDS spectra with corresponding elemental percentages. (c) & (d) HER LSV performance and corresponding summarized overpotential values at 100, 300 and 600 mA/cm^2^ in 1 M KOH. More detailed descriptions can be found in SI text S-1.5. Fabrication of HER and OER benchmark electrodes.

**S-2.1.6. OER benchmark electrode: RuO_2_ characterization**


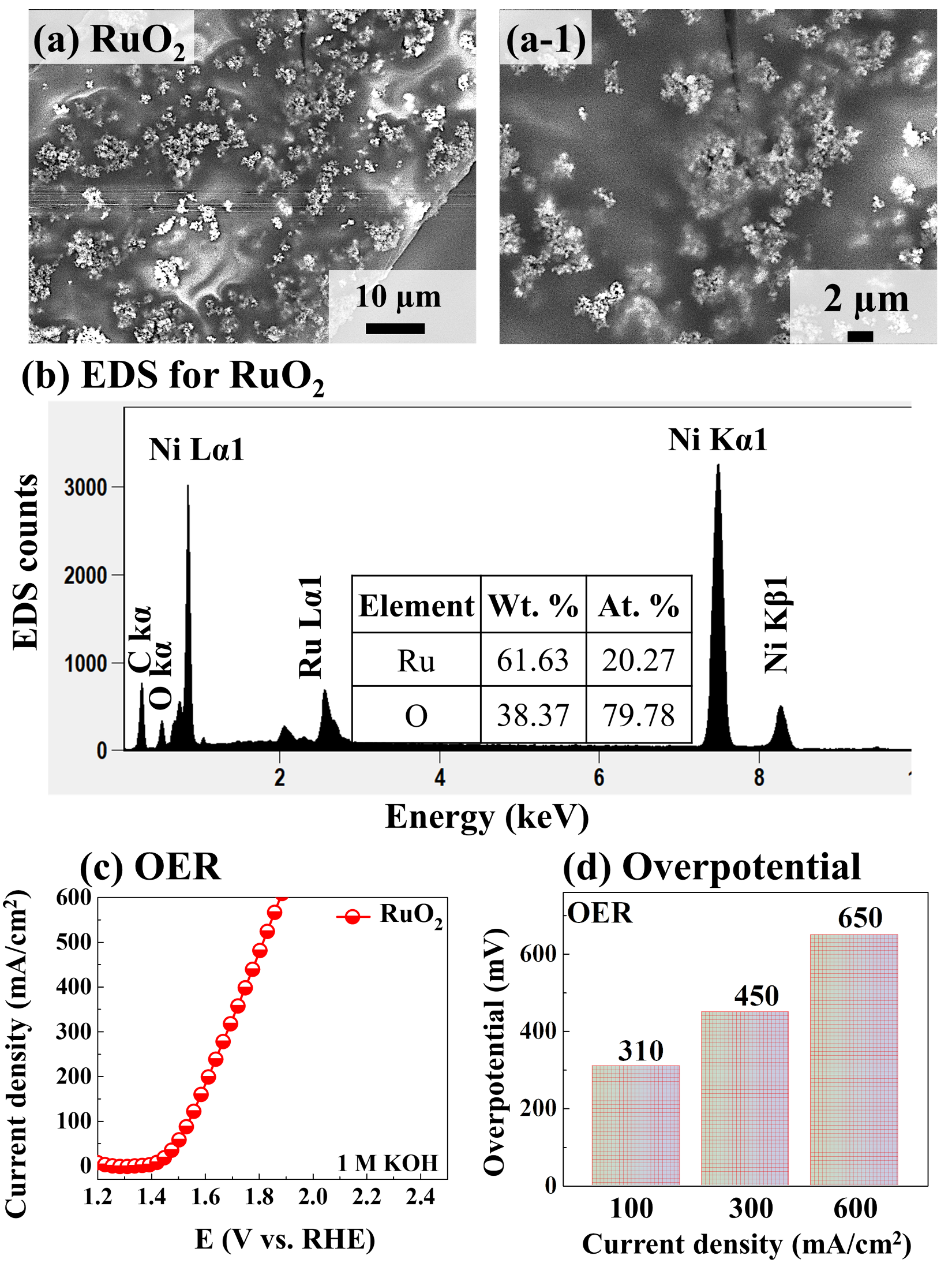


**Figure S6.** (a) & (a-1) SEM images of RuO_2_ benchmark electrode. (b) EDS spectra with corresponding elemental percentages. (c) & (d) OER LSV performance and corresponding summarized overpotential values at 100, 300 and 600 mA/cm^2^ in 1 M KOH. More details can be found in SI text S-1.5. Fabrication of HER and OER benchmark electrodes.

**S-2.2. NiBP base-electrode analysis**

**S-2.2.1. NiBP base-electrode: SEM & EDS**


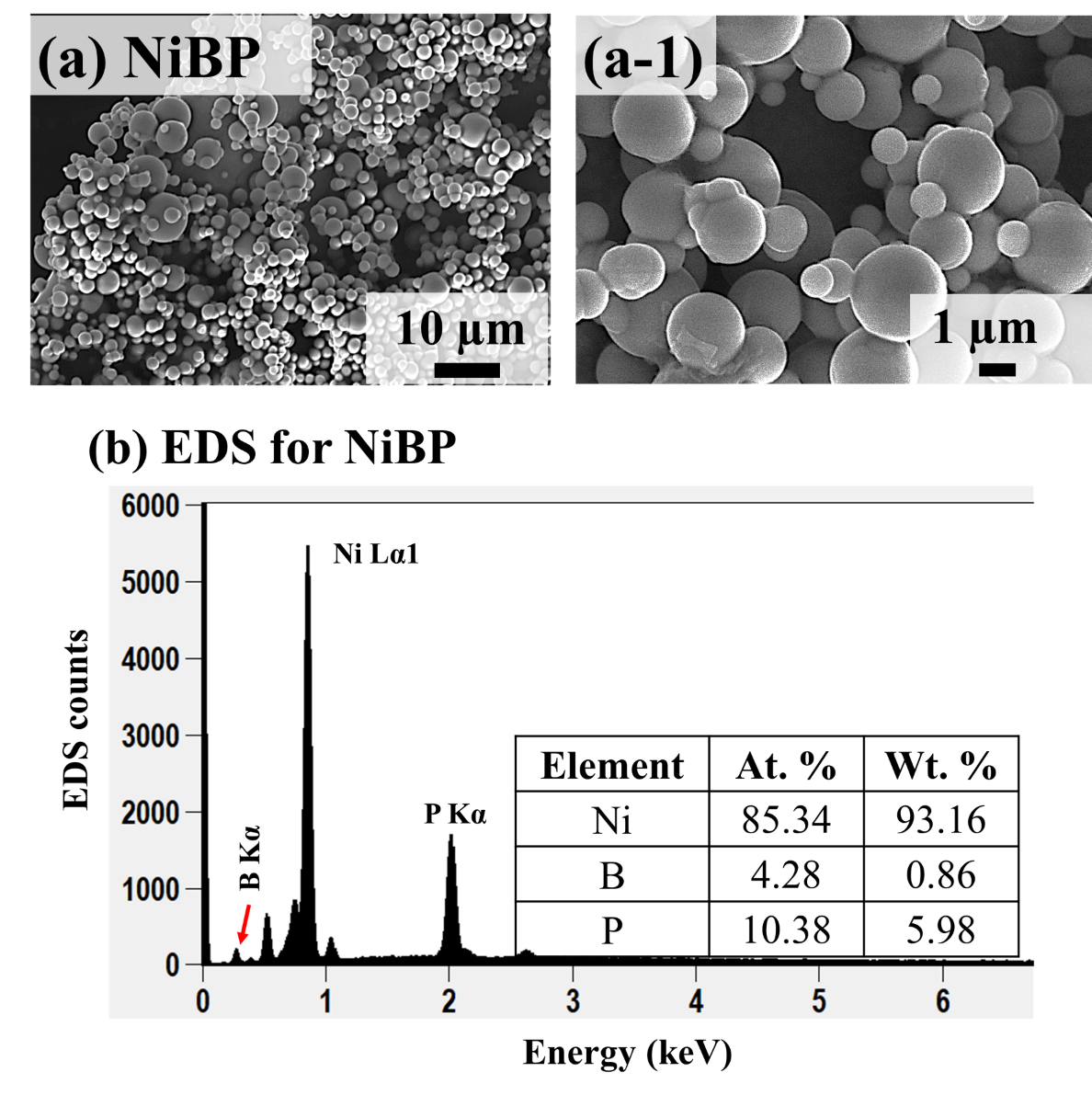


**Figure S7**. NiBP micro sphere (MS) template electrode. The electrode was fabricated by hydrothermal reaction at 180 ^o^C 12 hours with 1 mM Ni, 3.6 mM B**,** 8.4 mM P, 10 mM urea and 10 mM NH_4_F ^[17]^. (a) & (a-1) SEM image of NiBP MS base electrode. (b) EDS spectrum with the atomic %. More details about the fabrication process can be found in SI text S-1.1.

**S-2.2.2. NiBP base-electrode: Raman & XRD**


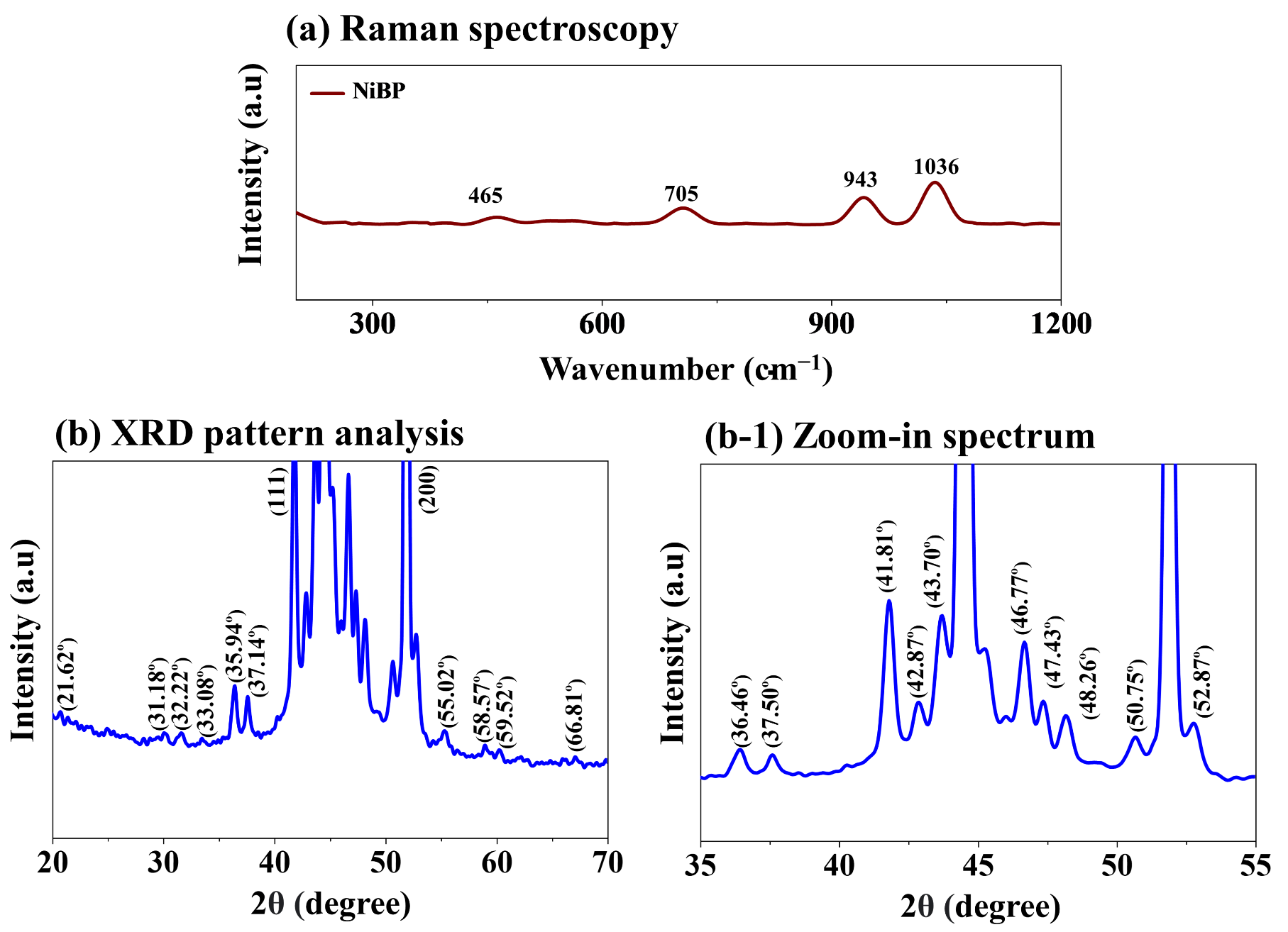


**Figure S8.** Raman & XRD analysis on NiBP electrode. (a) Raman spectroscopy analysis on NiBP. (b) XRD pattern of NiBP electrode. (b-1) Zoom in view between 35 and 55^o^. More detailed descriptions can be found in SI text S-1.6. NiBP base electrode characterizations.

**S-2.2.3. NiBP base-electrode: HER/OER & EIS**


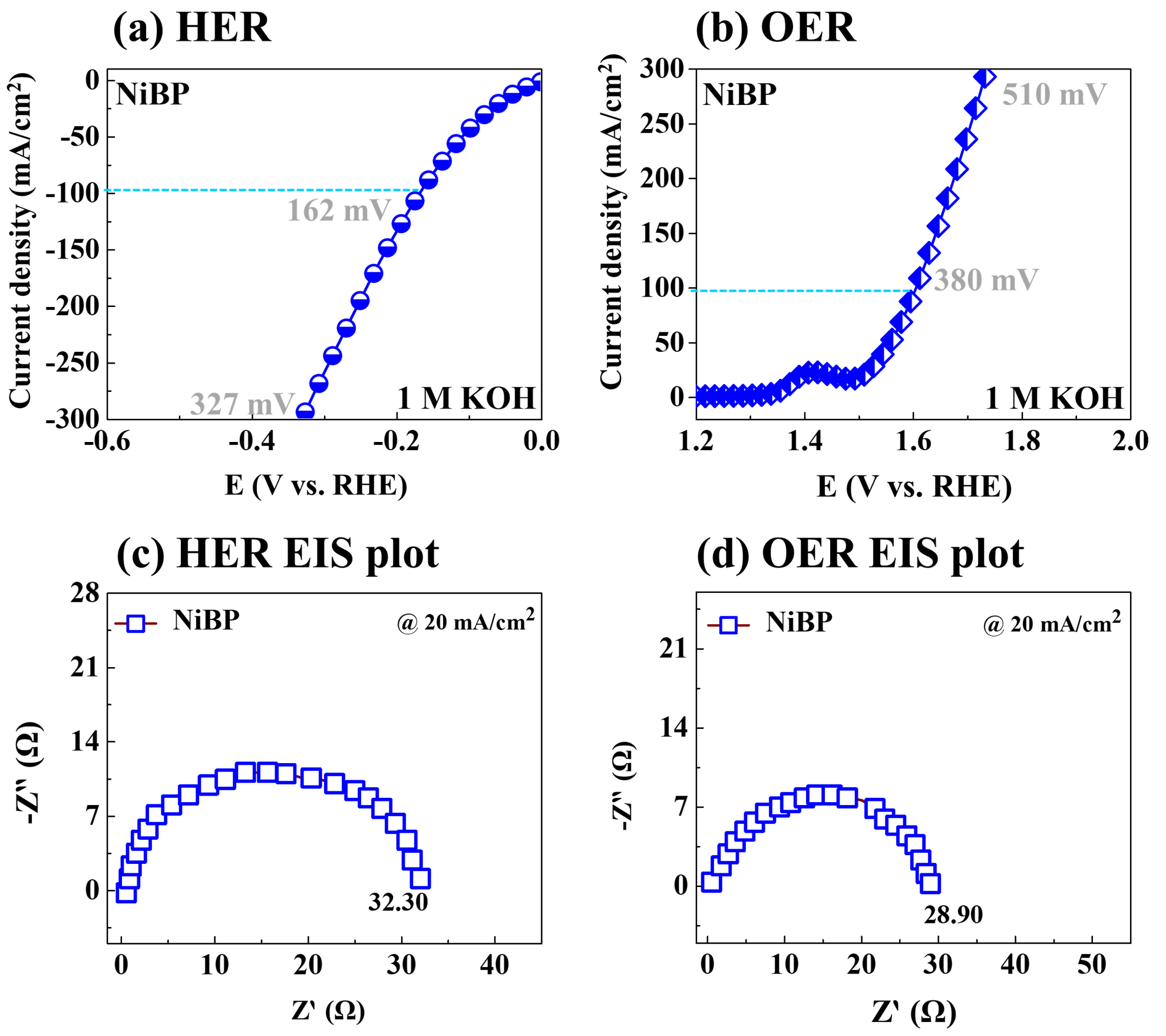


**Figure S9.** Electrochemical LSV and EIS analysis on NiBP electrode. (a) & (b) HER/OER LSV performance of NiBP in 1 M KOH. (c) & (d) HER/OER EIS plots.

**S-2.2.4. NiBP base-electrode: CV curves & current plot**


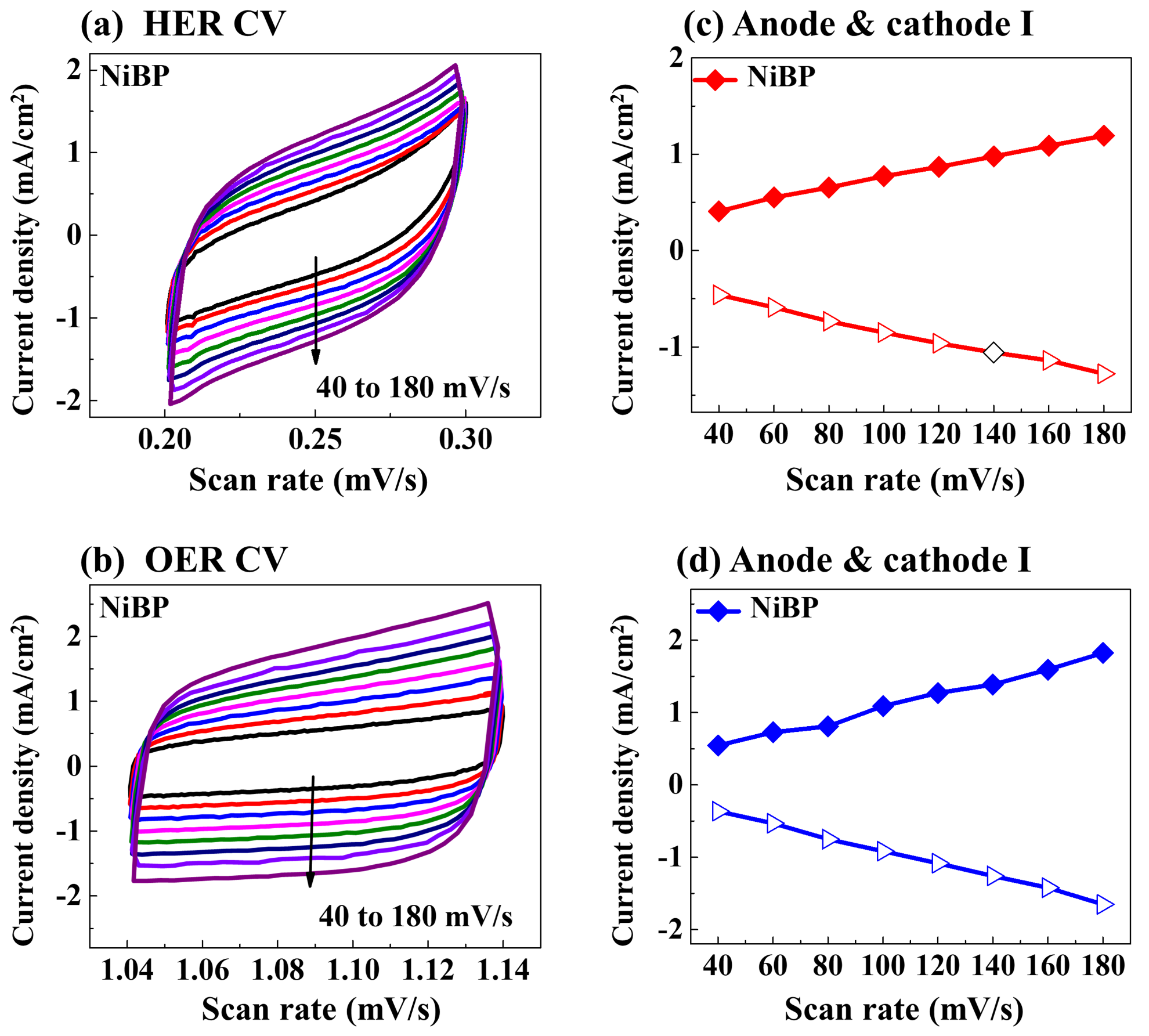


**Fig. S10.** (a) & (b) HER and OER CV curves of NiBP MSs. (c) & (d) HER and OER C_dl_ plots derived from anodic and cathodic current density plots versus scan rates.

**S-2.2.5. NiBP base-electrode: C_dl_ values & ECSA**


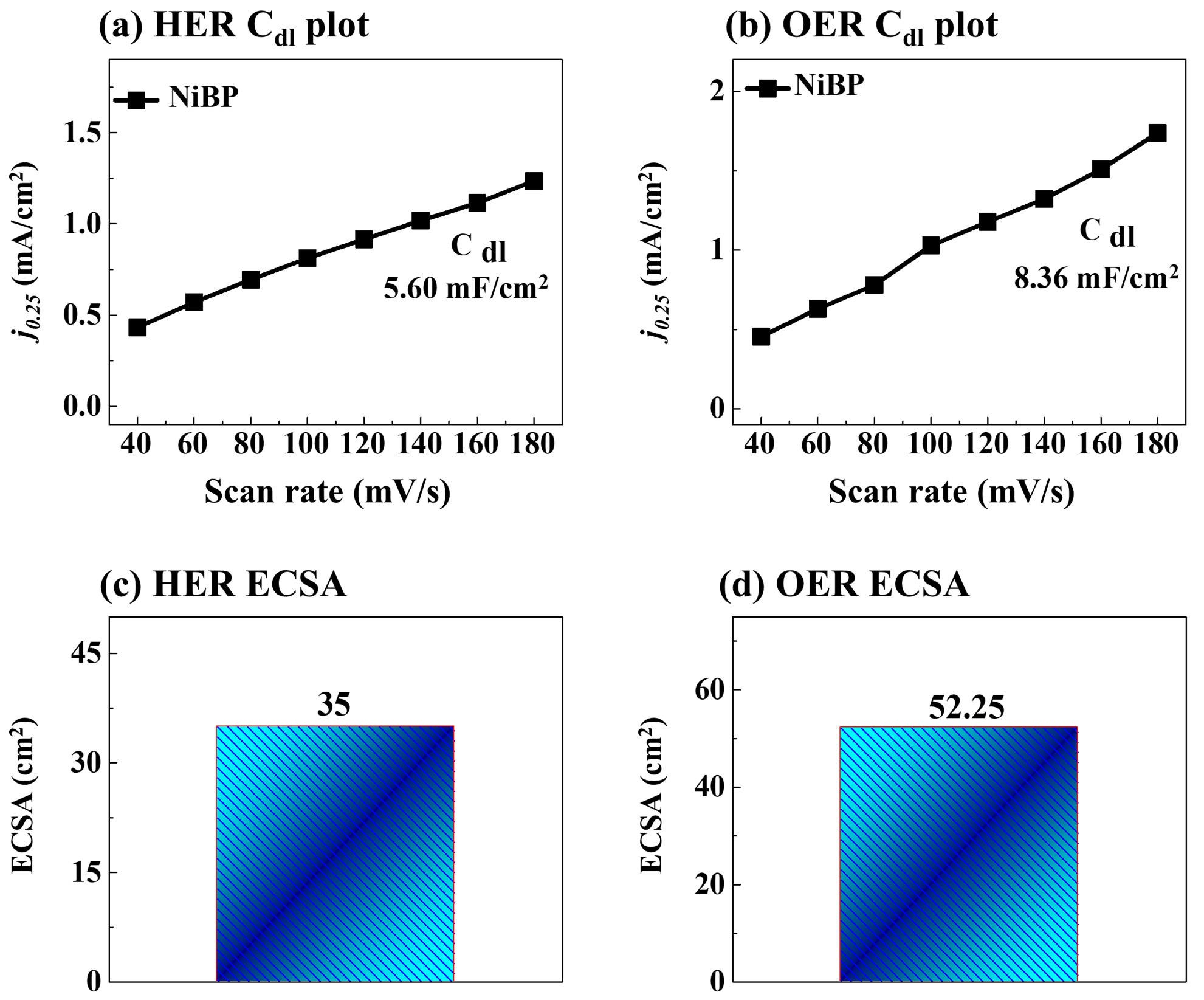


**Fig. S11.** (a) & (b) HER and OER C_dl_ plots of NiBP electrodes derived from anodic and cathodic current density plots versus scan rates. (c) & (d) HER and OER electrochemical surface area (ECSA) plots.

**S-2.3. Optimization of Cr doping by hydrothermal reaction**

**S-2.3.1. Cr doping: Cr concentration control**


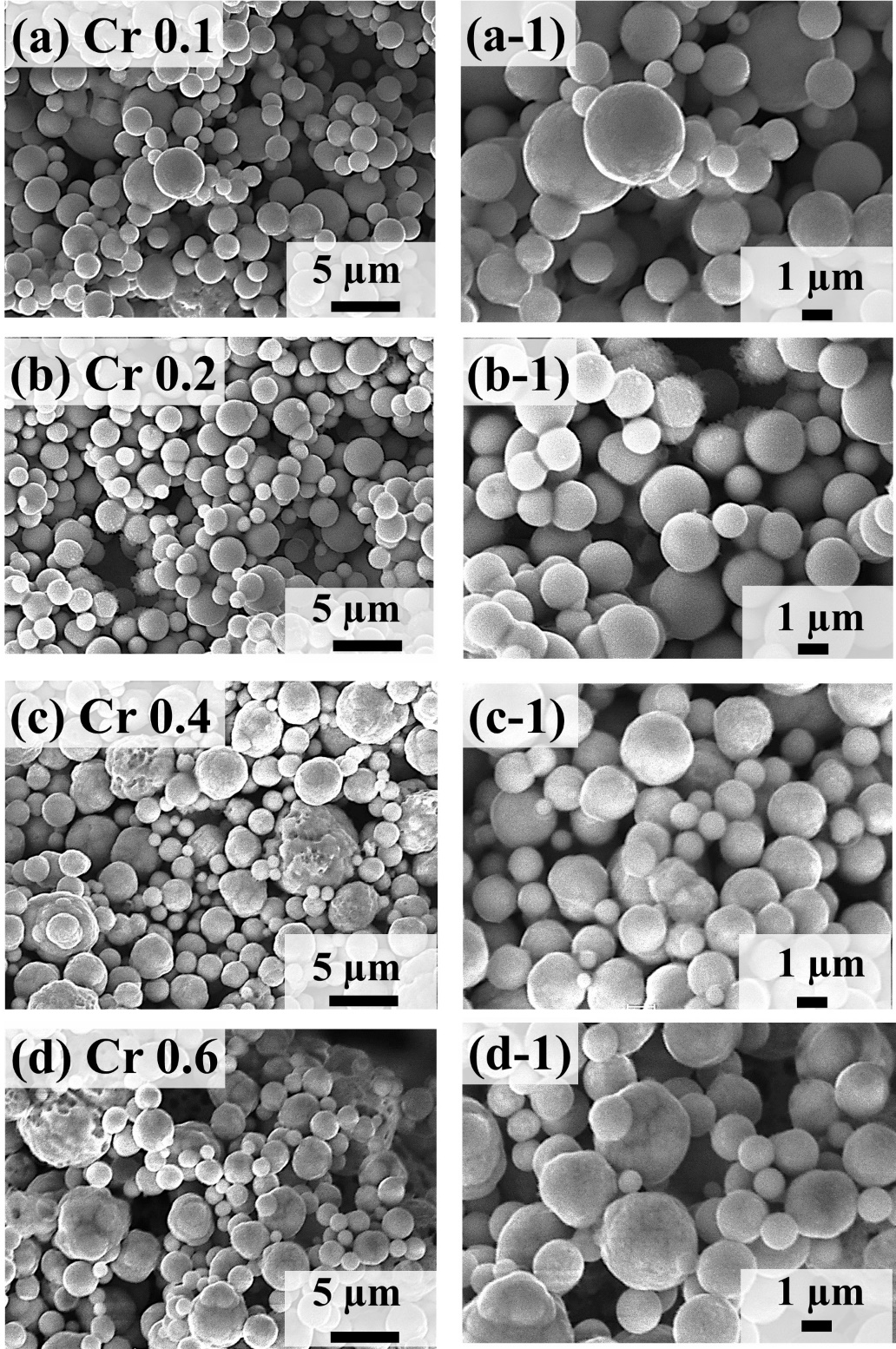


**Figure S12.** Cr concentration variation for Cr/NiBP MS electrocatalysts fabrication. Cr concentration was controlled between 0.1 ~ 0.6 mmol on NiBP at 180^o^ C for 12 hours. (a) – (d) SEM images. (a-1) – (d-1) Enlarged views.

**S-2.3.1. Cr doping: Cr concentration control**


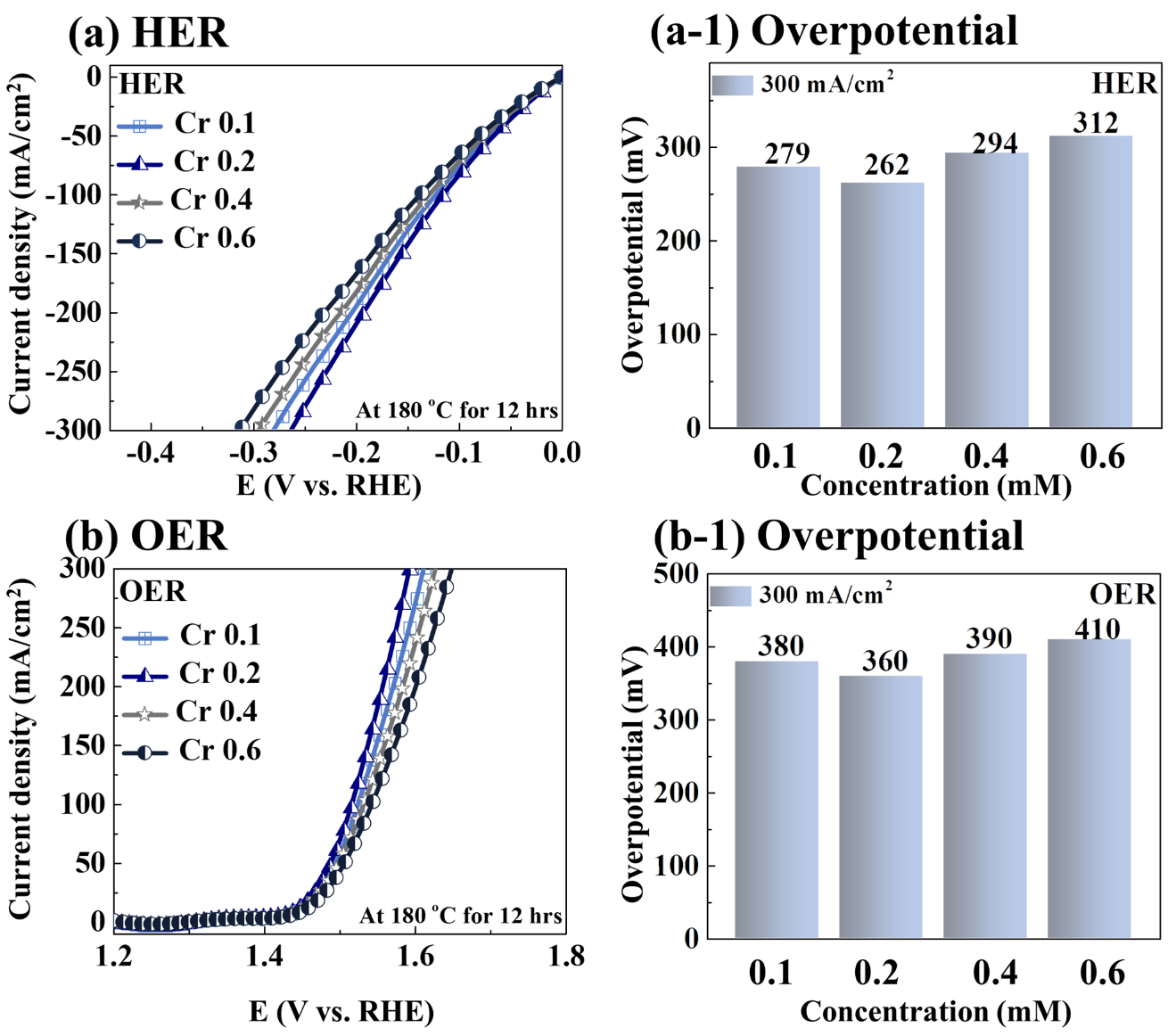


**Figure S13.** Electrochemical HER/OER performances of Cr/NiBP electrodes with Cr concentration variation set. (a) – (b) HER and OER LSV polarization curves in 1 M KOH. (a-1) – (b-1) Overpotential bar graphs at 300 mA/cm^2^. **0.2 mM Cr-doped Cr/NiBP electrode demonstrated the best HER and OER performances with the lowest overpotential values.**

**S-2.3.2. Cr doping: Reaction temperature control (at 12 hours)**


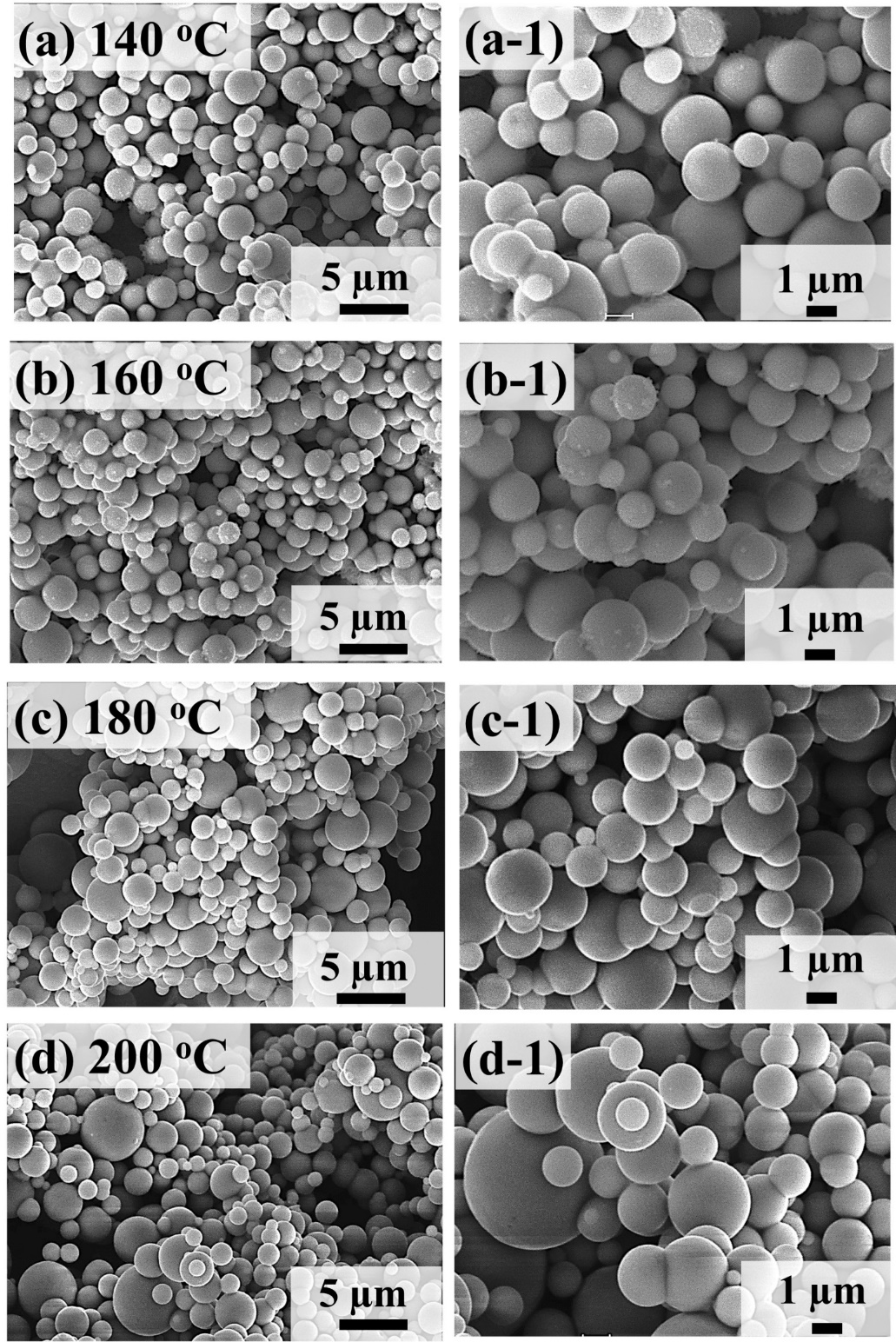


**Figure S14.** Hydrothermal reaction temperature variation for Cr/NiBP MS electrocatalysts fabrication. Hydrothermal reaction temperature was varied between 140 ~ 200 ℃ with 0.2 mM Cr for 12 hours. (a) – (d) SEM images. (a-1) – (d-1) Enlarged views.

**S-2.3.2. Cr doping: Reaction temperature control**


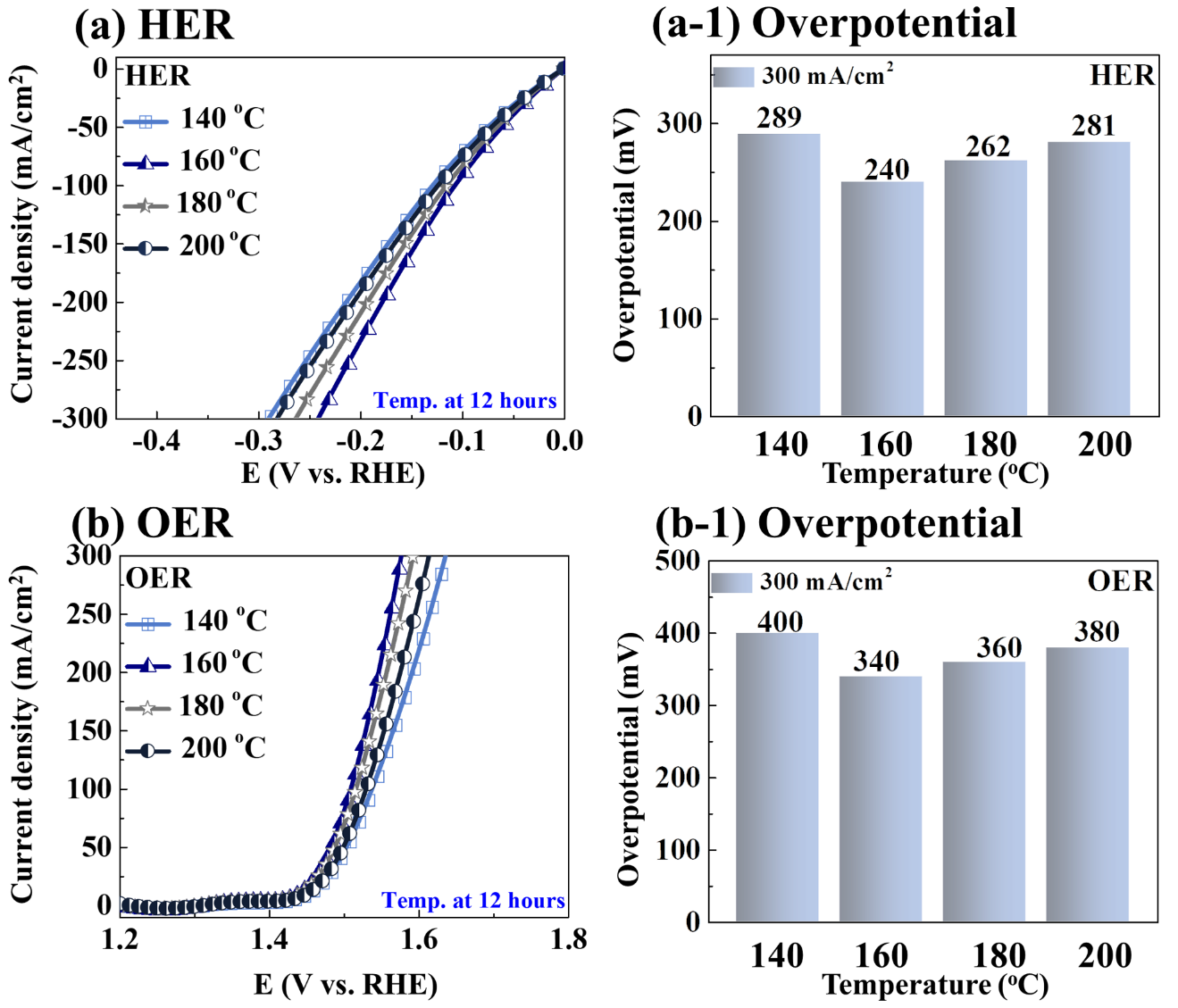


**Figure S15.** HER/OER performances of Cr/NiBP electrodes with reaction temperature variation set. (a) – (b) HER and OER LSV polarization curves in 1 M KOH. (a-1) – (b-1) Overpotential bar graphs at 300 mA/cm^2^. **160 ^o^C fabricated Cr/NiBP electrode demonstrated the best HER and OER performances in terms of the overpotentials.**

**S-2.3.3. Cr doping: Reaction duration control (at 160 ^o^C)**


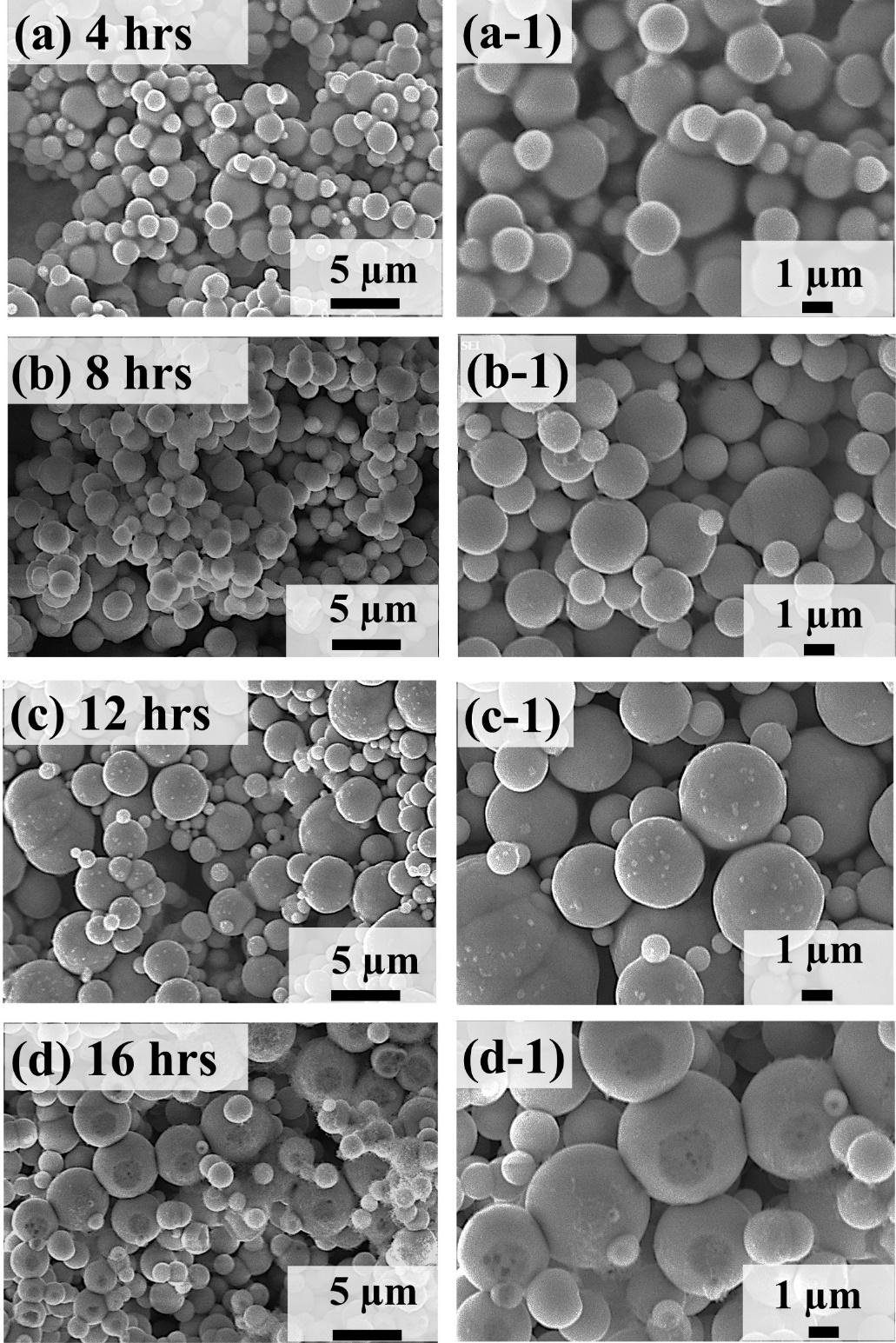


**Figure S16.** Hydrothermal reaction duration variation for Cr/NiBP MS electrocatalysts fabrication. The reaction durations varied between 4 ~ 16 hours at 160 ^o^C. (a) – (d) SEM images. (a-1) – (d-1) Enlarged views.

**S-2.3.3. Cr doping: Reaction duration control (at 160 ^o^C)**


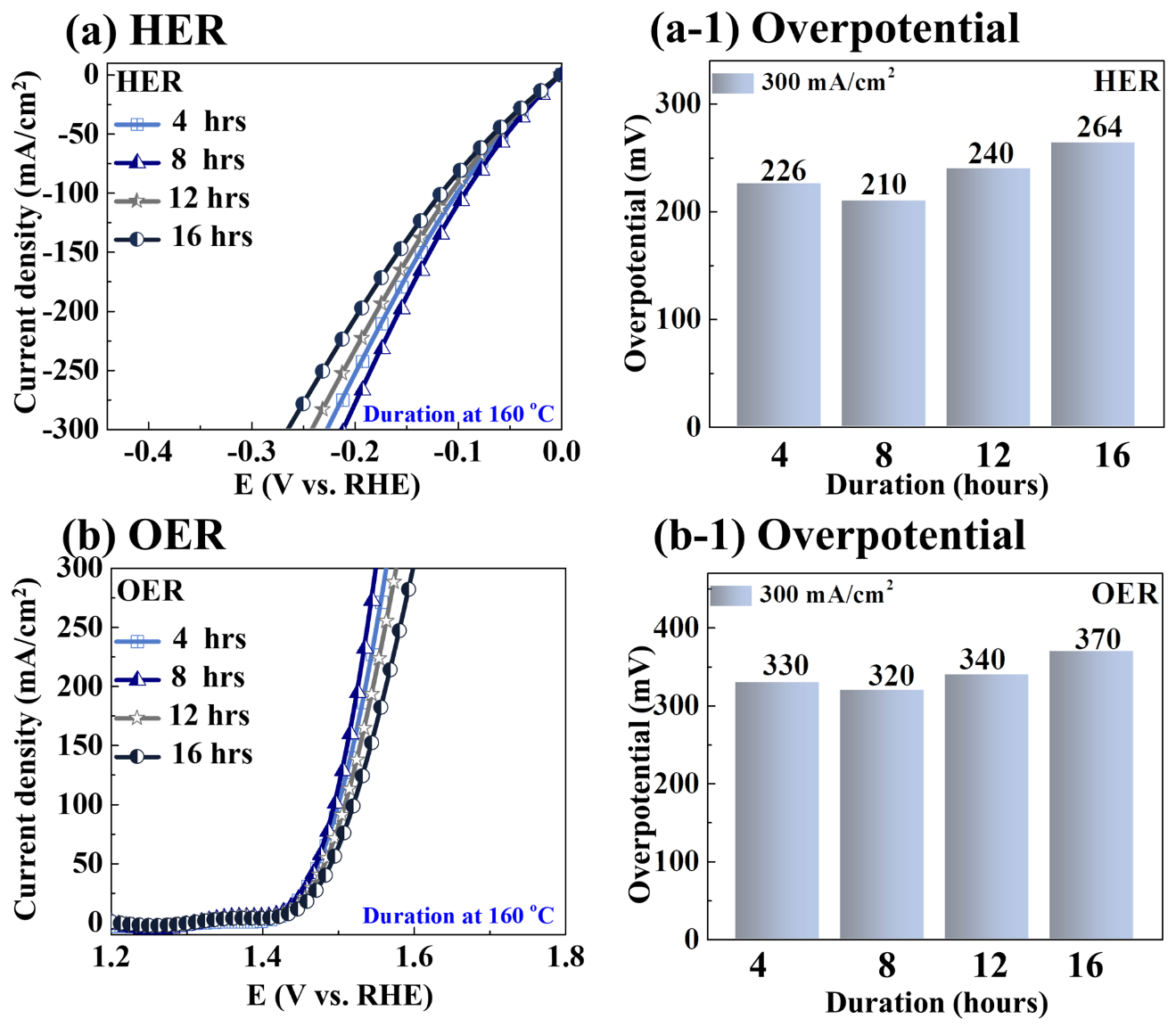


**Figure S17.** Electrochemical HER/OER performance of Cr/NiBP electrodes with reaction duration variation at 160 ^o^C. (a) – (b) HER and OER LSV polarization curves in 1 M KOH. (a-1) – (b-1) Overpotential bar graph summary at 300 mA/cm^2^. **8-hour reaction duration fabricated Cr/NiBP demonstrated the best HER and OER performance in this set.**

**S-2.3.4. Cr doping: Reaction temperature control (for 8 h)**


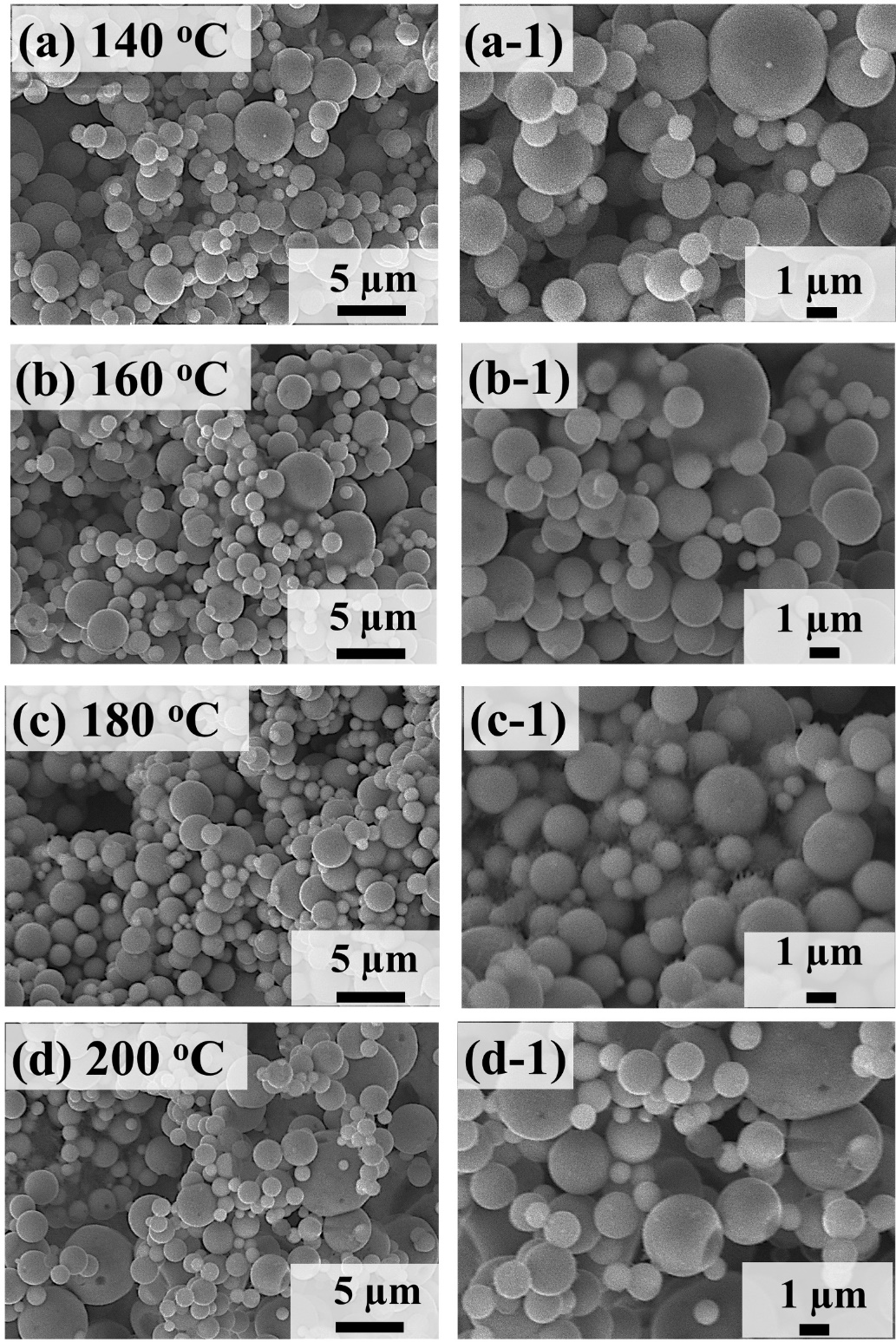


**Figure S18.** Hydrothermal reaction temperature variation with optimized 8-hour reaction for Cr/NiBP MS electrocatalysts fabrication. The reaction temperatures were varied between 140 ~ 200 ℃ with 0.2 mM Cr for 8 hours. (a) – (d) SEM images. (a-1) – (d-1) Enlarged views.

**S-2.3.4. Cr doping: Reaction temperature control (for 8 h)**


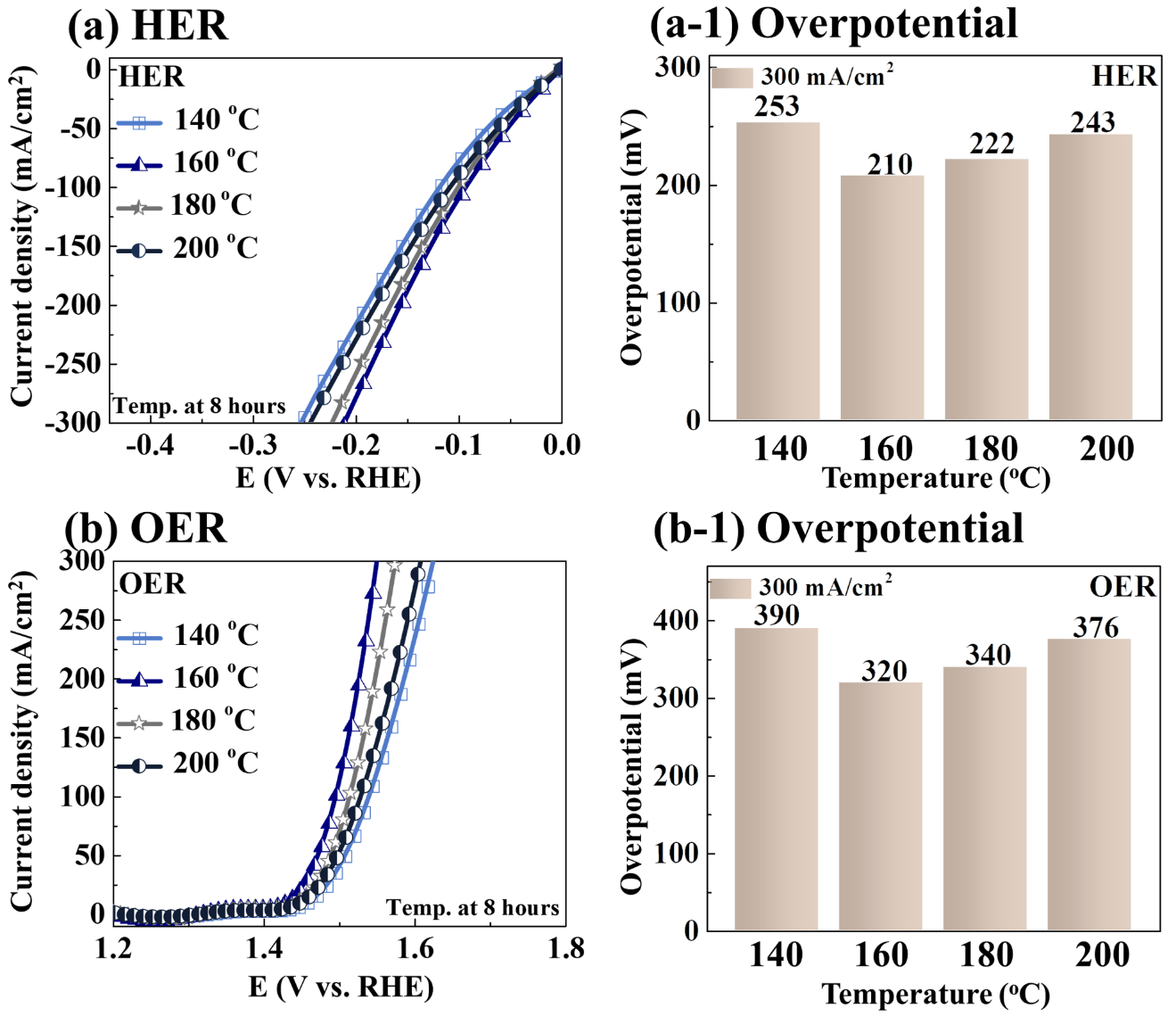


**Figure S19.** Electrochemical HER/OER performance of Cr/NiBP electrodes with reaction temperature variation at optimized 8 hours duration. (a) – (b) HER and OER LSV polarization curves in 1 M KOH. (a-1) – (b-1) Overpotential summary at 300 mA/cm^2^. **160 ^o^C fabricated Cr/NiBP demonstrated the best HER and OER performance in this set.**

**S-2.3.5. Cr doping: Cr con. control (at 160 ^o^C for 8 h)**


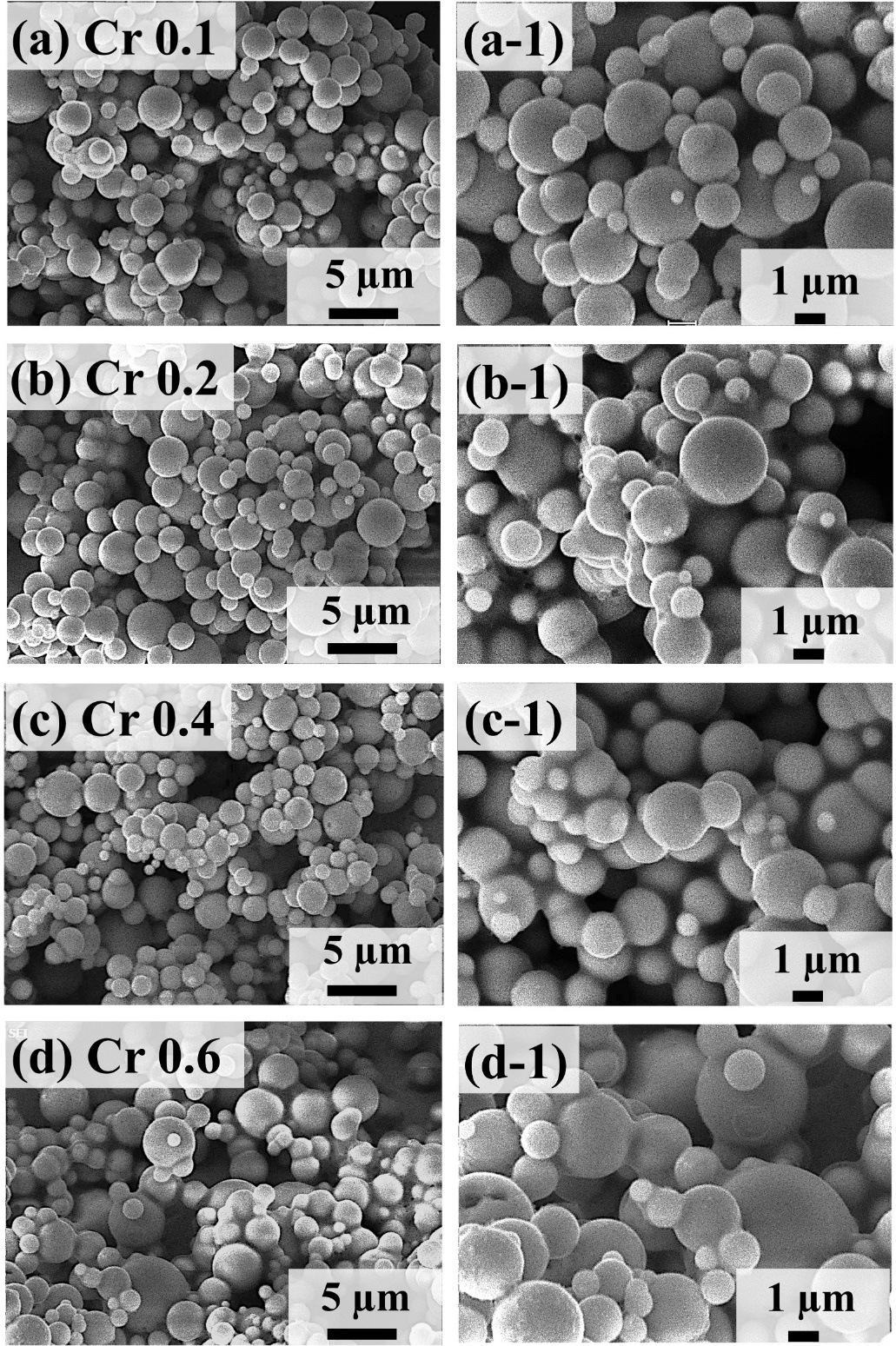


**Figure S20**. Cr concentration variation between 0.1 ~ 0.6 mmol (2^nd^ time-varied) with optimized hydrothermal reaction duration 8-hour and 160 ^o^C temperature for Cr/NiBP MS electrocatalysts fabrication. (a) – (d) SEM images. (a-1) – (d-1) Enlarged views.

**S-2.3.5. Cr doping: Cr con. control (at 160 ^o^C for 8 h)**


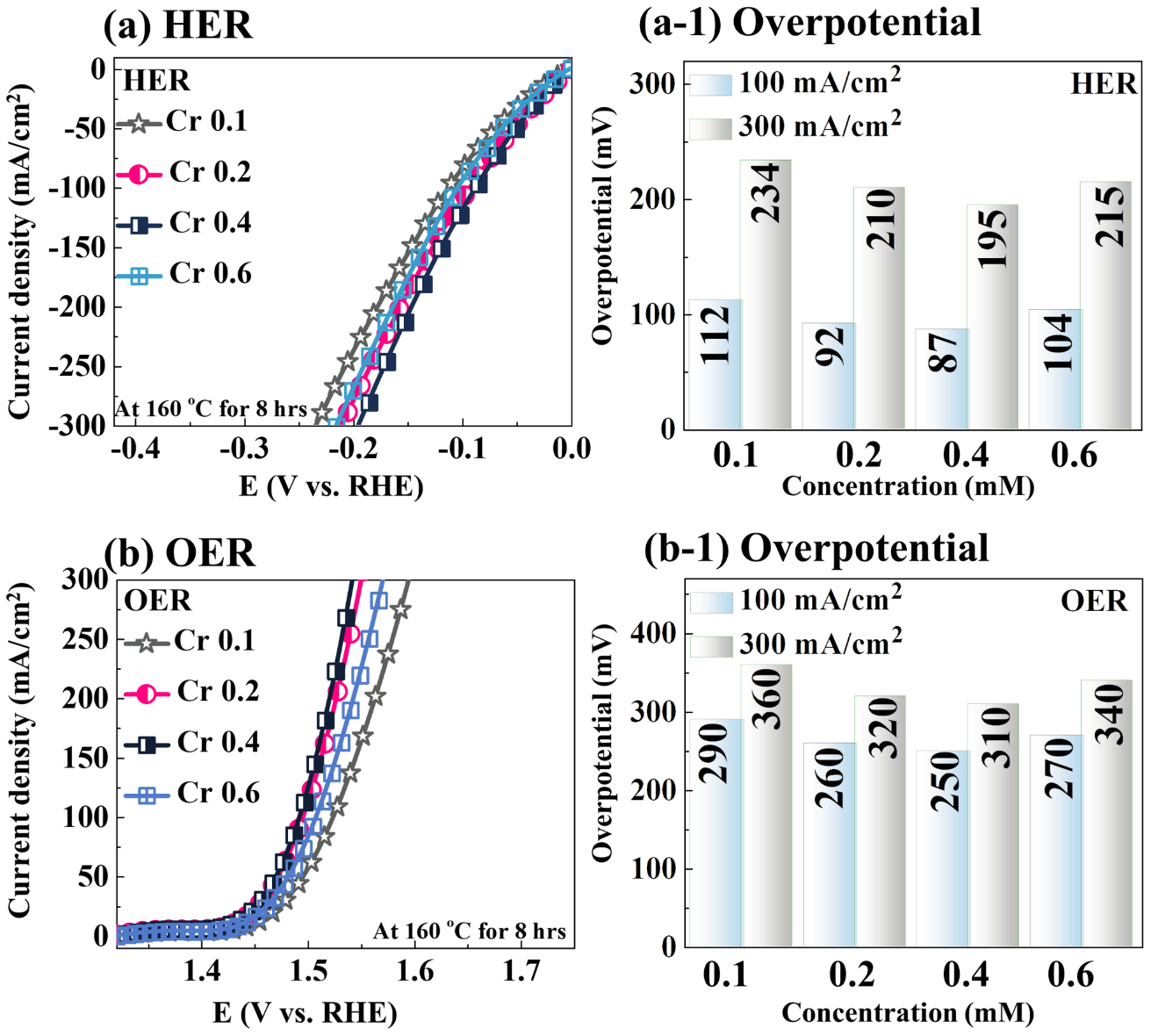


**Figure S21.** Electrochemical HER/OER activity of Cr/NiBP MS electrocatalysts with the Cr concentration variation at 160 ℃ for 8 h reaction. (a) – (b) HER and OER LSV polarization curves in 1 M KOH. (a-1) – (b-1) Overpotential bar graphs at 300 mA/cm^2^. **The 0.4 mM Cr-doped fabricated Cr/NiBP demonstrated the best HER and OER performance in this set.** The best Cr/NiBP (Cr doping at 0.4 mM at 160 ^o^C for 8 h) was introduced further for post-annealing.

**S-2.3.6. Post-annealing temperature optimization: SEM**


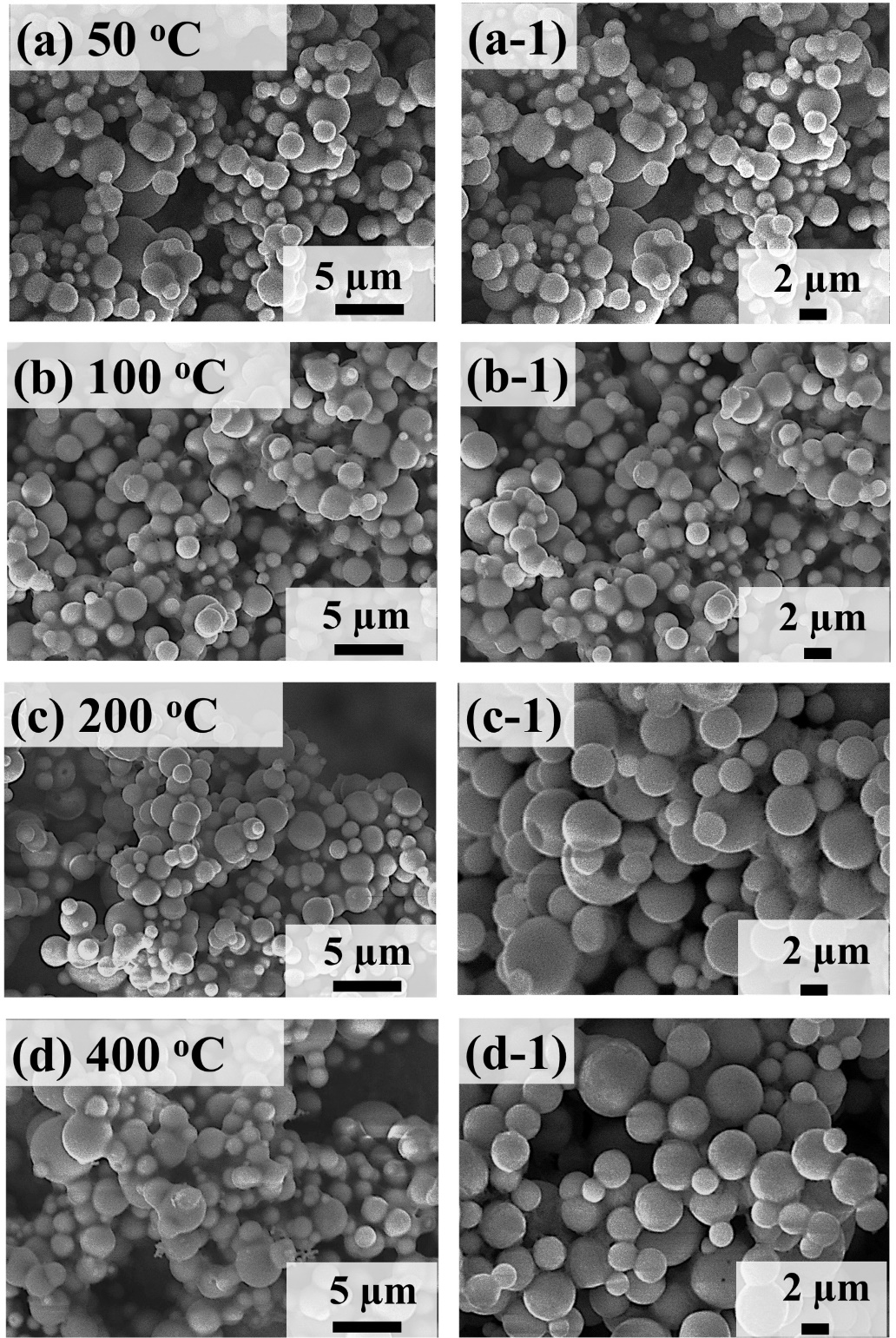


**Figure S22**. Post-annealing temperature optimization for Cr/NiBP MS electrocatalysts fabrication. The post-annealing temperature was controlled between 50 ~ 100 ^o^C for 30 min. (a) – (d) SEM images. (a-1) – (d-1) Enlarged view.

**S-2.3.6. Post-annealing temperature optimization: LSV**


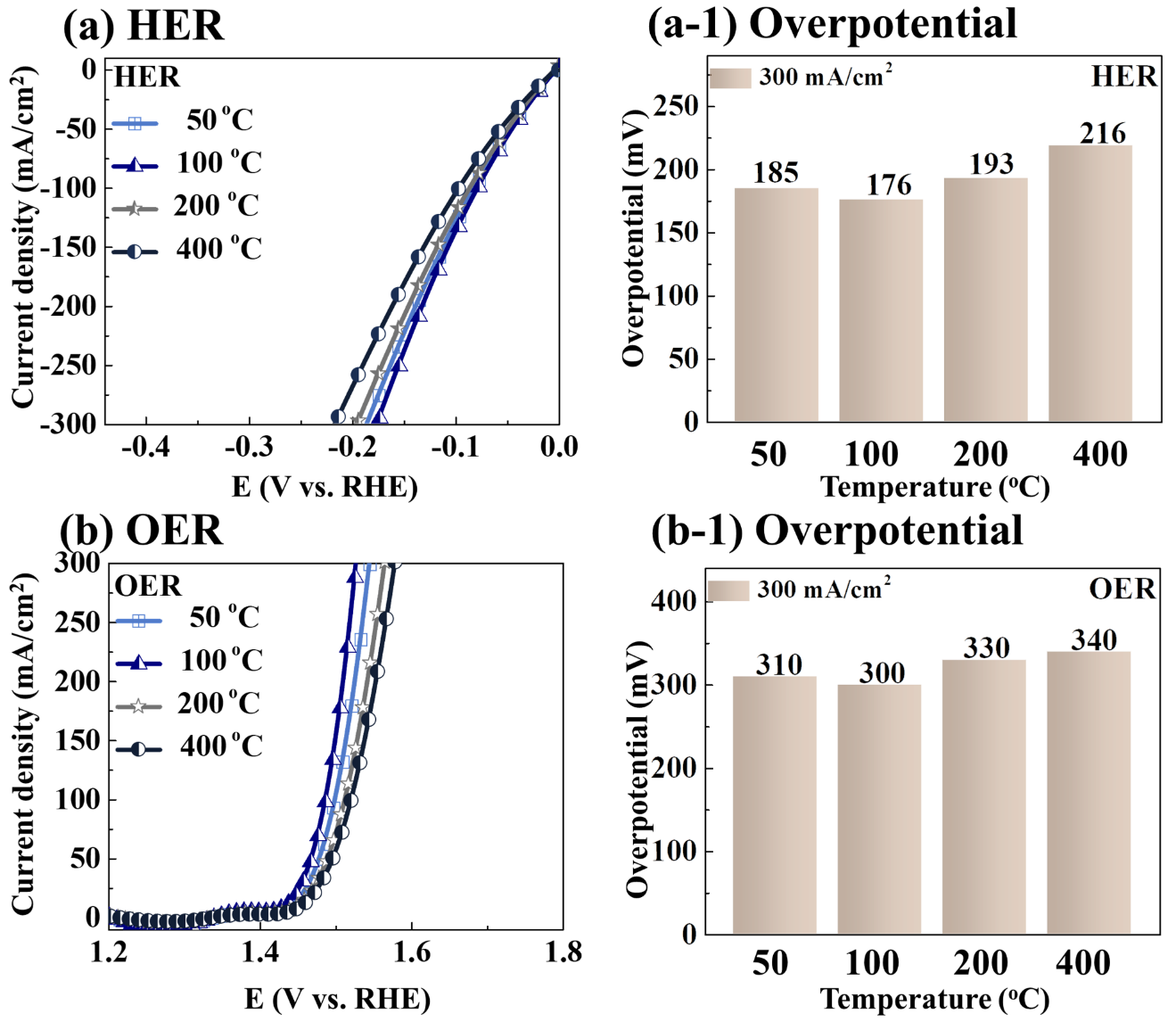


**Figure S23.** Electrochemical HER/OER performance of Cr/NiBP electrodes with post-annealing temperature variation. (a) – (b) HER and OER LSV polarization curves in 1 M KOH. (a-1) – (b-1) Overpotential bar graphs at 300 mA/cm^2^. **The 100 ^o^C annealed Cr/NiBP electrode demonstrated the best HER and OER performance in this set.**

**S-2.3.7. Post-annealing duration optimization: SEM**


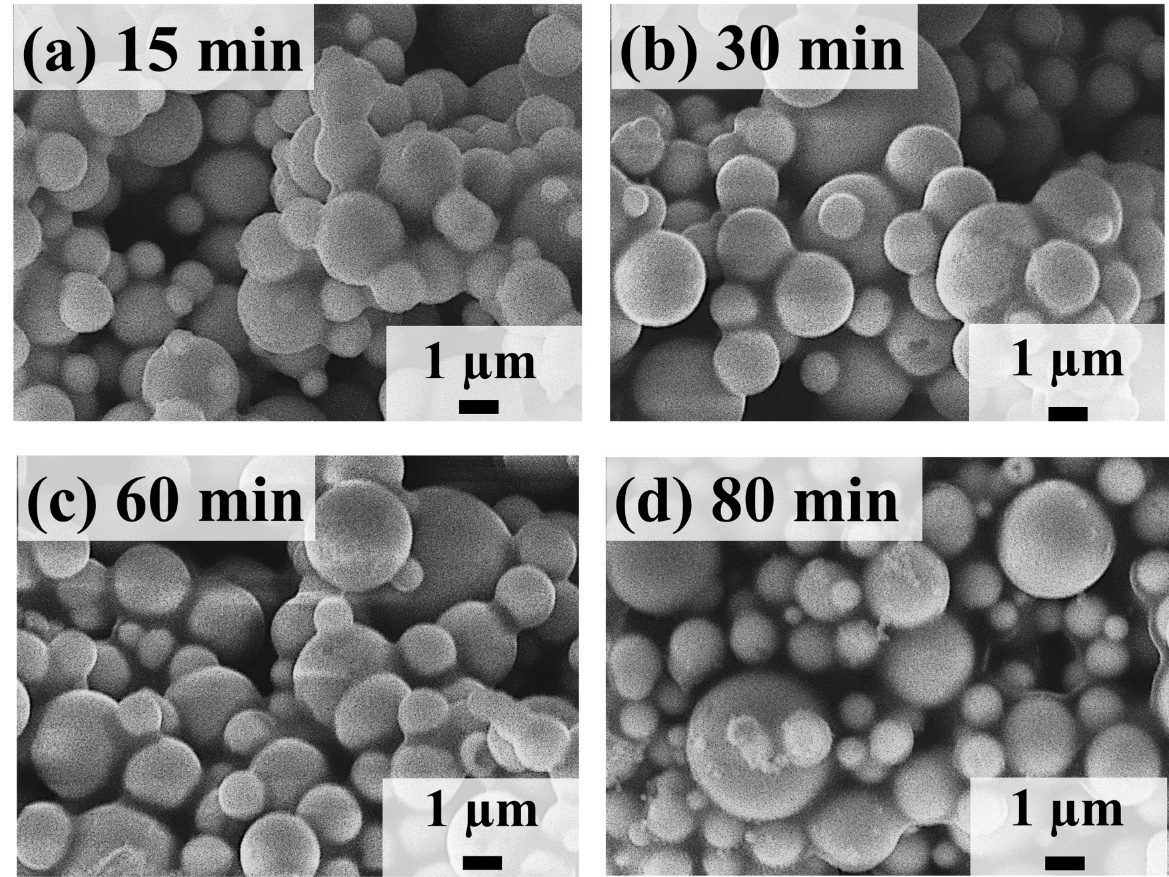


**Figure S24**. Annealing duration optimization for Cr/NiBP MS electrocatalysts. The optimized Cr/NiBP electrode was annealed between 15 ~ 80 min at 100 ^o^C. (a) – (d) SEM images.

**S-2.3.7. Post-annealing duration optimization: EDS**


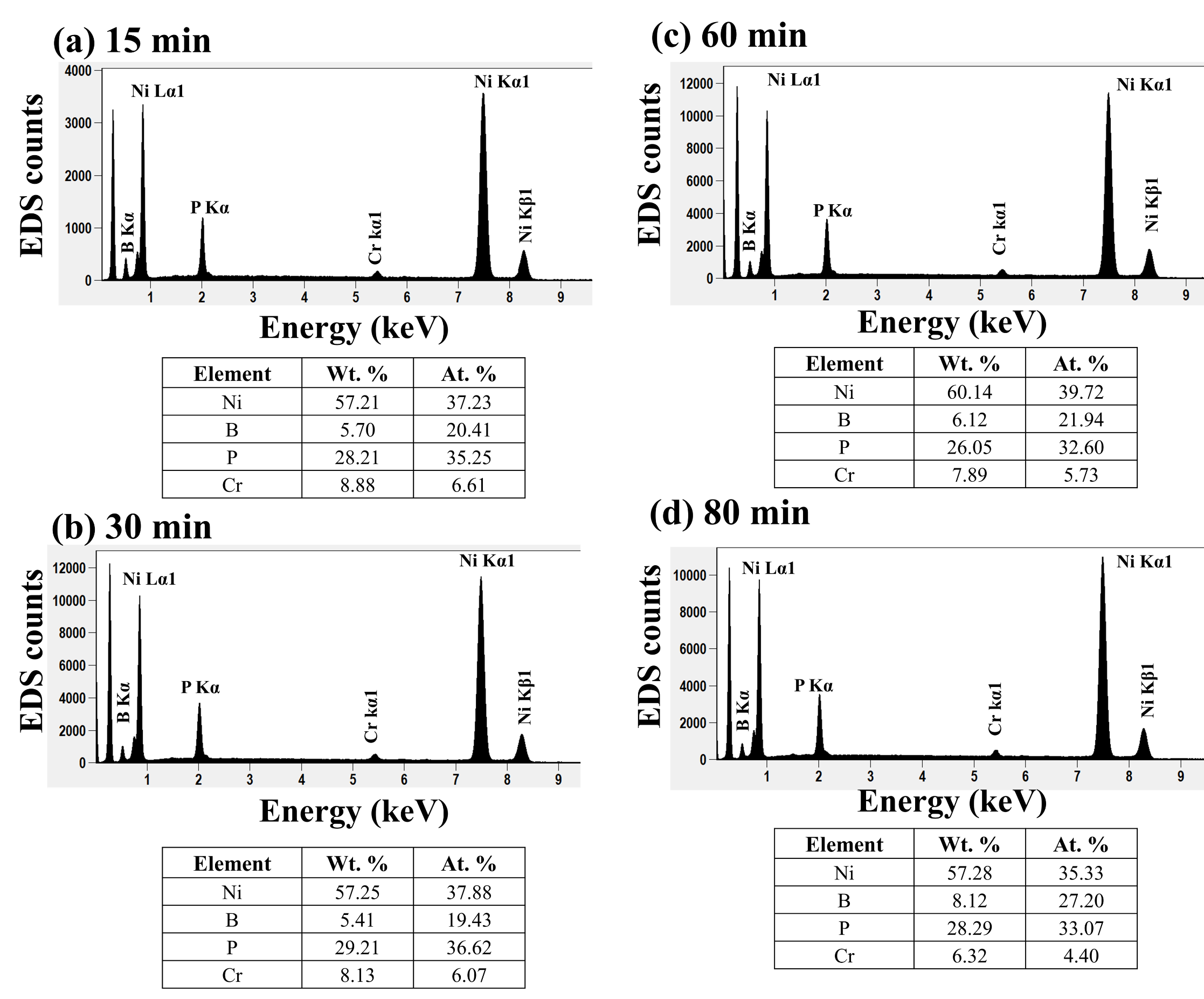


**Figure S25.** EDS spectra of Cr/NiBP MS electrocatalysts with the post-annealing duration optimization set. (a) – (d) EDS spectra, corresponding elemental weight and atomic percentages without NF substrate. The Cr/NiBP particles were transferred from nickel foam (NF) substrate to carbon types to obtain actual quantitative analysis in EDS measurements.

**S-2.3.7. Post-annealing duration optimization: HER CV**


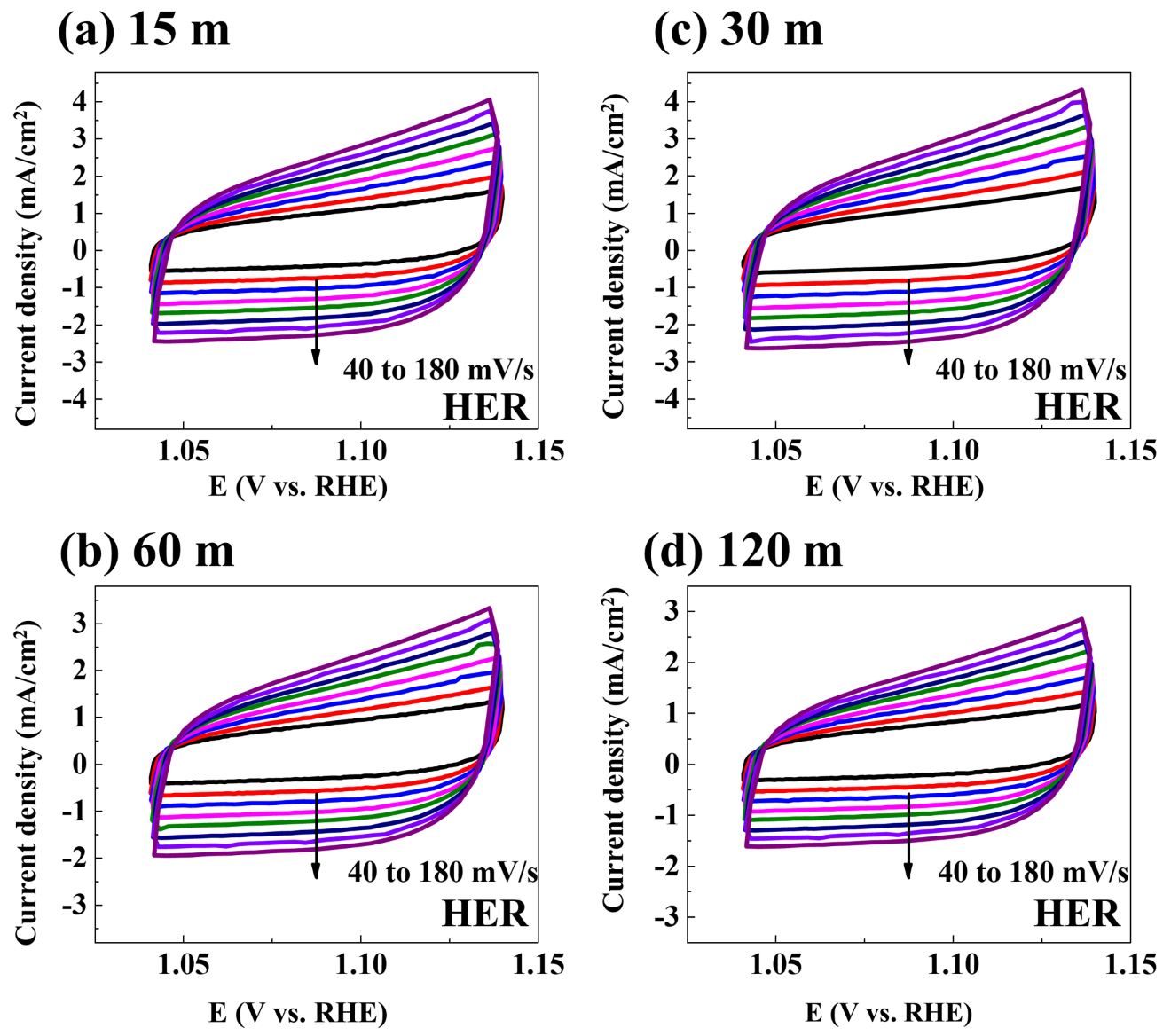


**Figure S26.** HER cyclic voltammetry (CV) profiles of Cr/NiBP electrocatalysts in post-annealing duration variation set at different scan rates ranging from 40 – 180 mVs^-1^. (a) – (d) HER CV curves.

**S-2.3.7. Post-annealing duration optimization: OER CV**


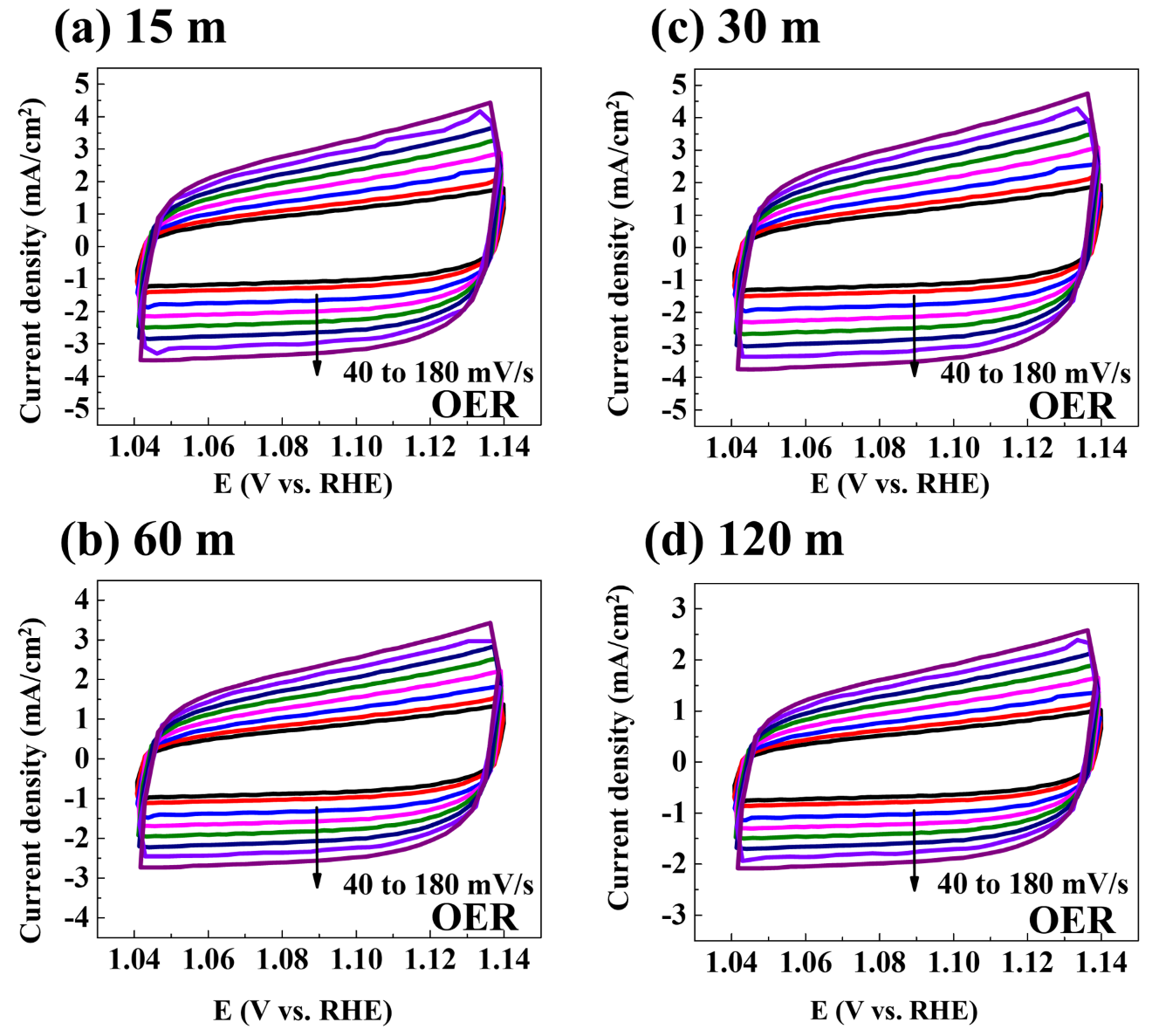


**Figure S27.** OER CV profiles of Cr/NiBP electrodes in post-annealing duration variation set. (a) – (d) OER CV curves.

**S-2.3.7. Post-annealing duration optimization: C_dl_**


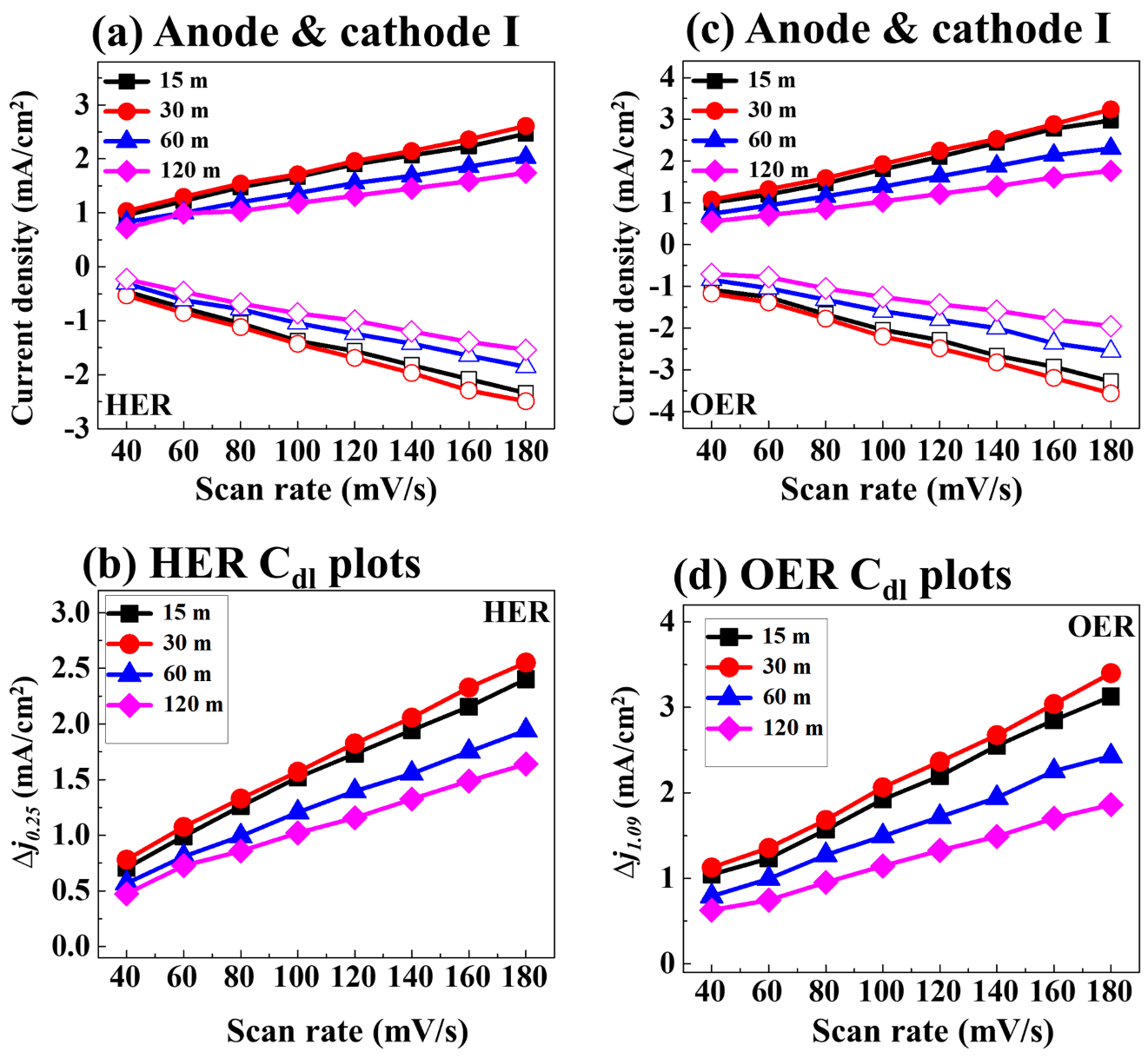


**Figure S28.** (a) & (c) HER and OER linear graph for the anodic and cathodic current density versus scan rates from CV curves. (b) & (d) Current density differences (anodic and cathodic) plotted versus various CV scan rates as the slope value gives as double-layer capacitance (C_dl_) values plots.

**S-2.3.7. Post-annealing duration optimization: ECSA**


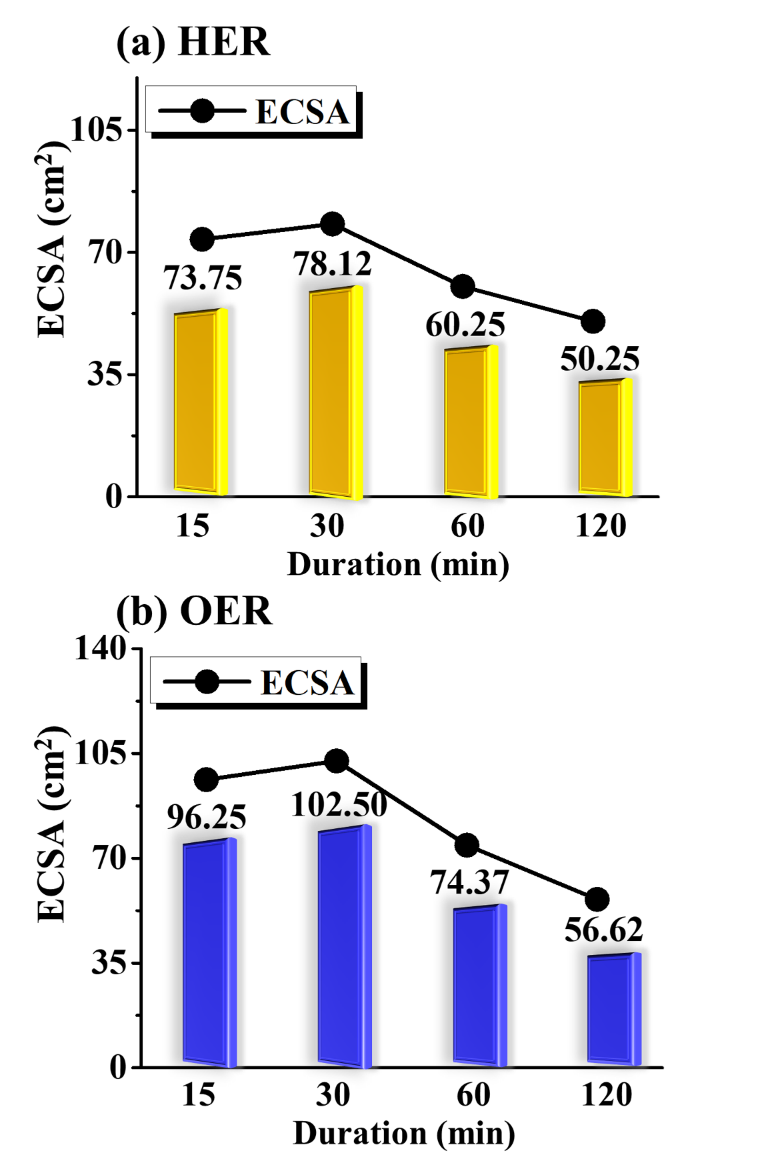


**Figure S29.** (a) & (b) Electrochemical active surface area (ECSA) HER & OER values of Cr/NiBP post-annealing duration variation set. A more detailed calculation process can be found in SI text S-1.2. Electrochemical characterizations.

**S-2.4. NiBP and Cr/NiBP comparison**

**S-2.4.1. NiBP and Cr/NiBP comparison: Raman**


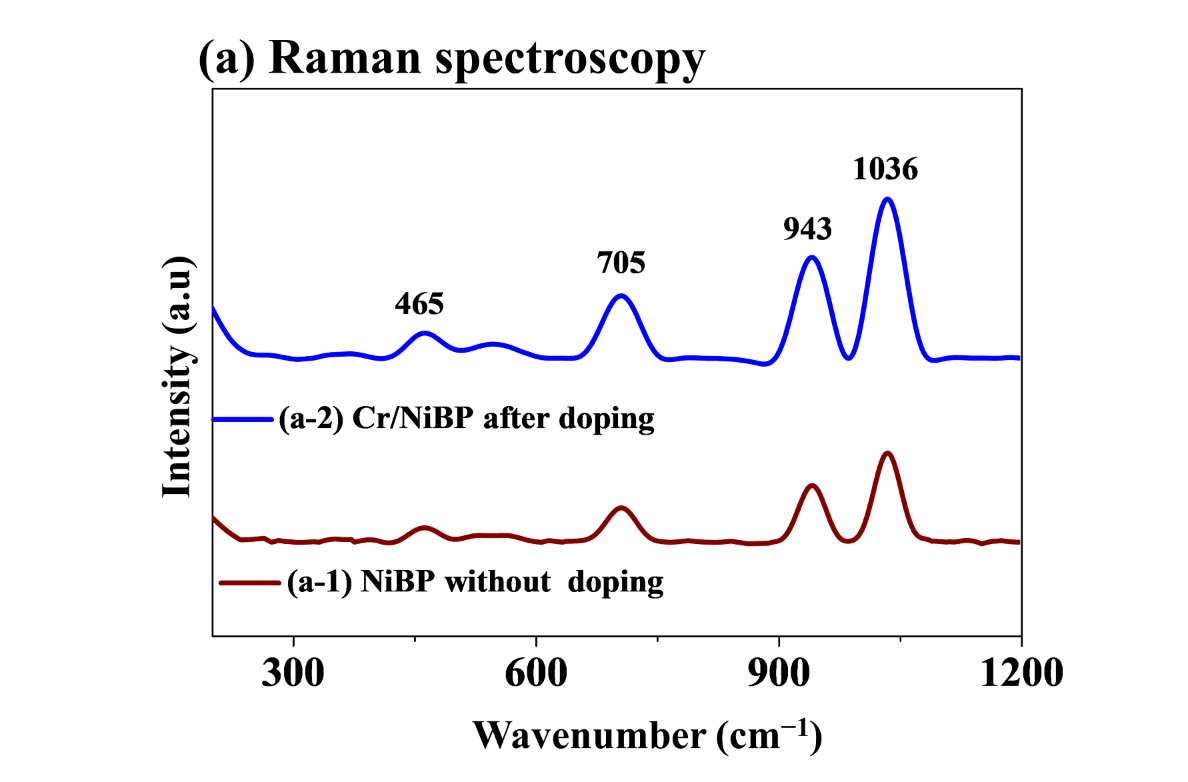


**Figure S30.** (a) Raman spectra comparison of bare NiBP and Cr/NiBP electrodes. More details about Raman analysis are provided in SI text S-1.7. Raman analysis of Cr/NiBP.

**S-2.4.2. NiBP and Cr/NiBP comparison: XRD**


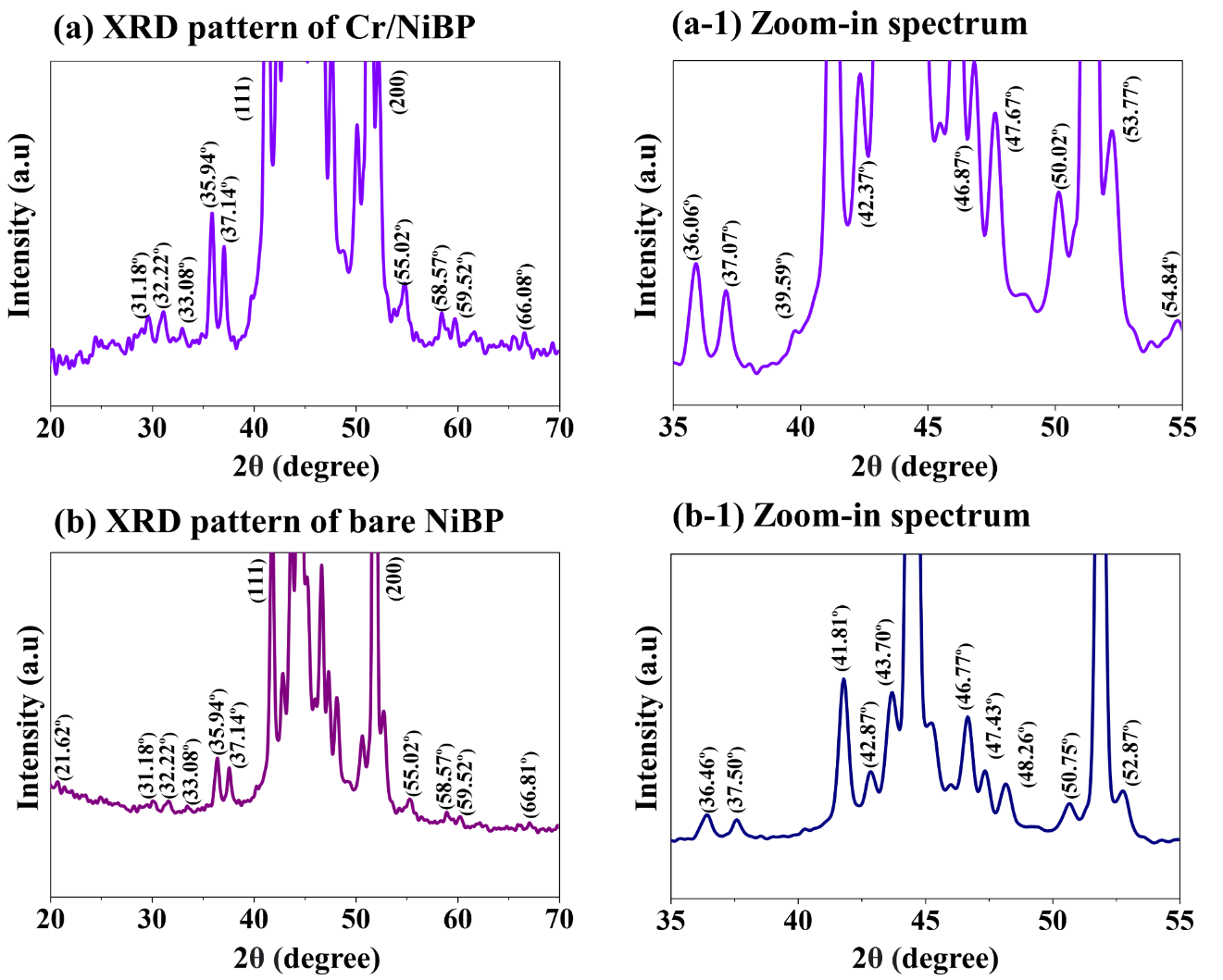


**Figure S31.** (a) & (b) X-ray diffraction (XRD) patterns comparison of bare NiBP and Cr/NiBP electrodes. (a-1) & (a-2) Zoom-in spectra in between 35 and 55^o^. More details discussion about XRD can be found in SI text S-1.8. X-ray diffraction (XRD) analysis.

**S-2.4.3. NiBP and Cr/NiBP comparison: EIS**


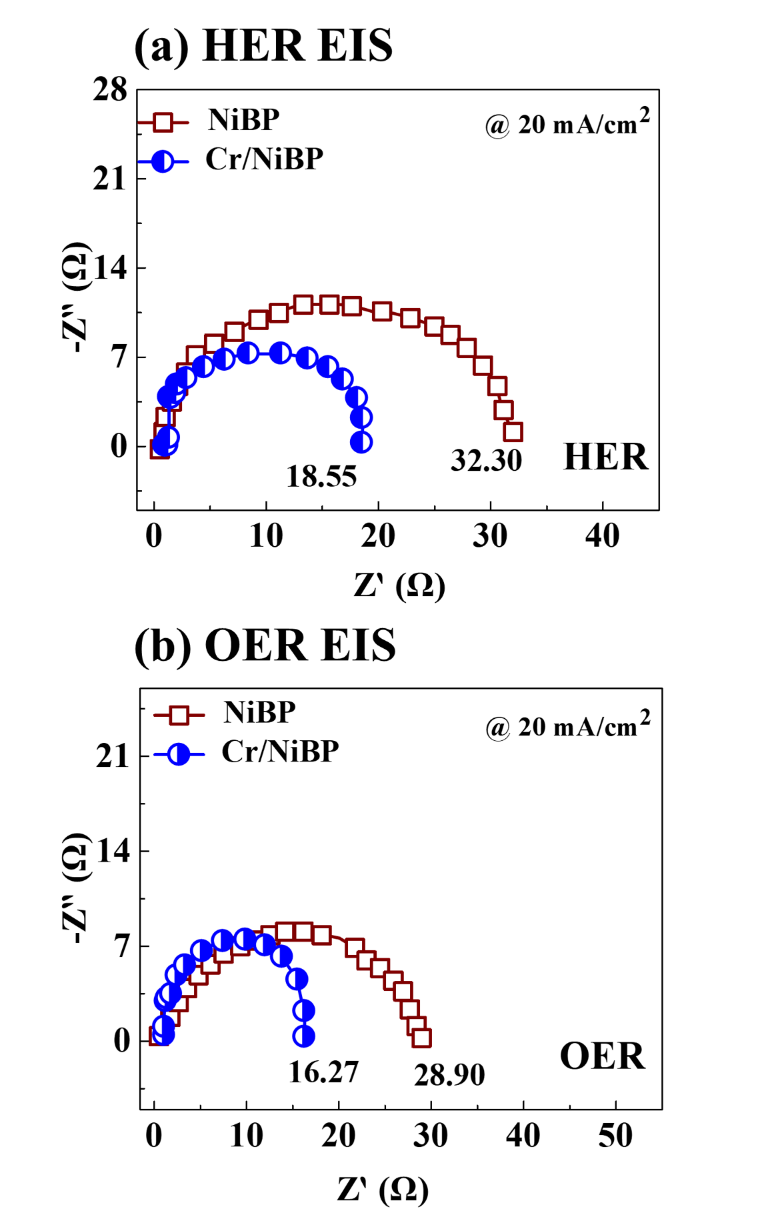


**Figure S32.** (a) & (b) HER and OER electrochemical impedance spectroscopy (EIS) comparison of bare NiBP and Cr/NiBP electrocatalyst. A more detailed discussion can be found in SI text S-1.13. Cr doping effect.

**S-2.4.4. NiBP and Cr/NiBP comparison: C_dl_ & ECSA**


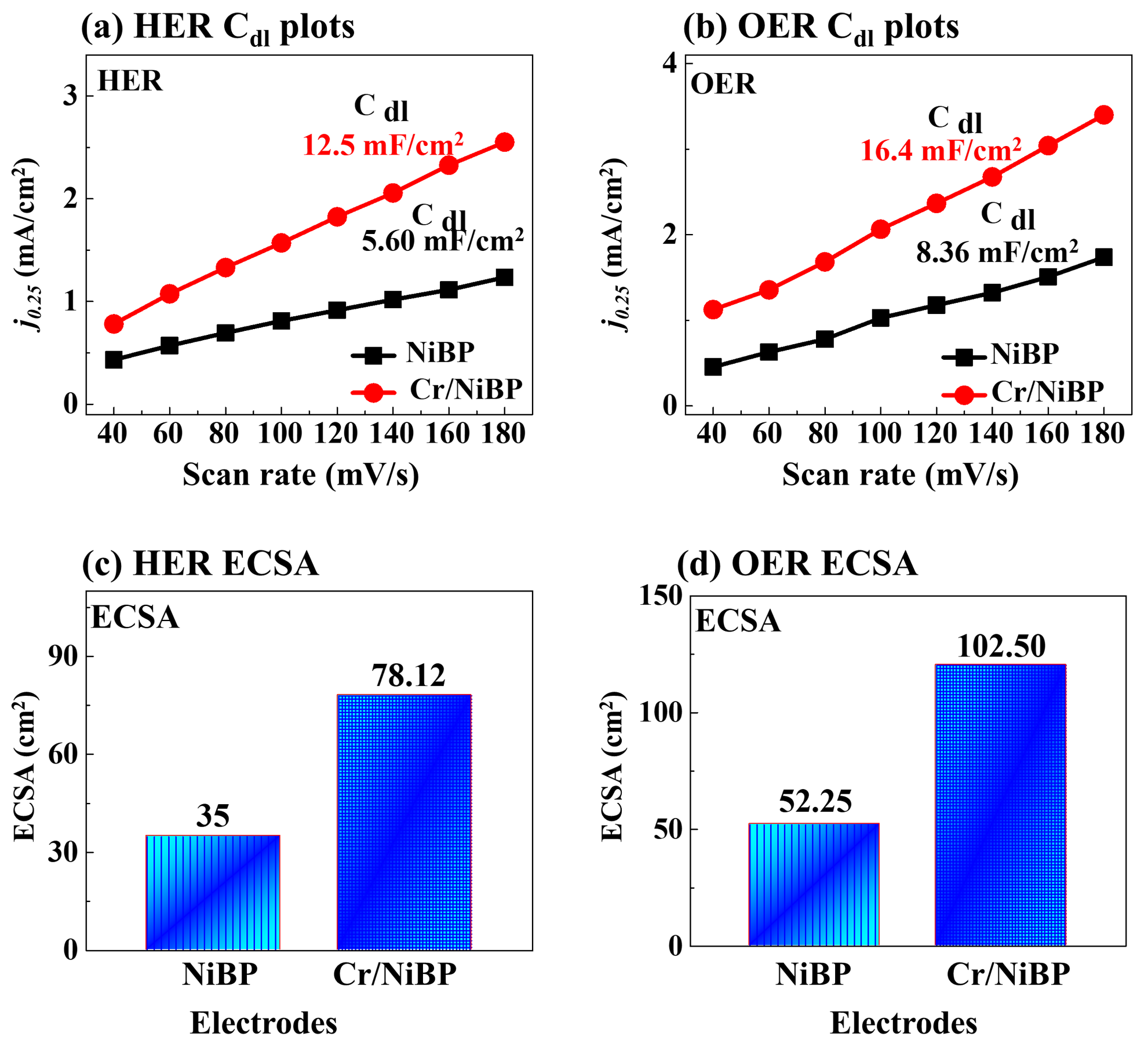


**Figure S33.** (a) & (b) HER and OER double layer capacitance (C_dl_) value comparison between bare NiBP and Cr/NiBP. (c) & (d) HER & OER electrochemical surface area (ECSA) comparison. A more detailed discussion can be found in SI text S-1.13. Cr doping effect.

**S-2.4.5. NiBP and Cr/NiBP comparison: HER & OER**


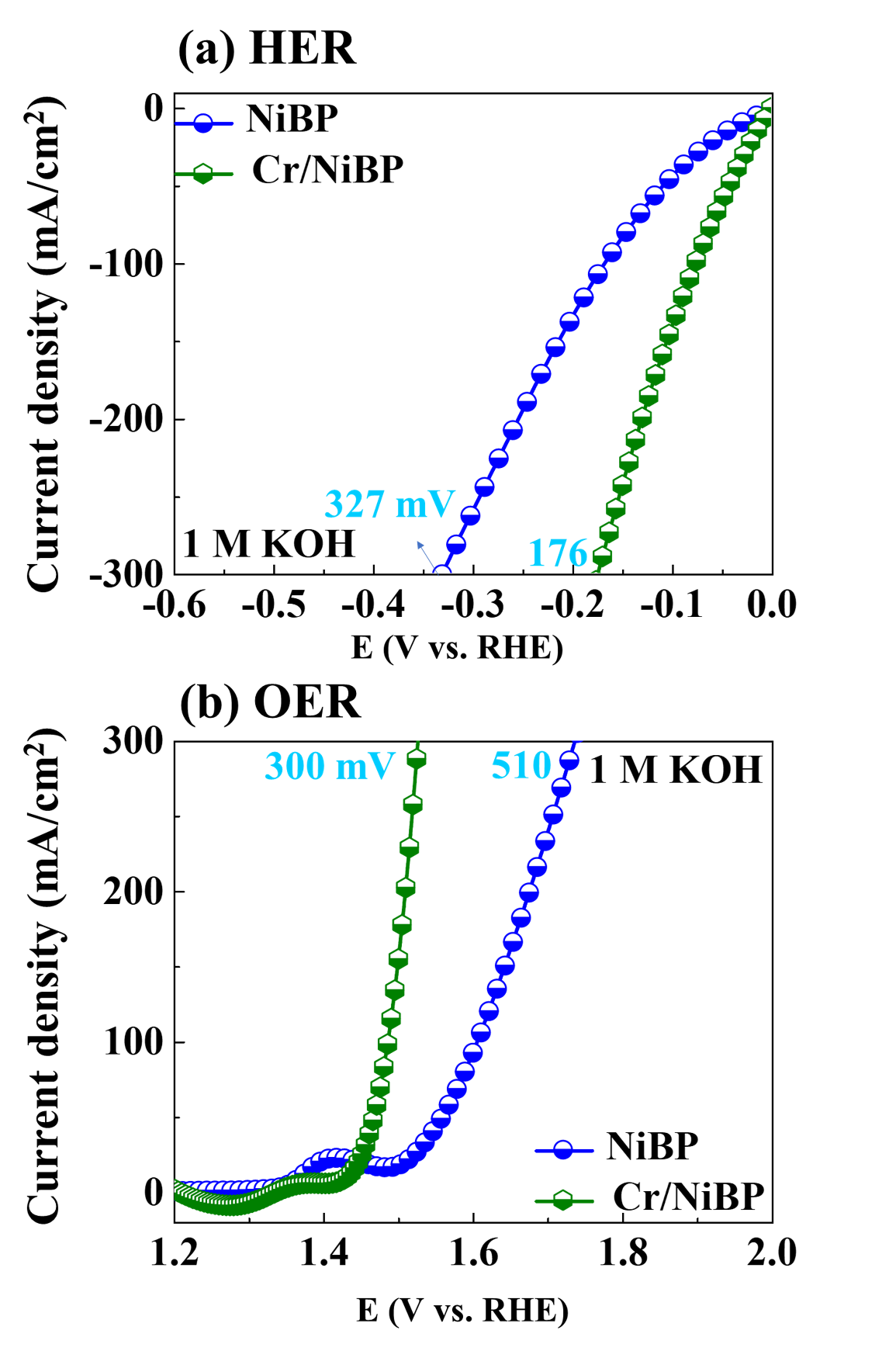


**Figure S34.** (a) & (b) HER and OER LSV comparison of bare NiBP and Cr/NiBP in 1 M KOH. After Cr doping, both HER and OER performances significantly improved. A more detailed discussion can be found in SI text S-1.13. Cr doping effect.

**S-2.4.6. NiBP and Cr/NiBP comparison:** **ECSA-normalized LSV**


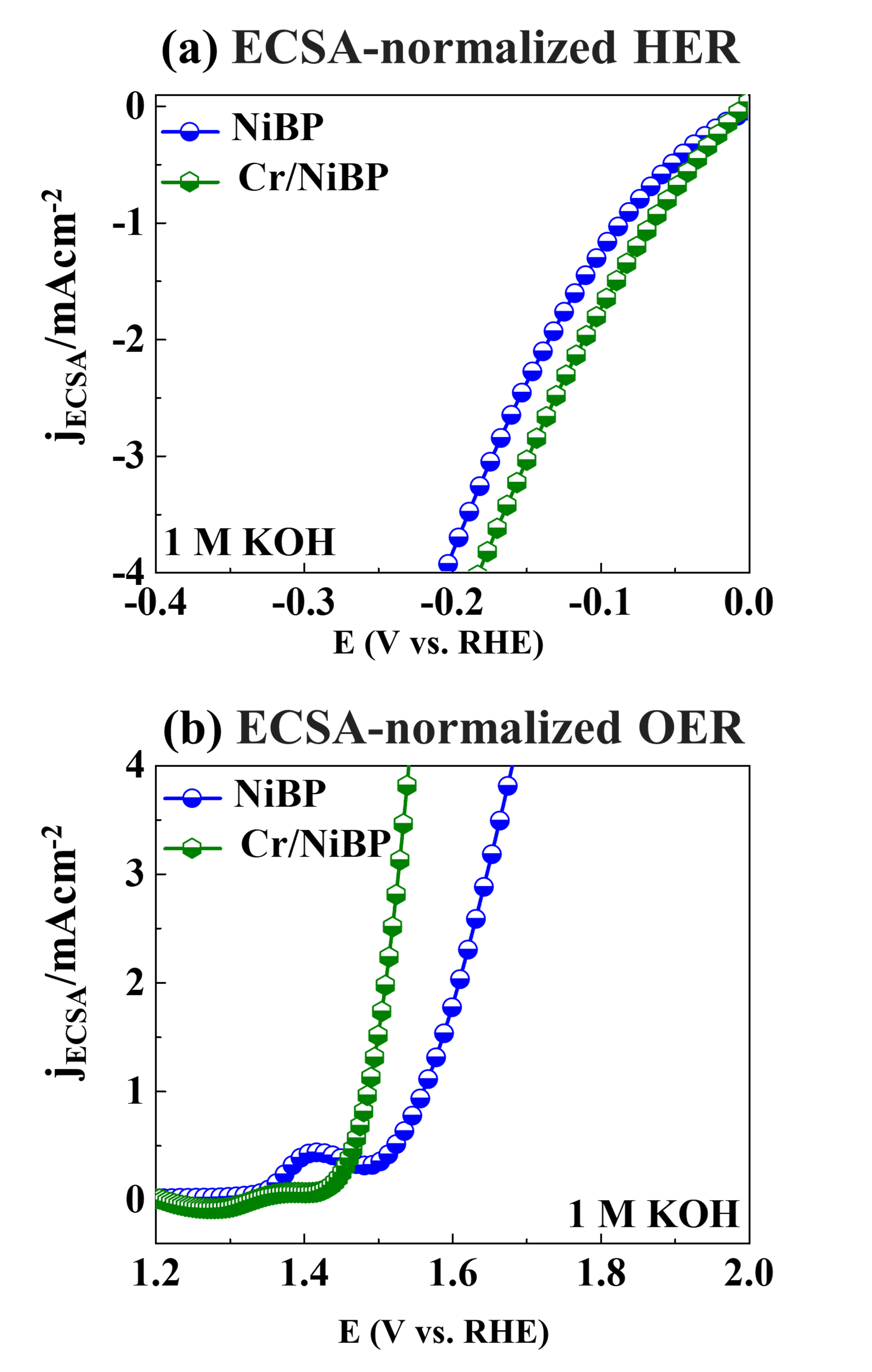


**Figure S35.** (a) & (b) HER and OER ECSA-normalized LSV curves comparison of bare NiBP and Cr/NiBP in 1 M KOH. The normalized HER and OER of Cr/NiBP still exhibited much better intrinsic activity, indicating successful Cr doping. A more detailed discussion can be found in SI text S-1.13. Cr doping effect.

**S-3. Analysis on Cr/NiBP electrode**

**S-3.1. Structural analysis on Cr/NiBP**

**S-3.1.1. CV in OER: oxidation peak formation**


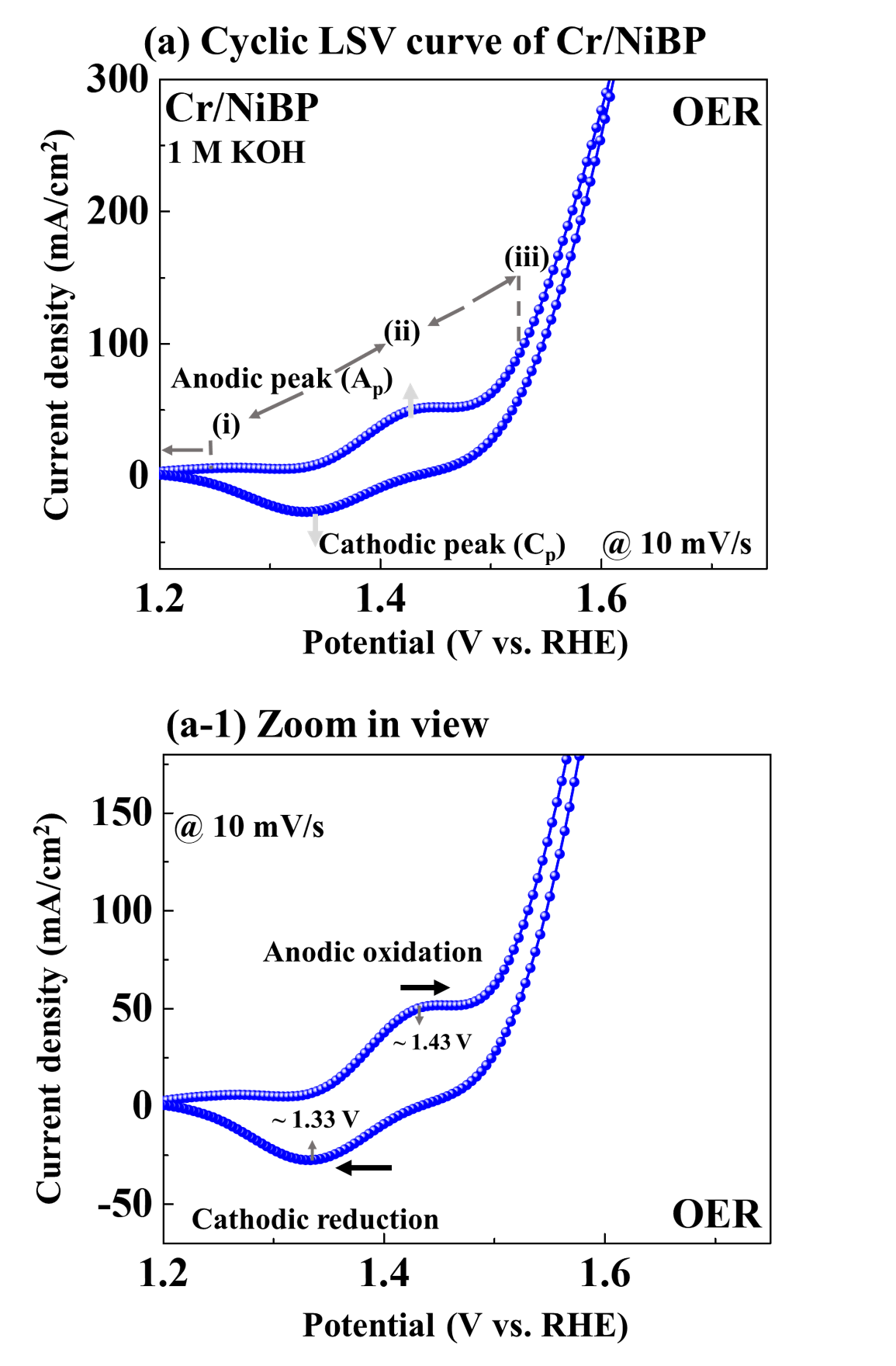


**Figure S36.** CV in the OER LSV range of Cr/NiBP electrode at the scan rate of 10 mVs^-1^ in 1 M KOH. (a) OER LSV curves. (a-1) Corresponding zoom-in view. A more detailed discussion can be found in SI text S-1.10.

**S-3.1.2. TEM analysis on Cr/NiBP**


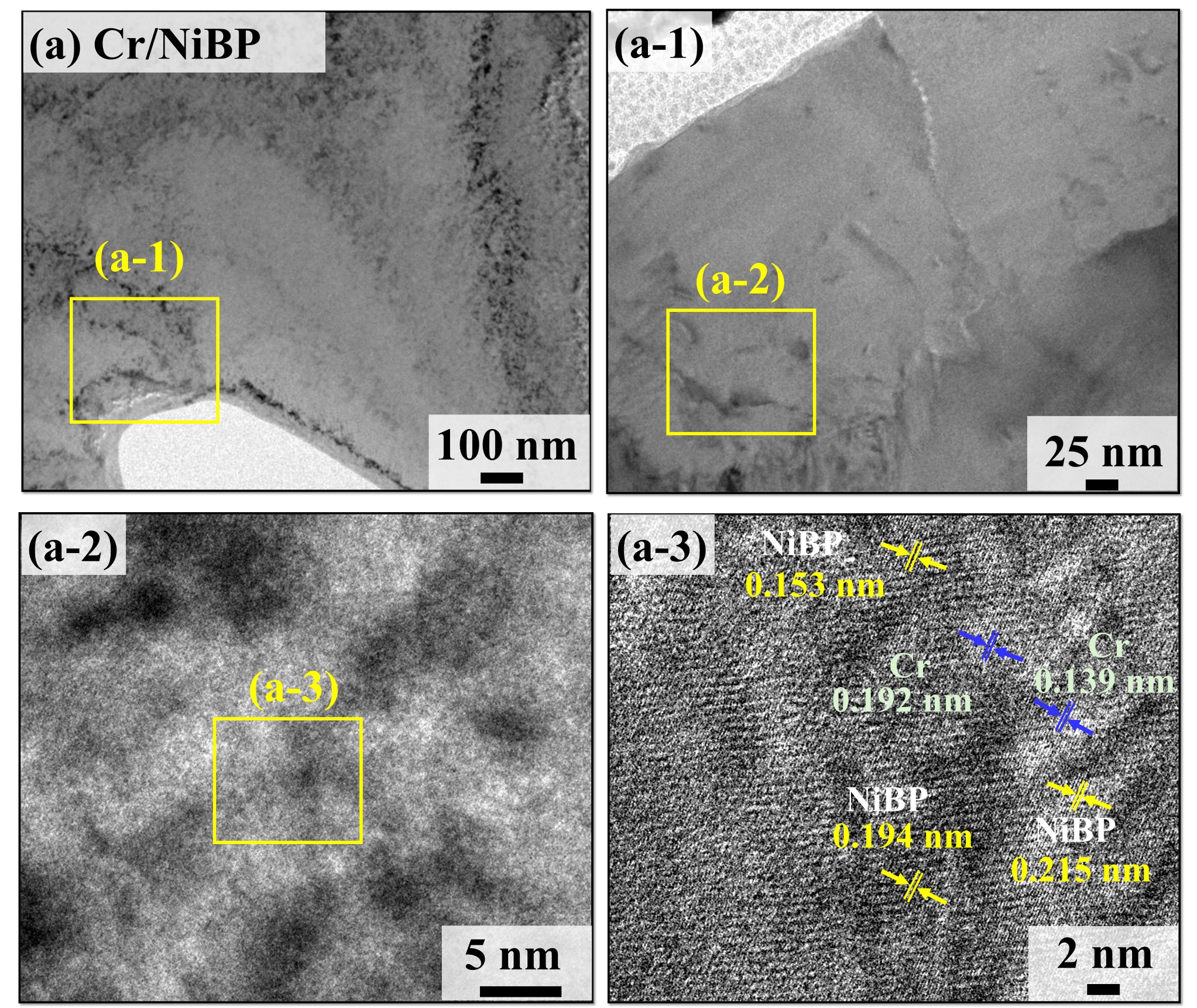


**Figure S37.** TEM analysis on Cr/NiBP micro spherical electrode. (a) FIB processed cross-sectional area. (a-1) – (a-2) Magnified views. (a-3) High-resolution TEM image with lattice distance. The lattice distance of 0.204 and 0.139 nm can be assigned to the Cr nanocrystal distribution. The interplanar distances 0.153, 0.206 and 0.321 nm can correspond to NiBP. This clearly indicated that the Cr- was incorporated in the NiBP matrix in a nano-cluster phase. Overall, the Cr/NiBP electrocatalyst demonstrated a polycrystal structure.

**S-3.1.3. EDS analysis on Cr/NiBP**


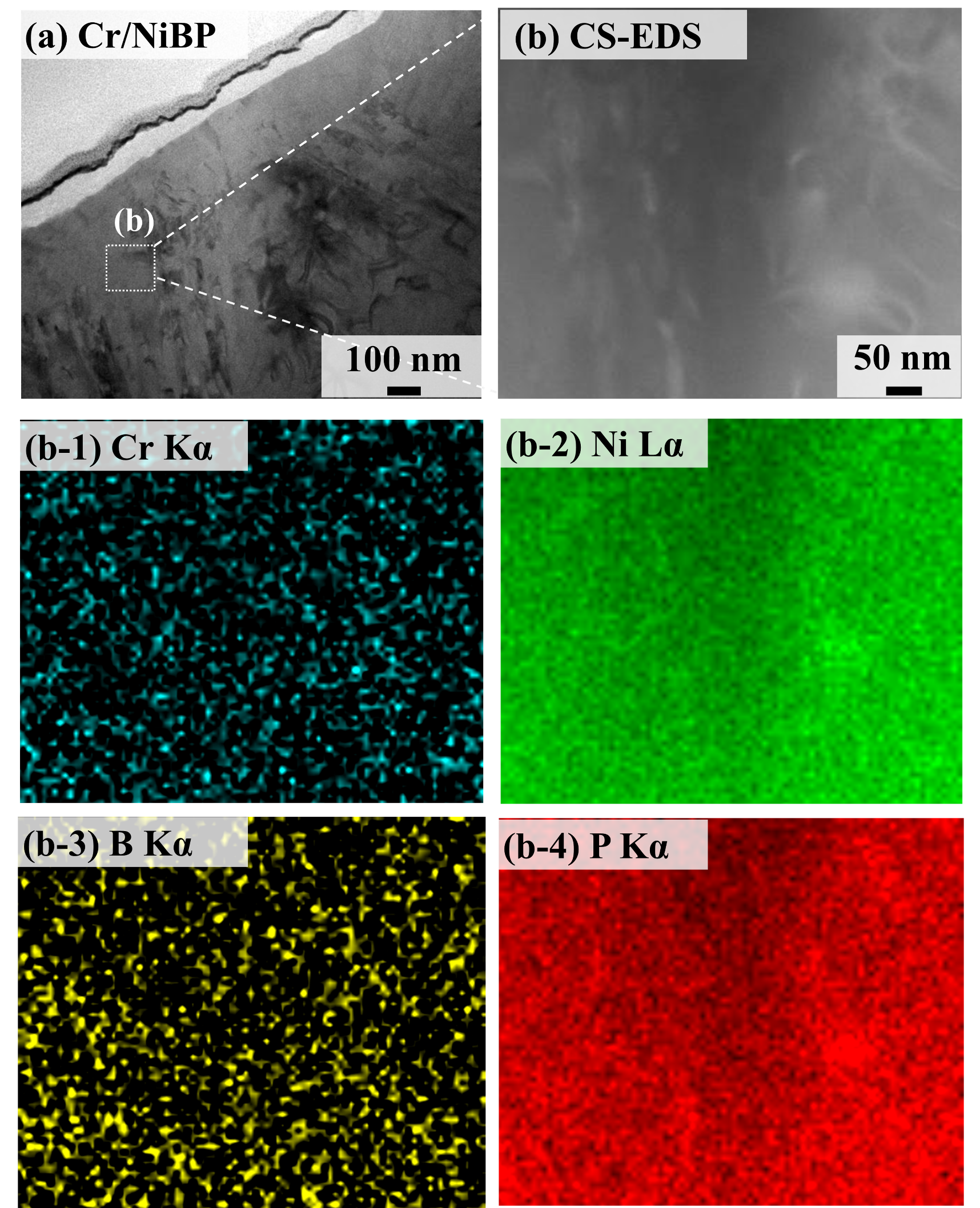


**Figure S38.** (a) – (b) TEM image with an enlarged view. (b-1) – (b-4) Cross-sectional (CS) EDS maps for Cr/NiBP electrocatalyst. HRTEM EDS elemental side-view phase maps confirm the appearance of Cr, Ni, B, and P in the selected cross-sectional area.

**S-3.1.4. XRD pattern analysis on Cr/NiBP**


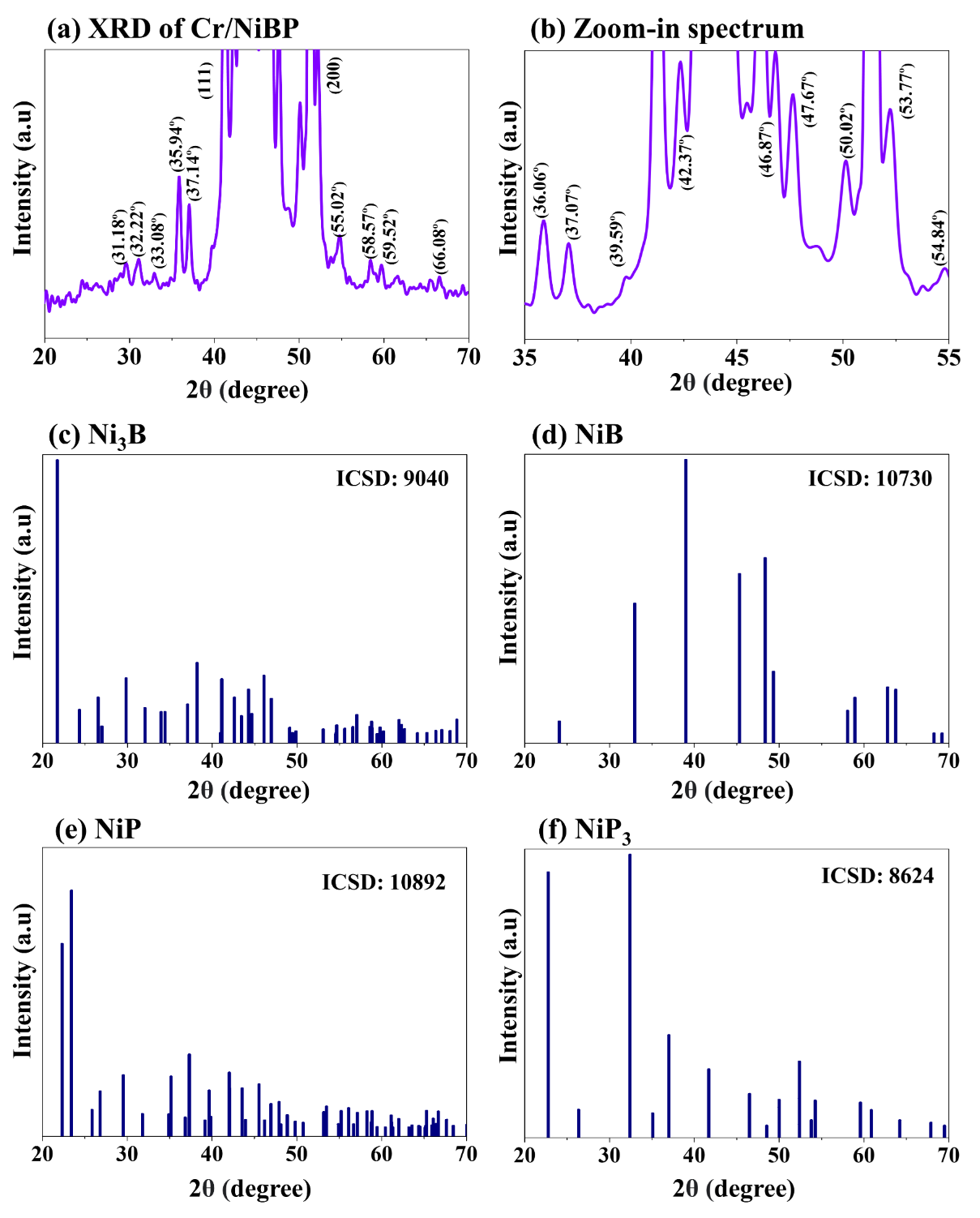


**Figure S39.** X-ray diffraction (XRD) patterns of Cr/NiBP and simulated XRD patterns of related materials. (a) XRD pattern Cr/NiBP electrode. (b) Zoom-in view between 35 and 55^o^. (c) – (f) Simulated XRD diffraction patterns of Ni_3_B, NiB, NiP and NiP_3_ phase. Simulated XRD patterns of different material systems can be found at <http://icsd.kisti.re.kr>. More details about X-ray diffraction analysis (XRD) analysis can be found in SI text S-1.8. X-ray diffraction (XRD) analysis.

**S-3.2. 3-E electrochemical analysis on Cr/NiBP**

**S-3.2.1. TOF of Cr/NiBP in different pH solutions**

**
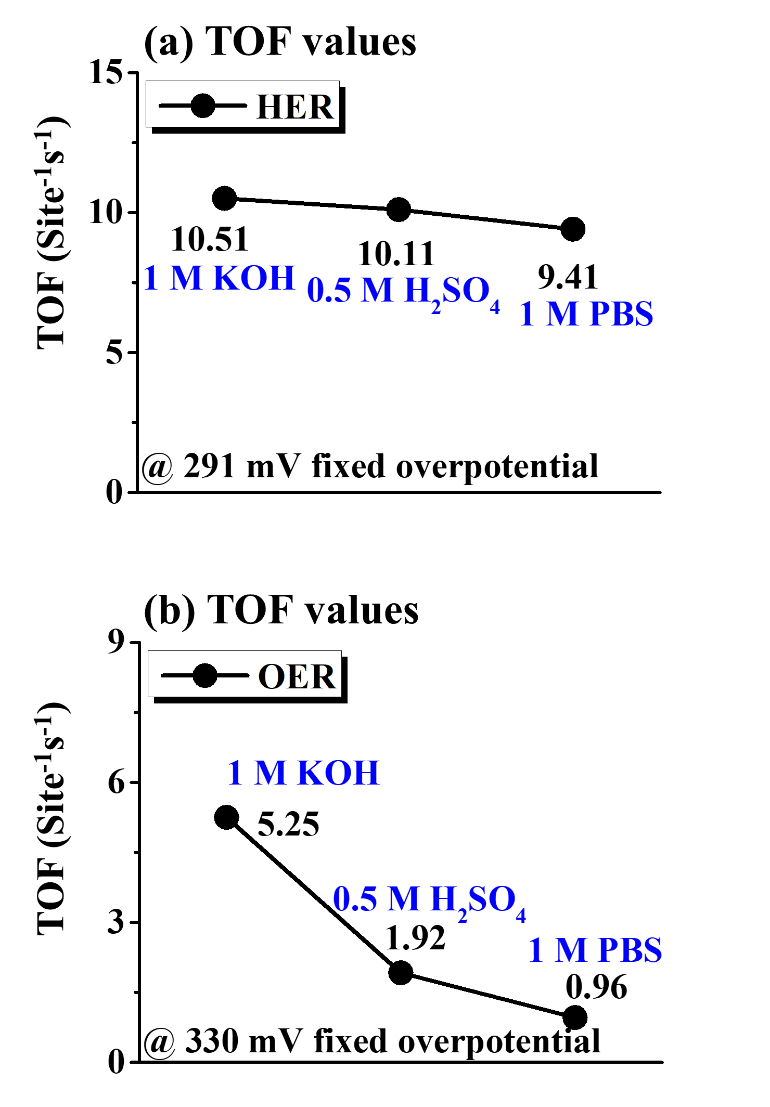
**

**Figure S40.** (a) & (b) HER and OER TOF values of Cr/NiBP MS electrocatalysts in different electrolytes. The TOF values are calculated based on LSV plots in different electrolytes and 600 mA/cm^2^ corresponding fixed overpotential in Figs. 4(d) – 4(i). A more detailed discussion about TOF calculation can be found in SI text S-1.11. TOF calculation of Cr/NiBP.

**S-3.2.2. HER/OER CA performance & LSV comparison of Cr/NiBP**


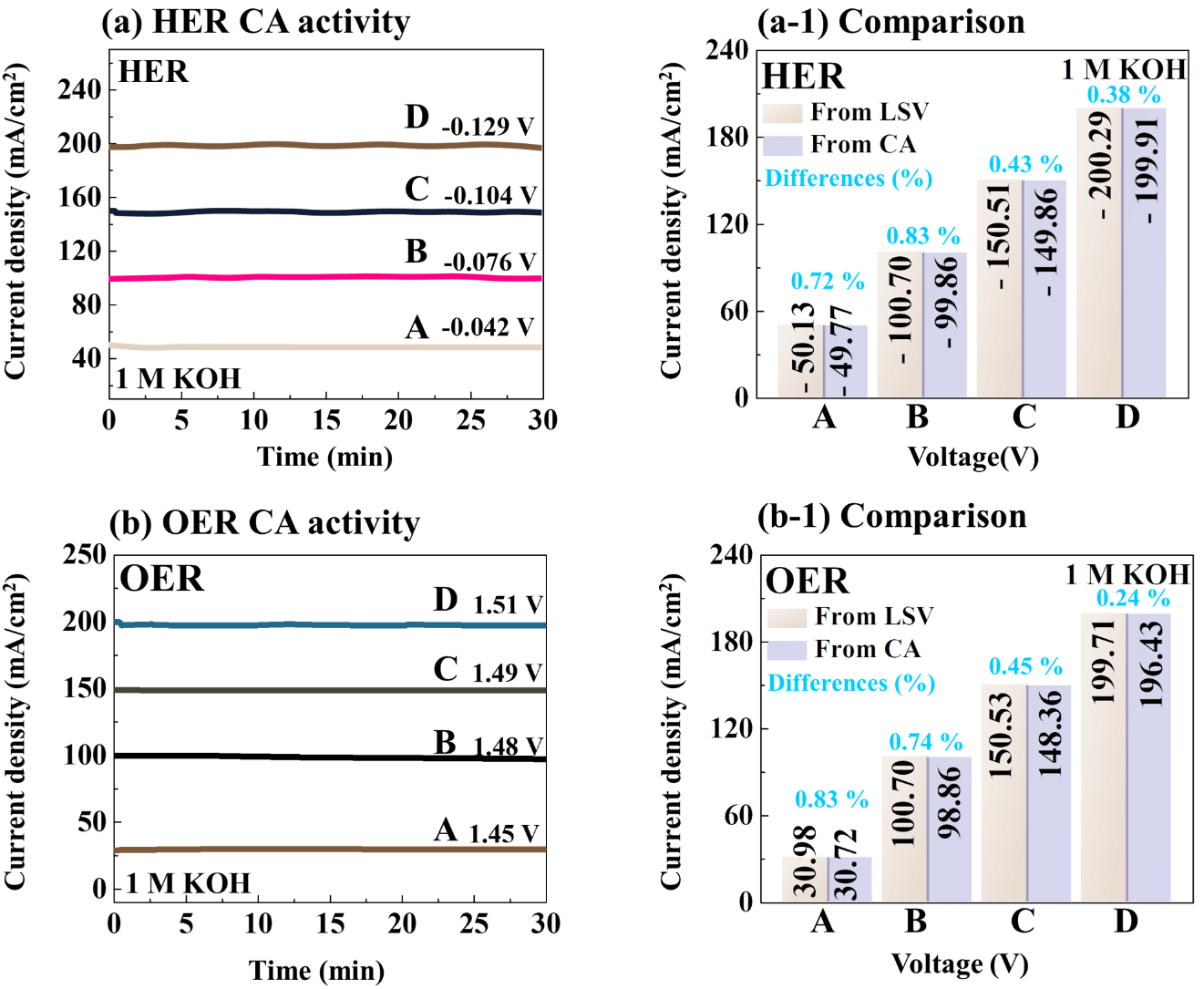


**Figure S41.** (a) – (b) HER/OER Chronoamperometry **(**CA) activities with various applied potentials and LSV performance comparison of Cr/NiBP MS electrocatalyst in 1 M KOH. HER/OER CA. (a-1) – (b-1) CA/LSV current comparison.

**S-3.2.3. HER/OER repeatability test of Cr/NiBP**


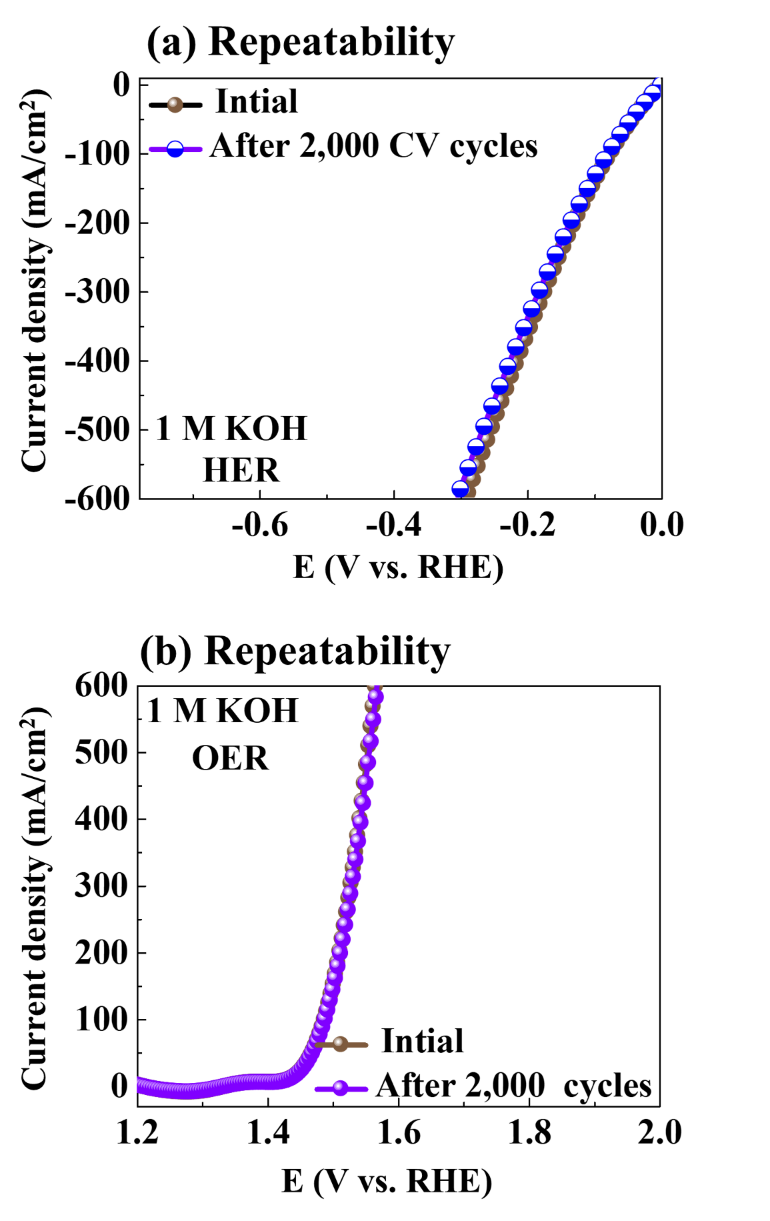


**Figure S42.** (a) & (b) HER and OER repeatability of Cr/NiBP MS electrocatalyst before/after 2,000 cycle CV (scan rate of 100 mV/s for 20 hours) operations.

**S-3.2.4. HER/OER stability test of Cr/NiBP**


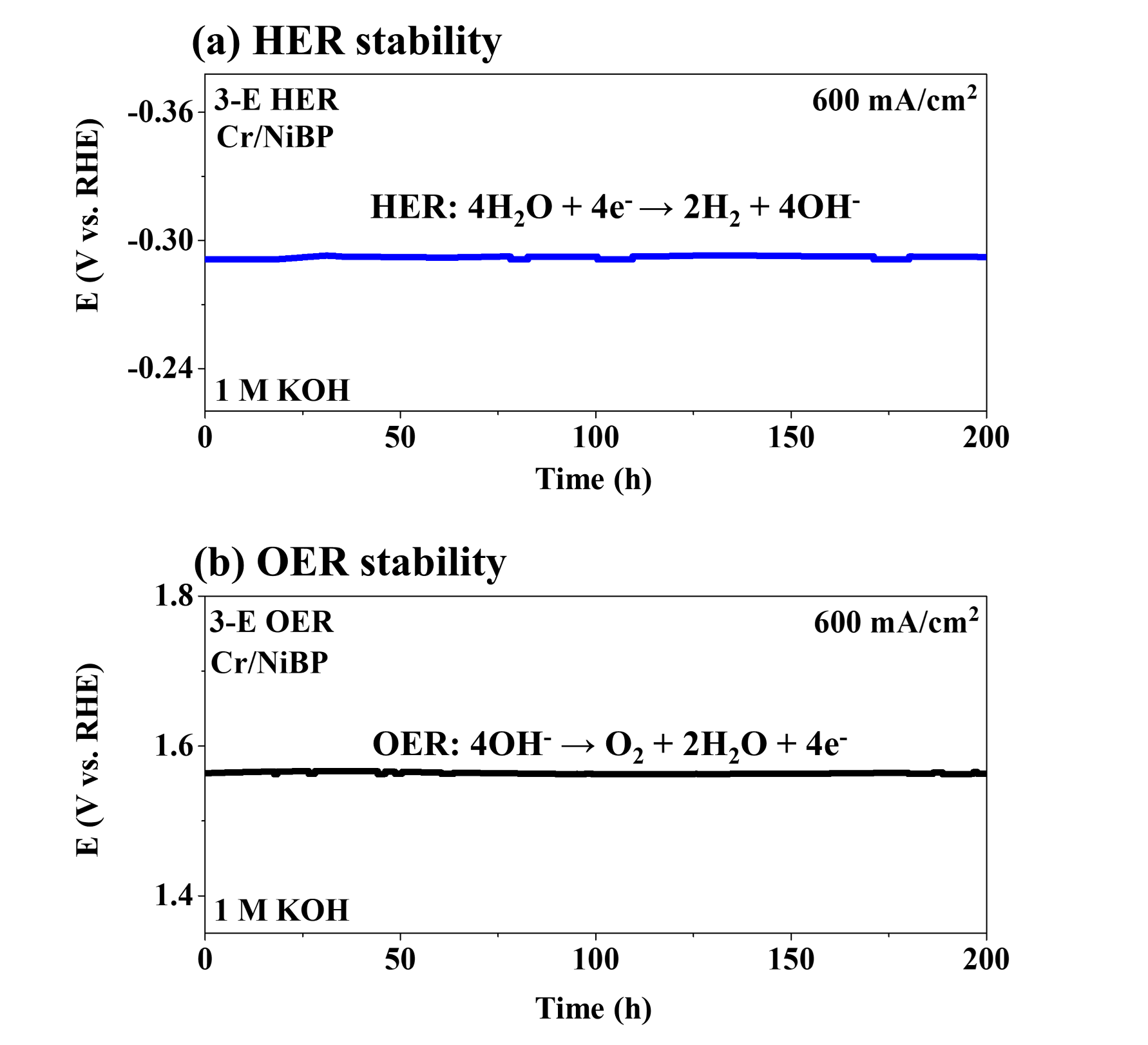


**Figure S43.** HER/OER stability test of Cr/NiBP micro spheres in 1 M KOH. (a) & (b) HER/OER 3-E stability test at 600 mA/cm^2^ in 1 M KOH for 200 hours (Over 8 days operation). Cr/NiBP micro spheres clearly demonstrated long-term stability at high currents in alkaline media.

**S-3.2.5. HER/OER comparison of Cr/NiBP & NF**


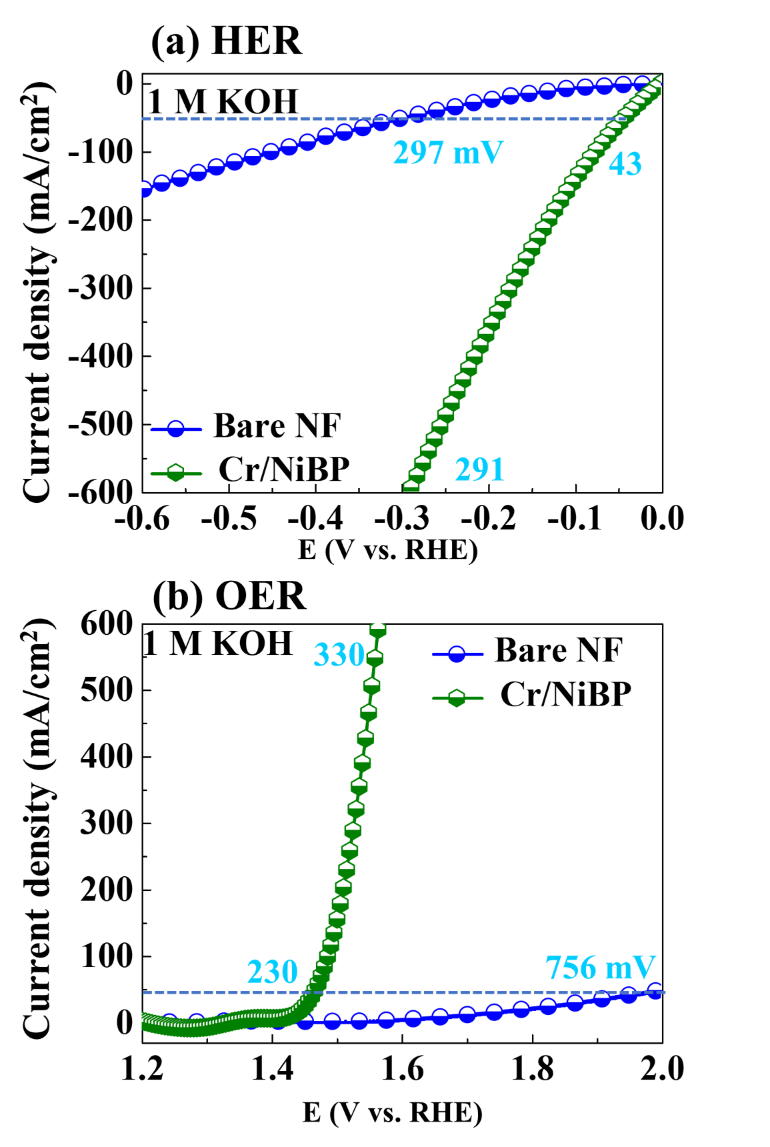


**Figure S44.** HER/OER performance of bare Ni foam (NF) substrate and Cr/NiBP MS electrocatalyst. (a) & (b) HER & OER LSV polarization curves in 1 M KOH. After the fabrication of Cr/NiBP electrocatalysts, the HER and OER performance significantly improved as compared to bare NF. A more detailed discussion can be found in SI text S-1.13. Cr doping effect.

**S-3.2.6. Water–gas displacement for faradic efficiency**


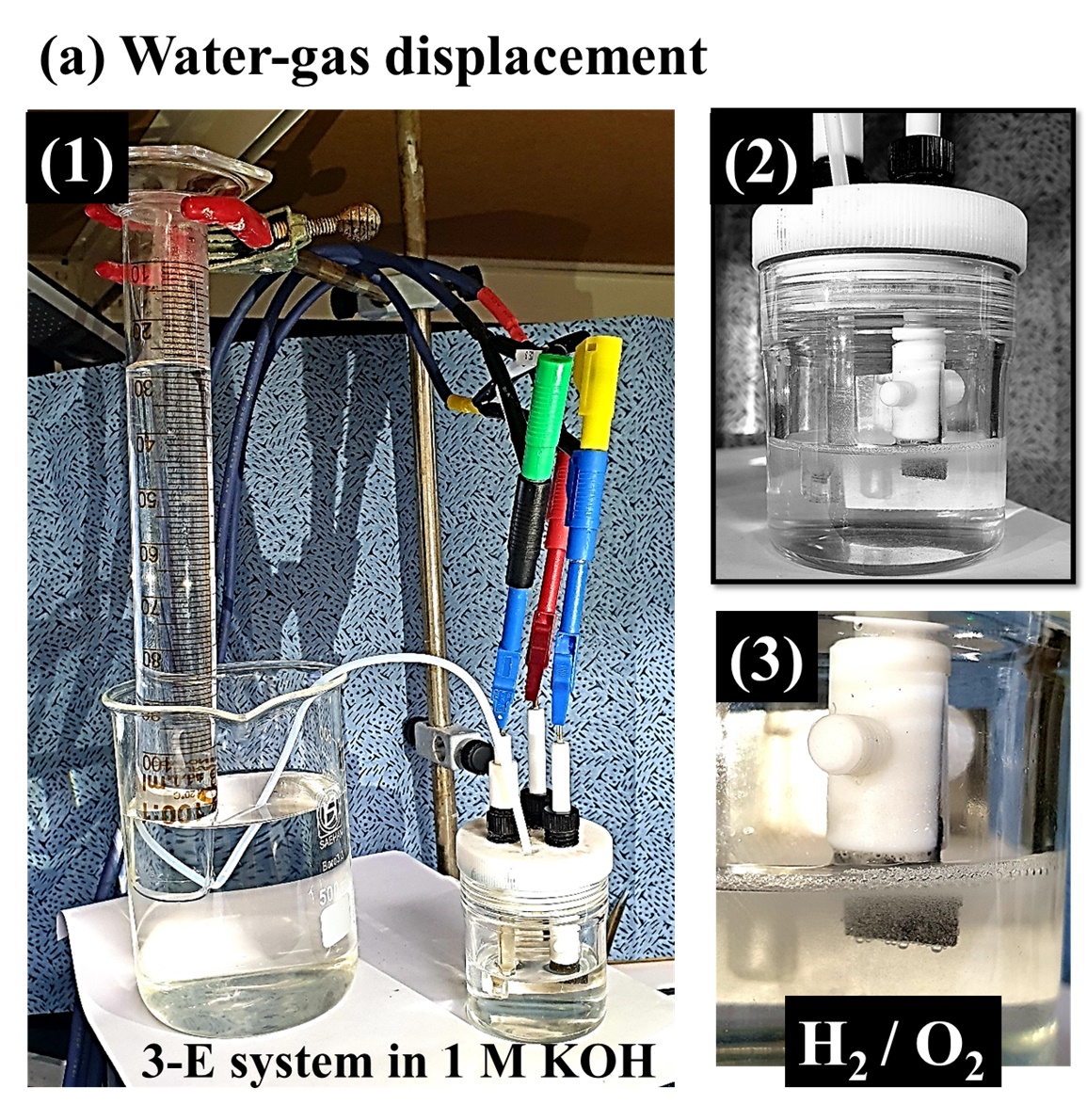


**Figure S45.** (a) Digital images of water–gas displacement and gas bubble formation in 1 M KOH for Faradaic efficiency measurements of Cr/NiBP MS electrocatalyst.

**S-3.2.7. Faradaic efficiency of Cr/NiBP electrode**

**
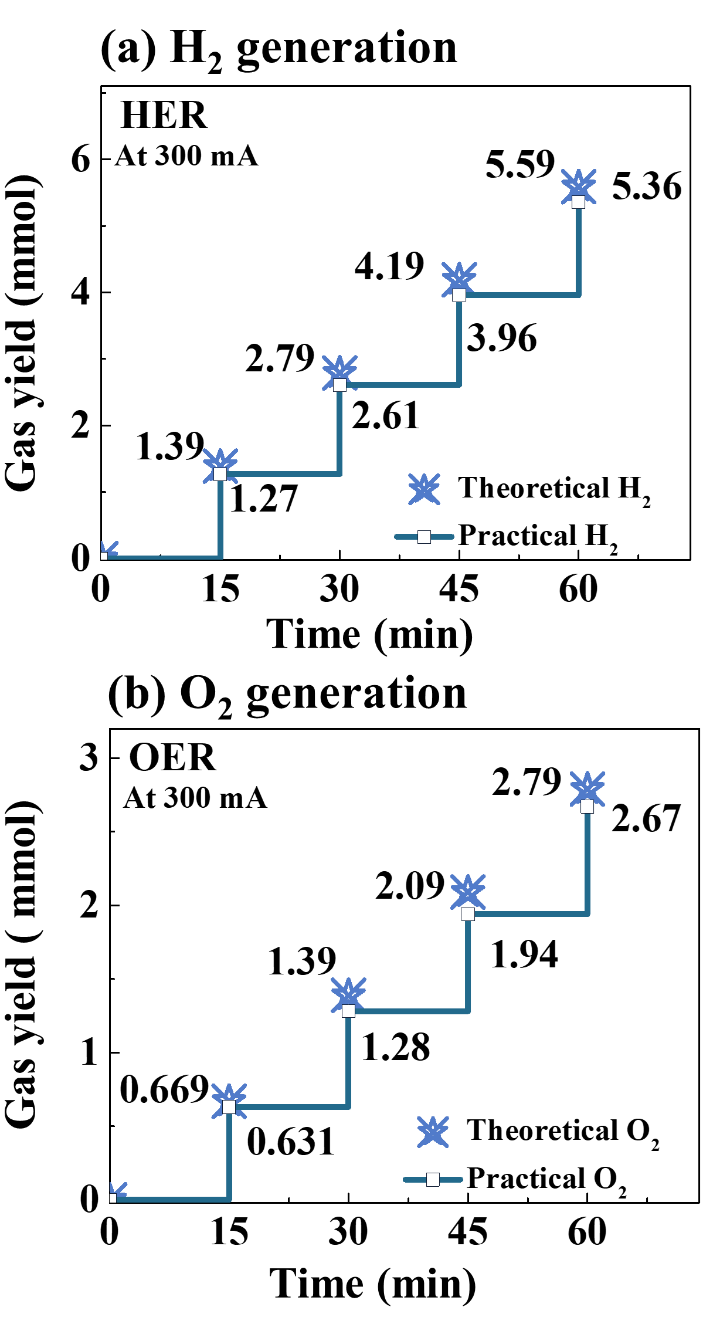
**

**Figure S46.** Faradaic efficiency of Cr/NiBP MS electrocatalyst. (a) & (b) Actual and theoretical H_2_ and O_2_ generation for 15 ~ 60 min observation in 1 M KOH. A more detailed discussion about calculation can be found in SI text S-1.12. Faradaic efficiency calculation.

**S-3.3. 2-E electrochemical analysis on bifunctional Cr/NiBP ǁ Cr/NiBP**

**S-3.3.1. 2-E comparison on Cr/NiBP and NiBP**


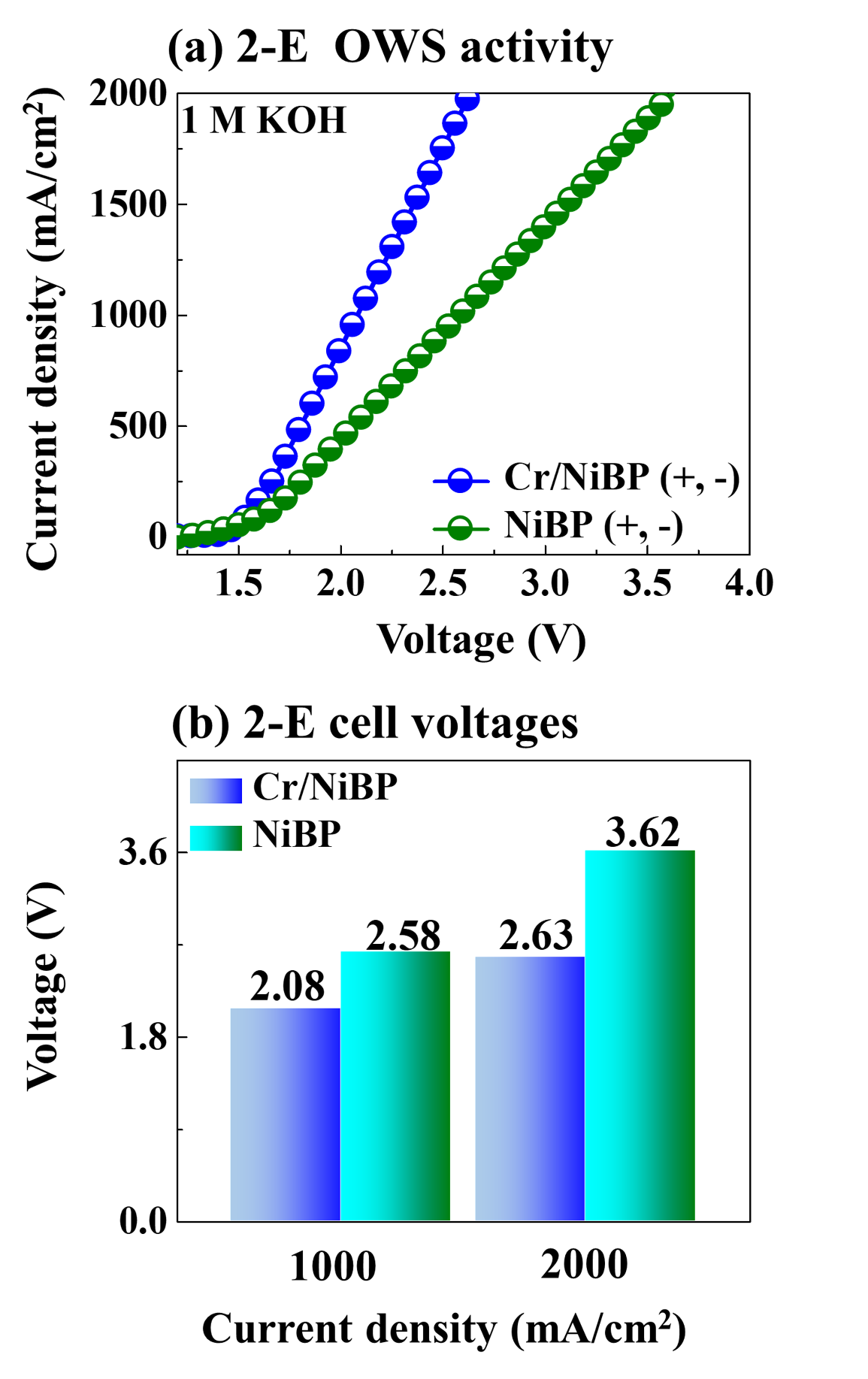


**Figure S47.** 2-E OWS performance comparisons of bifunctional Cr/NiBP and NiBP electrodes in 1 M KOH. (a) 2-E high current LSV curves in 1 M KOH. (b) Summarized cell voltage at 1,000 and 2,000 mA/cm^2^. A more detailed discussion can be found in SI text S-1.13. Cr doping effect.

**S-3.3.2. 2-E CA and LSV comparison on Cr/NiBP**


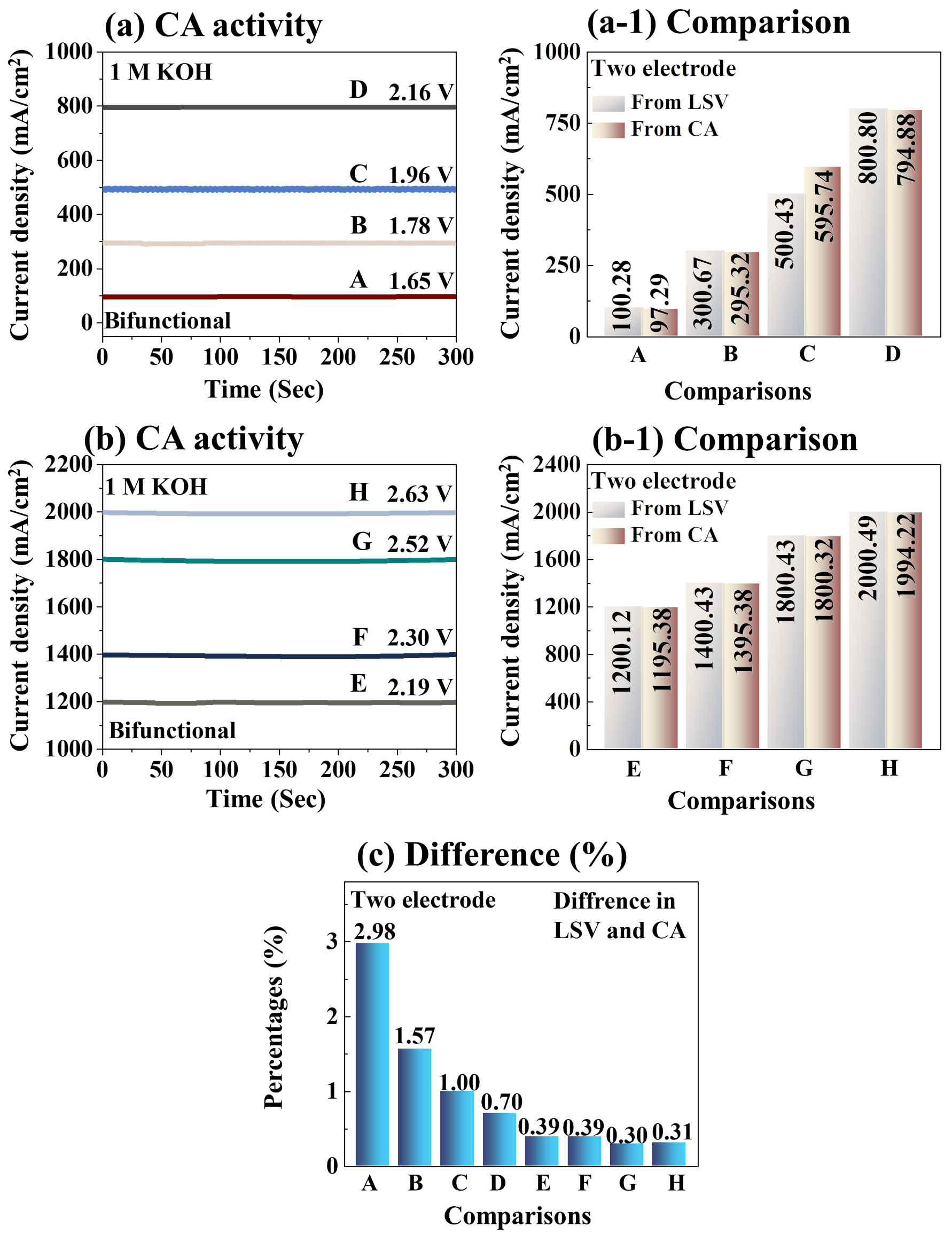


**Figure S48.** (a) – (b) 2-E steady**-**state CA observation of bifunctional Cr/NiBP MS electrocatalysts. (a-1) – (b-1) Comparison of obtained current density between LSV and CA. (c) Current difference in percentages ( %).

**S-3.3.3. 2-E repeatability test of bifunctional Cr/NiBP**


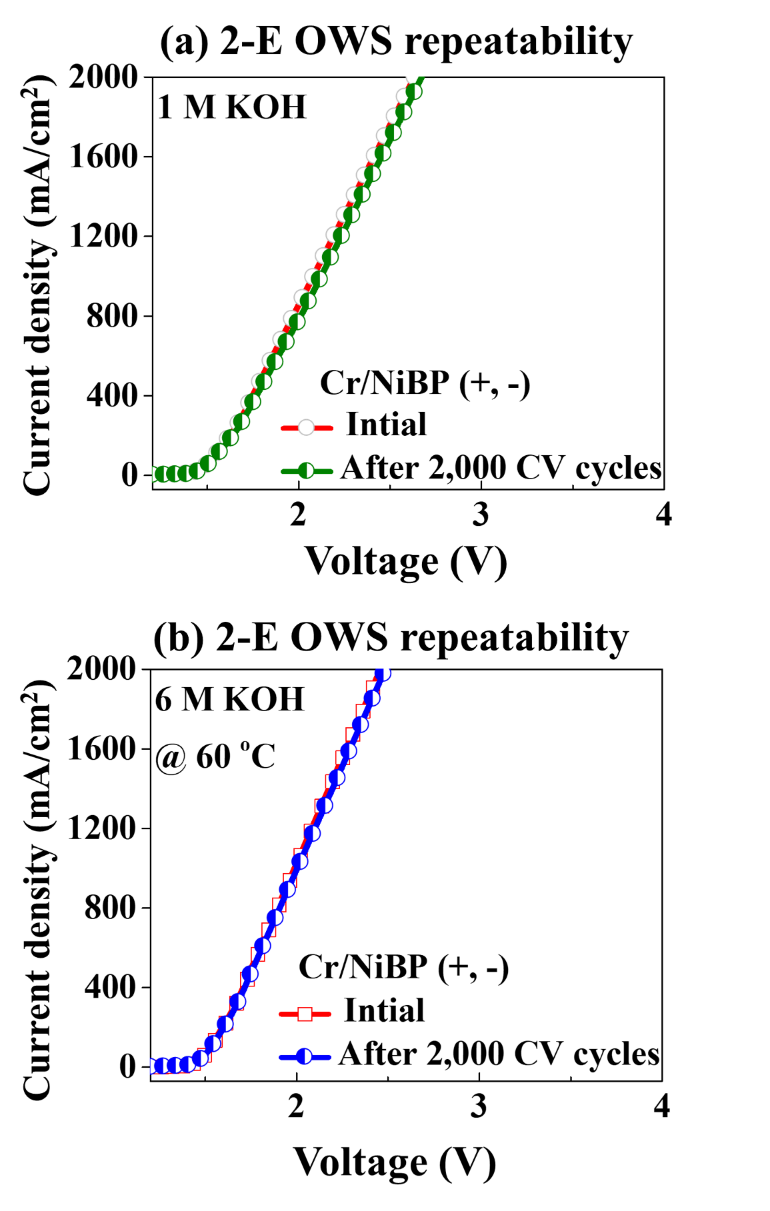


**Figure S49.** (a) & (b) 2-E overall water splitting (OWS) repeatability test of the bifunctional Cr/NiBP MS electrocatalysts after 2,000 cycles CV (scan rate at 100 mV/s) of ~ 20-hour operation in 1 M and 6 M KOH.

**S-3.3.4. 2-E stability at industrial condition**

**
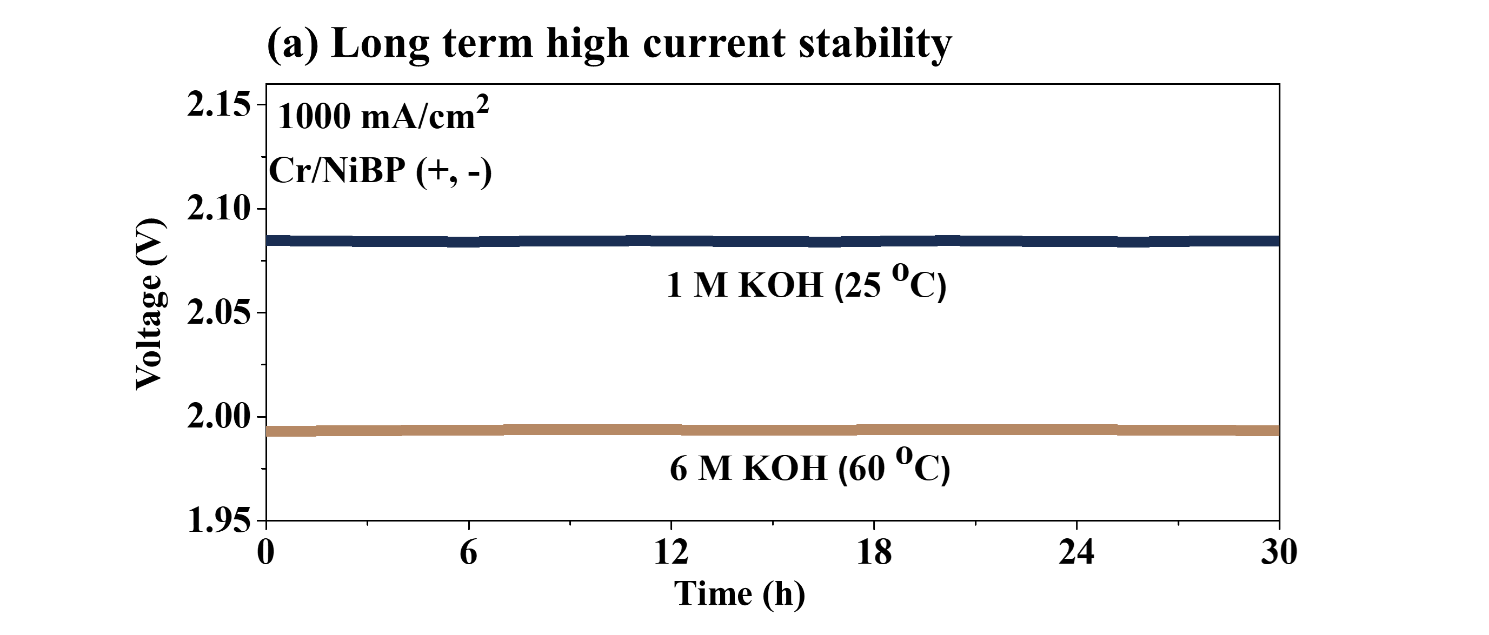
**

**Figure S50.** (a) Bifunctional stability of Cr/NiBP MS electrocatalysts at 1,000 mA/cm^2^ in 1 M KOH and in 6 M KOH for 30 hours.

**S-3.3.5. 2-E natural water performance of Cr/NiBP**

**
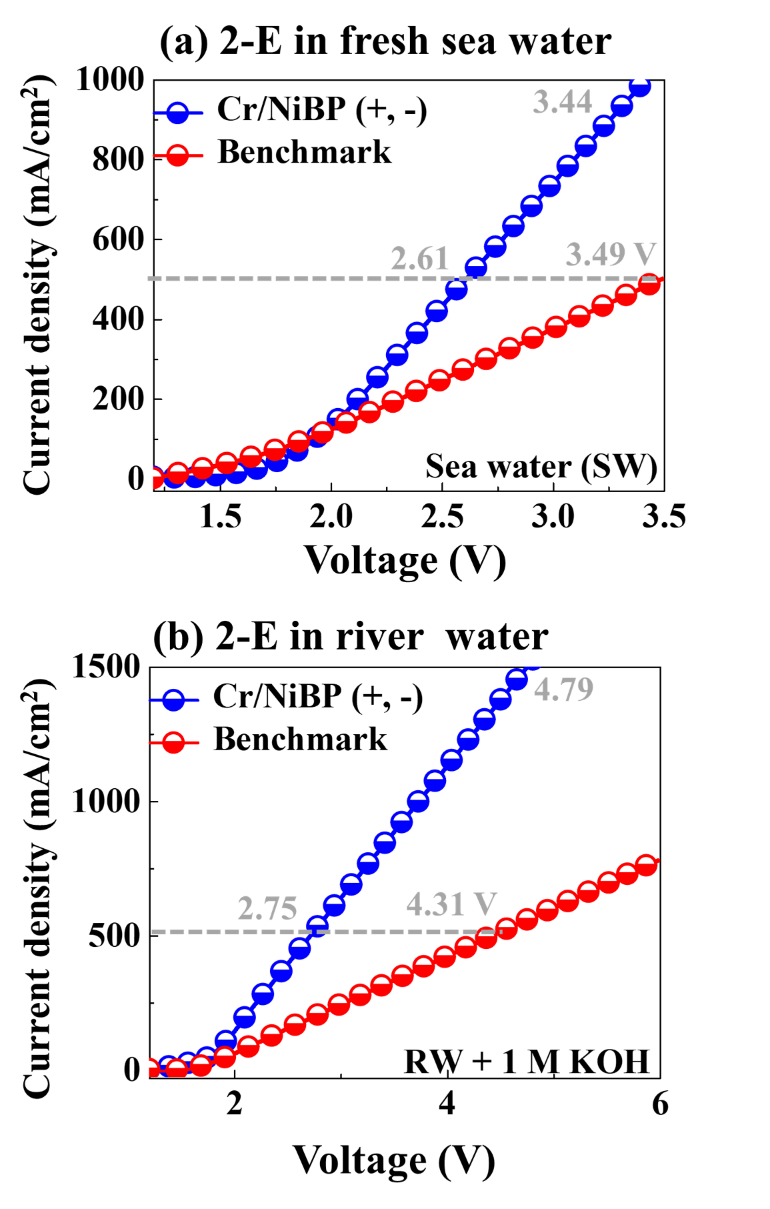
**

**Figure S51.** (a) & (b) Bifunctional Cr/NiBP (+, -) OWS performance comparisons with the benchmarks in natural seawater (SW) and alkaline river water (RW + 1 M KOH).

**S-3.3.6. 2-E seawater stability of Cr/NiBP**

**
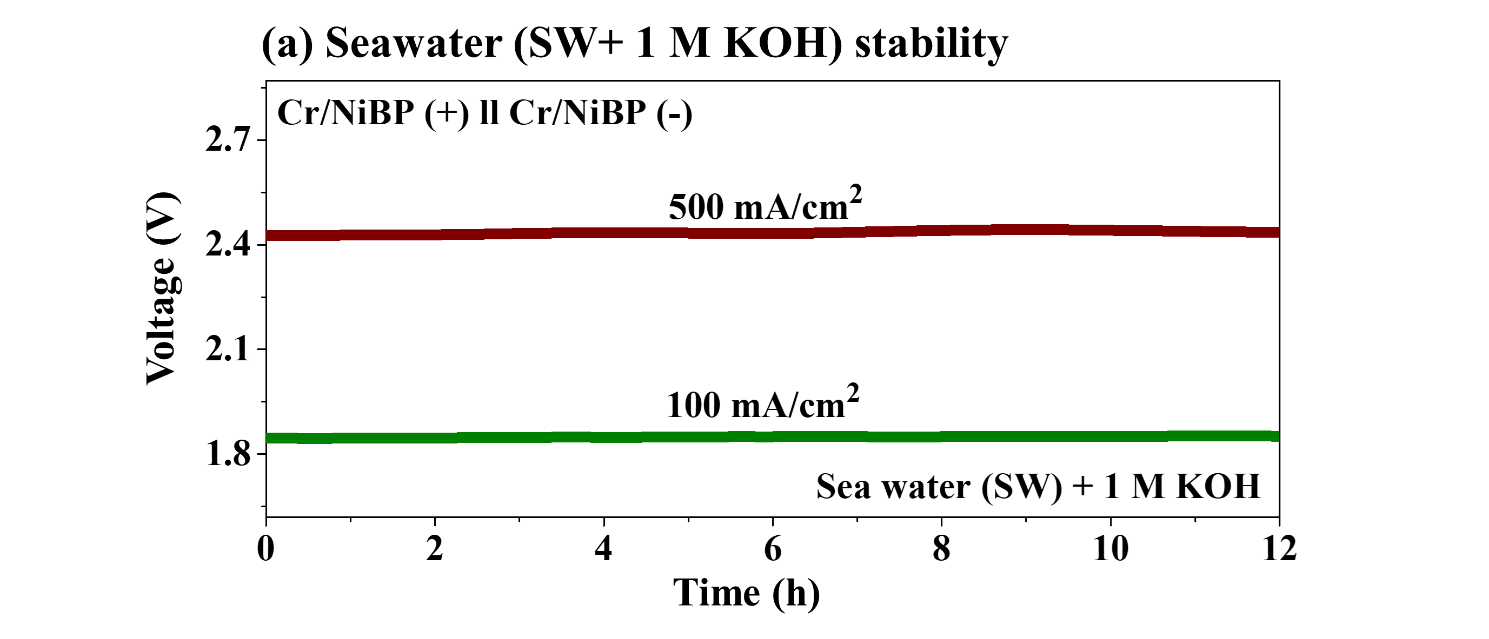
**

**Figure S52.** (a) Dual-step stability tests of bifunctional Cr/NiBP MS electrocatalysts at 100 and 500 mA/cm^2^ in alkaline seawater (SW + 1 M KOH).

**S-3.4. 2-E electrochemical analysis on hybrid (Cr/NiBP ǁ Pt/C) system**

**S-3.4.1. 2-E OWS of hybrid system in different pH**


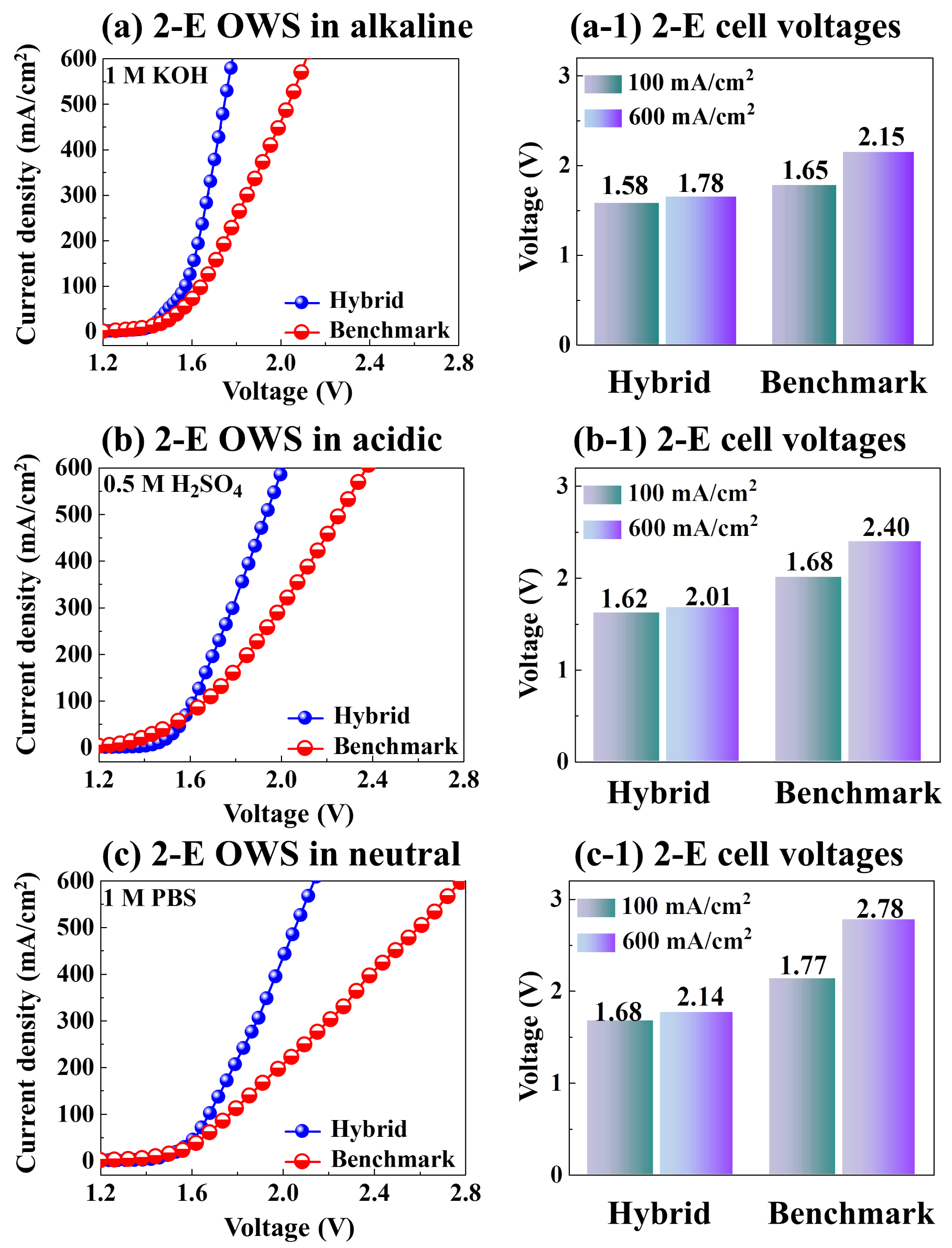


**Figure S53.** (a) – (c) Two-electrode (2-E) OWS performance of hybrid system (Cr/NiBP (+) ǁ Pt/C (-)) as compared with the benchmark system RuO_2_ (+) ‖ Pt/C (-) in 1 M KOH, 0.5 M H_2_SO_4_ and 1 M PBS electrolytes. (a-1) – (c-1) Summarized cell voltage at 100 and 600 mA/cm^2^.

**S-3.4.2. Hybrid CA and LSV comparison**


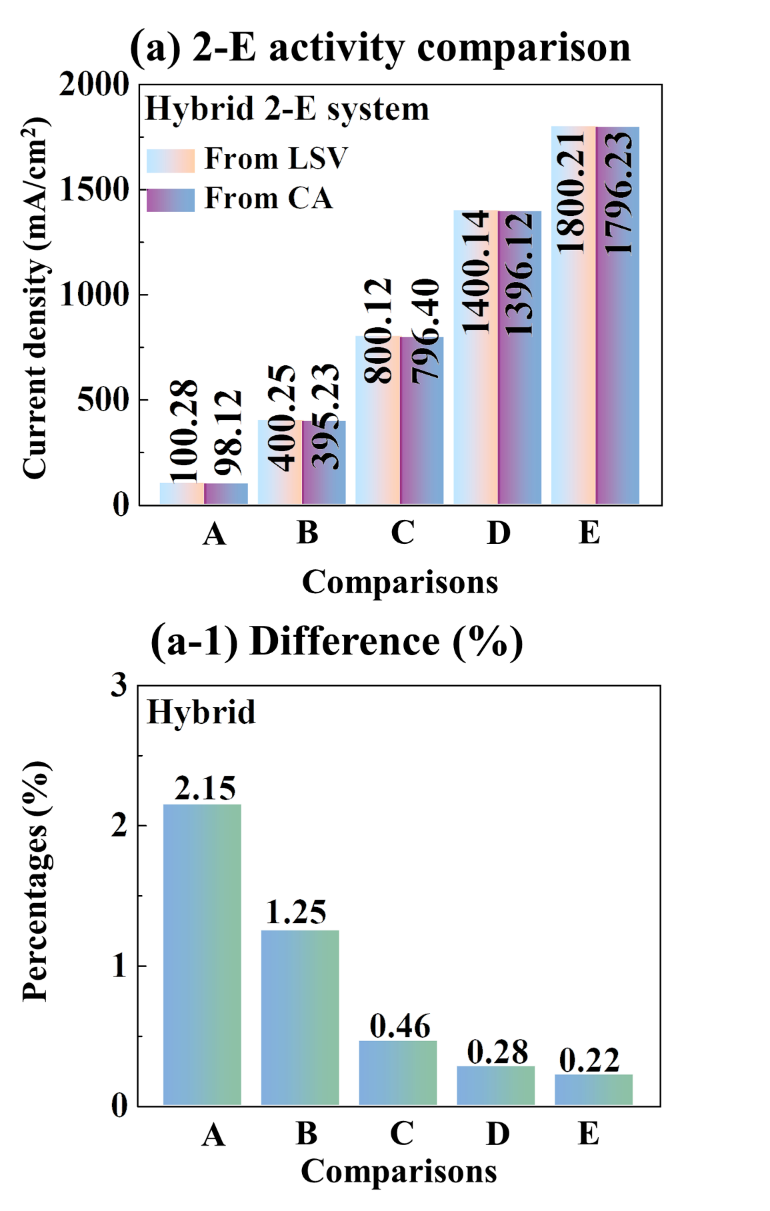


**Figure S54.** 2-E steady**-**state CA and LSV observation of Cr/NiBP hybrid system. (a) Comparison of obtained current density between LSV and CA. (a-1) Current density difference in percentage (%).

**S-3.4.3. Hybrid repeatability in 6 M KOH**


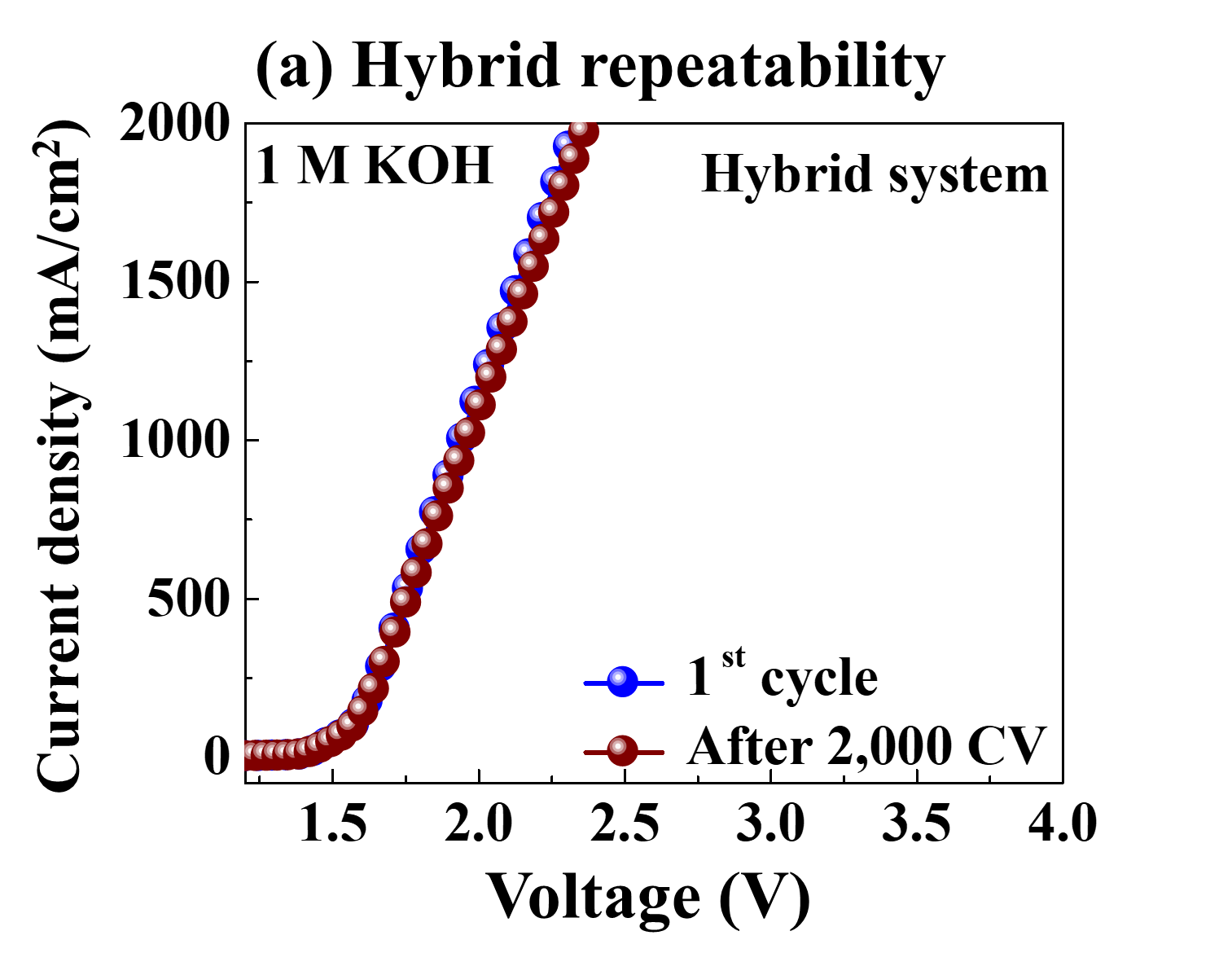


**Figure S55.** (a) 2-E OWS repeatability test of the Cr/NiBP hybrid system before/after 2,000 cycles (over ~ 20 hours) in 1 M KOH.

**S-3.4.4. Hybrid LSV performance in sea and river waters**


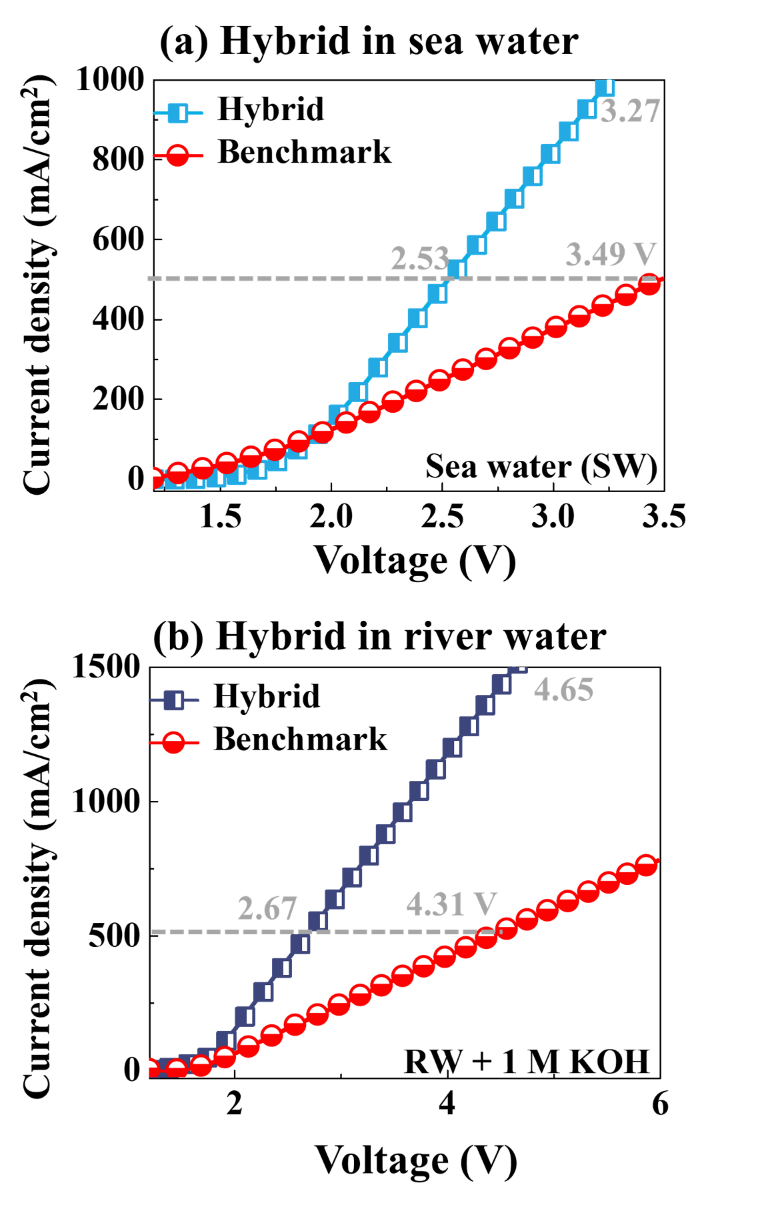


**Figure S56.** 2-E performance of Cr/NiBP hybrid system as compared with benchmark system of RuO_2_ (+) ‖ Pt/C (-) in natural waters. (a) & (b) 2-E OWS LSV performance in natural seawater (SW) and alkaline river water (RW + 1 M KOH).

**S-3.5. Before/after stability analysis on Cr/NiBP**

**S-3.5.1. Before/post stability test: SEM**


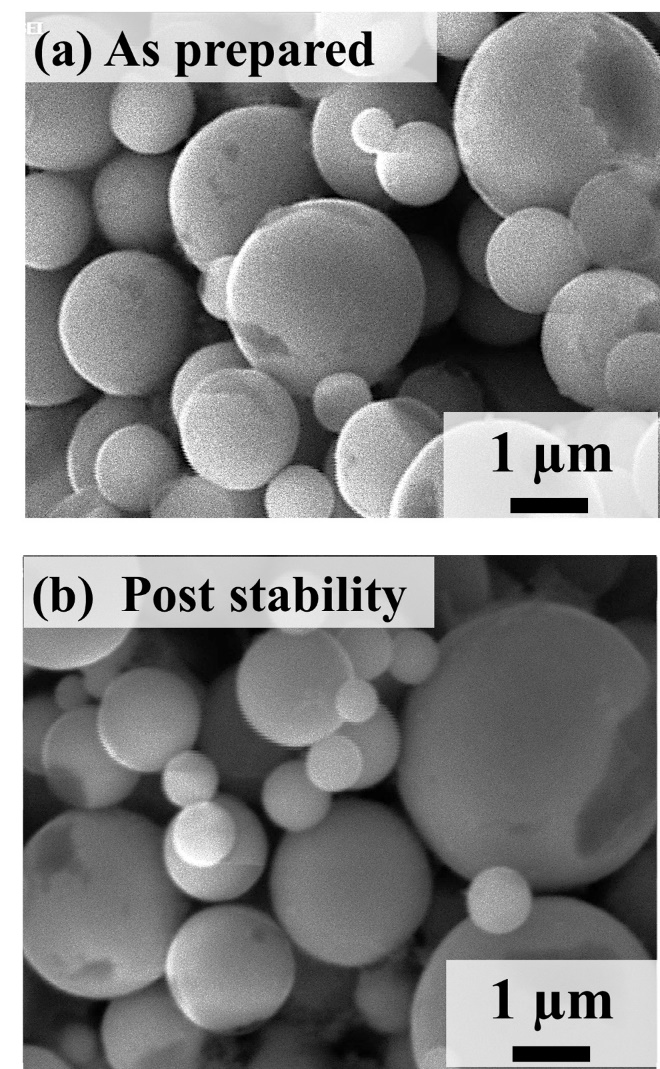


**Figure S57.** (a) – (b) SEM images comparison of Cr/NiBP MS electrocatalyst before/after stability test measurement at high current density of 1,000 mA/cm^2^ in 1 M KOH for 30-hr.

**S-3.5.2. Before/post stability test: Raman analysis**


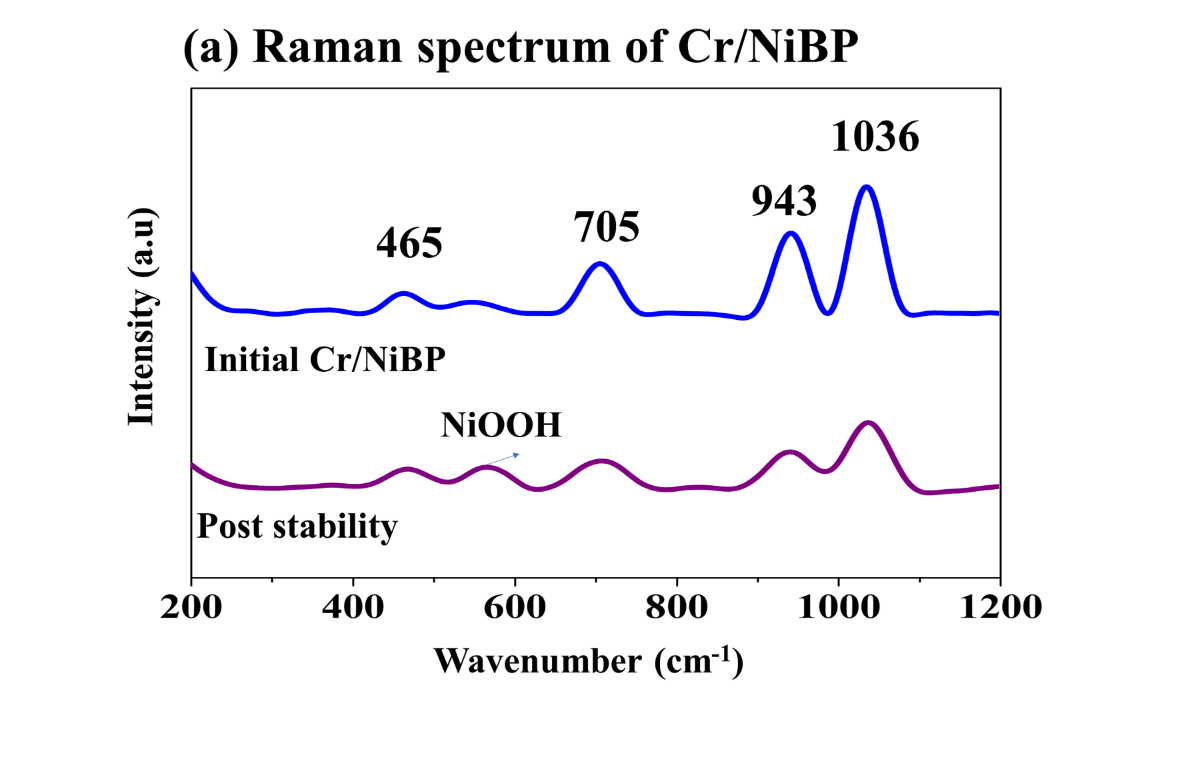


**Figure S58.** (a) Raman of Cr/NiBP MS electrocatalyst before/after stability test measurement at high current density of 1,000 mA/cm^2^ for 30-hr duration in 1 M KOH.

**S-3.5.3. Before/post stability test: XRD**


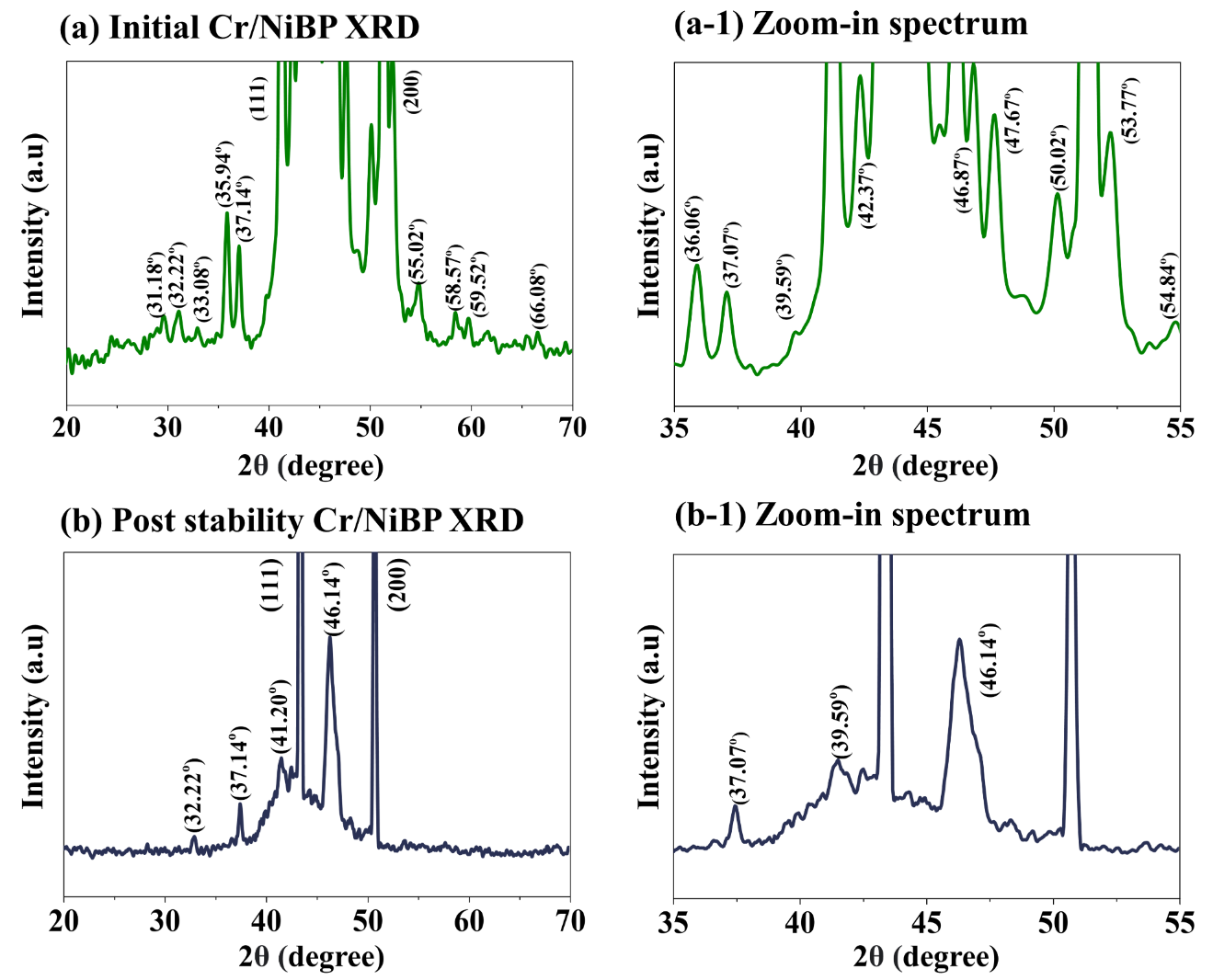


**Figure S59.** XRD analysis of Cr/NiBP MS electrocatalyst before/after stability test measurement at high current density of 1,000 mA/cm^2^ for 30-hr duration in 1 M KOH. (a) & (b) Full range XRD patterns of Cr/NiBP before/after stability test. (a-1) & (a-2) Zoom-in spectrum between 35 and 55^o^.

**S-3.5.4. Before/post stability test: full scan XPS**


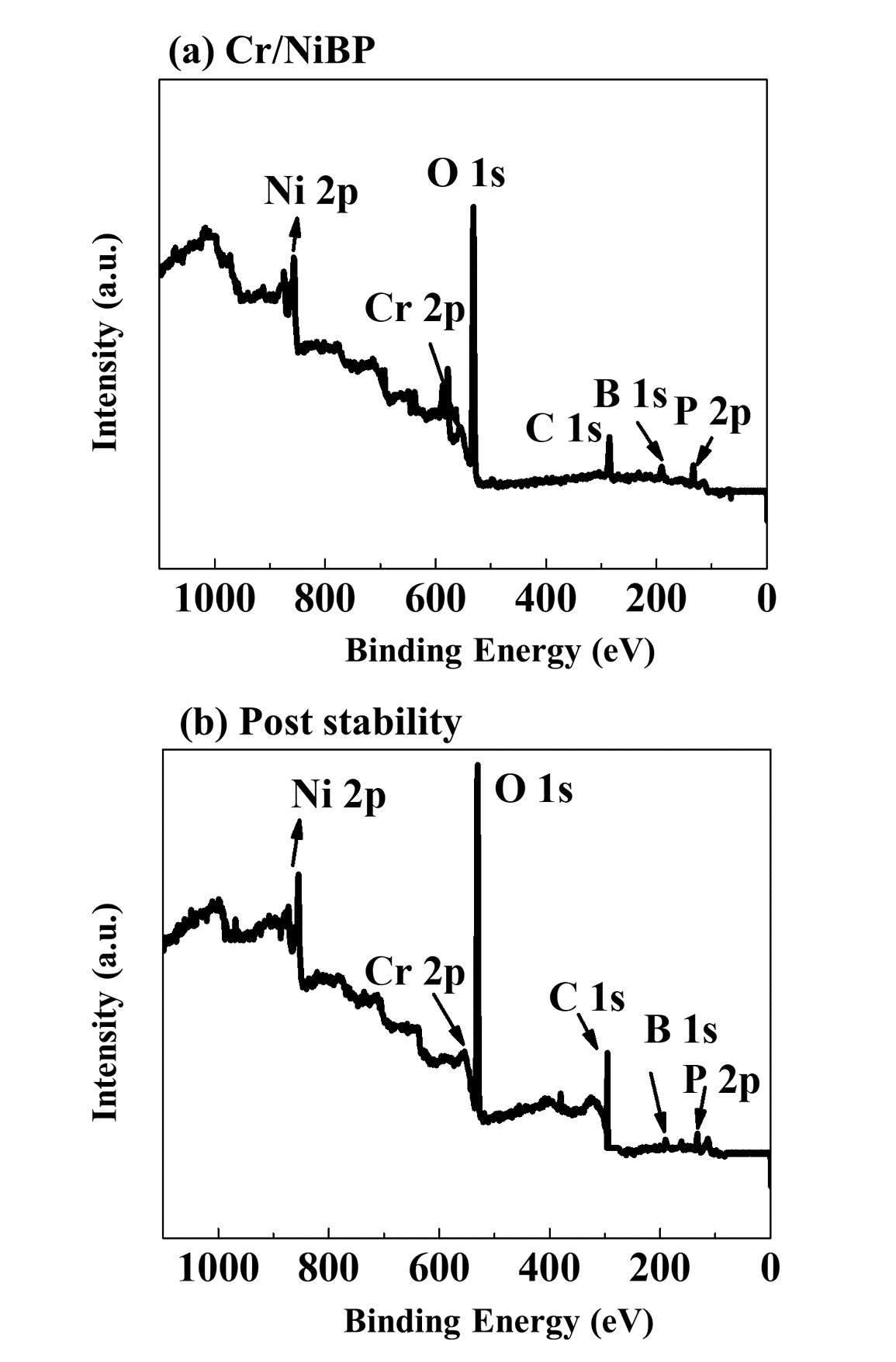


**Figure S60.** (a) & (b) Full-scan XPS spectra of Cr/NiBP MS electrocatalysts before/after stability test.

**S-3.5.5. After stability: high-resolution XPS**


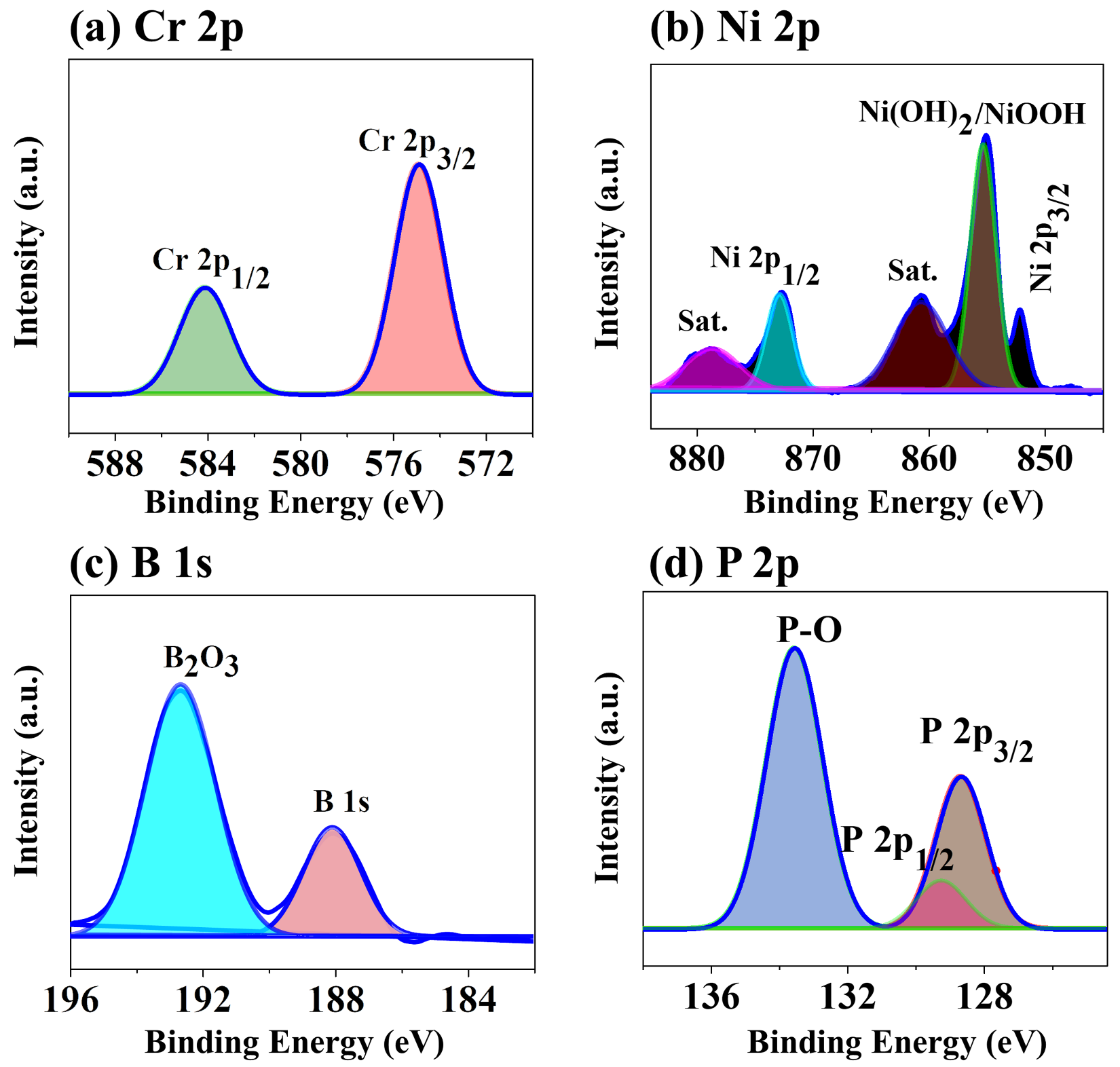


**Figure S61.** High-resolution XPS spectra of a) Cr 2p, (b) Ni 2p, (c) B 1s, (d) P 2p for Cr/NiBP MS electrocatalysts after the 30-hr stability measurement at 1,000 mA/cm^2^ in 1 M KOH.

**S-3.5.6. Before/post stability test: LSV**


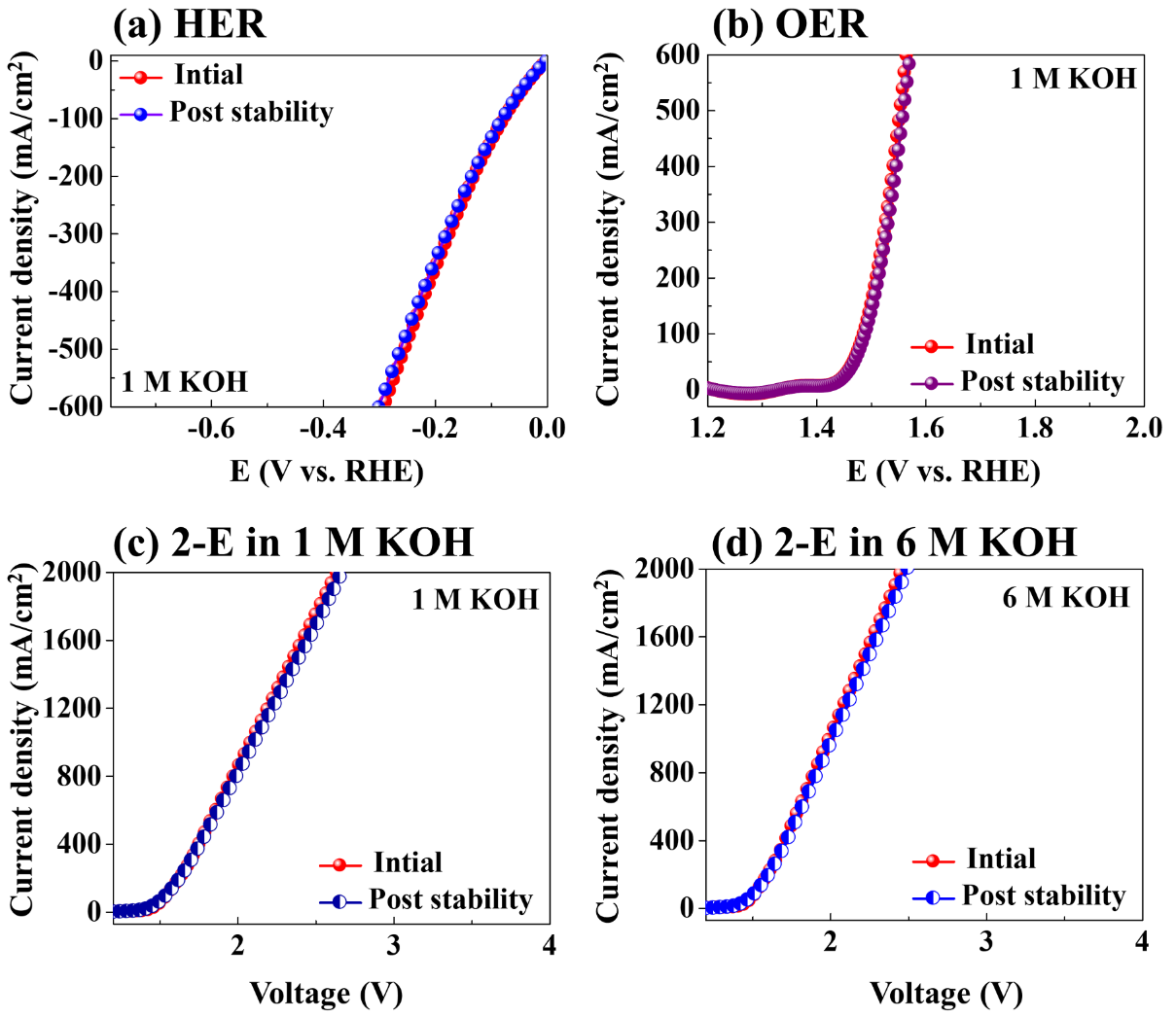


**Figure S62.** Electrochemical performance of Cr/NiBP MS electrocatalysts before/post stability test. (a) – (b) HER/OER LSV activity in 1 M KOH. (c) – (d) High current 2-E OWS activity 1 M and 6 M KOH solutions.

**S-4. Supplementary tables (Table S1- S10)**

**Table S1.** HER performance comparison with the state-of-art electrodes at 100, 300 and 600 mA/cm^2^  current density in 1 M KOH.

| Electrode | HER  Overpotential [mV]  100 at mA/cm^2^ | HER  Overpotential [mV]  300 at mA/cm^2^ | HER  Overpotential [mV]  600 at mA/cm^2^ | Year | Reference |
| --- | --- | --- | --- | --- | --- |
| Fe_2_P/CO_2_N | 74 | 120 | 165 | 2022 | ^[88]^ |
| Cr/NiBP | **76** | **176** | **291** | **This work** | **This** |
| Pt/NiO | 78 | 140 | NA | 2023 | ^[89]^ |
| NiCoP | 96 | 140 | 196 | 2023 | ^[90]^ |
| Cr/CoP | 100 | NA | NA | 2021 | ^[91]^ |
| W_3_CoB_3_ | 120 | NA | NA | 2022 | ^[6]^ |
| (FeNi)_2_P | 130 | 190 | 230 | 2023 | ^[92]^ |
| Ru/NiFe | 135 | NA | NA | 2024 | ^[93]^ |
| NiMoB | 140 | 280 | 370 | 2022 | ^[2]^ |
| CoMnB | 144 | 290 | NA | 2022 | ^[94]^ |
| RuFe-Ni_2_P | 151 | 202 | 245 | 2023 | ^[95]^ |
| NiCoSxSey | 180 | 210 | 280 | 2022 | ^[96]^ |
| Zr/NiP | 220 | 290 | NA | 2023 | ^[97]^ |
| NFMN/Ni_2_P | 230 | 310 | NA | 2024 | ^[98]^ |

**Table S2.** OER performance comparison with the state-of-art electrodes at 100, 300 and 600 mA/cm^2^ current density in 1 M KOH.

| Electrode | OER  Overpotential [mV]  100 at mA/cm^2^ | OER  Overpotential [mV]  300 at mA/cm^2^ | OER  Overpotential [mV]  600 at mA/cm^2^ | Year | Reference |
| --- | --- | --- | --- | --- | --- |
| NiCoP | 220 | 240 | 265 | 2023 | ^[90]^ |
| Fe/Ni_3_S_2_ | 240 | 260 | 330 | 2023 | ^[99]^ |
| Cr/NiBP | **250** | **300** | **330** | **This work** | **This** |
| NiFeV-S | 250 | 310 | NA | 2023 | ^[100]^ |
| (NiFeCo)_2_P | 260 | 320 | 450 | 2023 | ^[101]^ |
| Cr/CoP | 310 | 330 | NA | 2021 | ^[91]^ |
| W_3_CoB_3_ | 310 | NA | NA | 2022 | ^[6]^ |
| P/MoS_2_Ni_3_S_2_ | 312 | NA | NA | 2023 | ^[102]^ |
| Mo/NiSSe | 330 | 390 | 450 | 2023 | ^[103]^ |
| NiCoSxSeY | 330 | 360 | 400 | 2022 | ^[96]^ |
| Mo/CoFe | 332 | 420 | 525 | 2022 | ^[104]^ |
| RuCoMoOx | 340 | 370 | NA | 2023 | ^[105]^ |
| NiMoB | 350 | 540 | 600 | 2022 | ^[2]^ |
| CoMnB | 390 | 470 | NA | 2022 | ^[94]^ |

**Table S3.** HER/OER electrochemical properties of Cr/NiBP MS electrocatalyst in 1 M KOH. (Related to Figs. 3 & 4)

| Properties | HER | OER |
| --- | --- | --- |
| Tafel slopes | 32 mV/dec | 51 mV/dec |
| EIS values | 18.55 Ω | 17.98 Ω |
| Cdl values | 11.7 mF/cm^2^ | 16.27 mF/cm^2^ |
| ECSA | 78.12 cm^2^ | 102.50 cm^2^ |
| TOF values  (at 600 mA/cm^2^) | 10.51 site^-1^s^-1^ | 5.25 site^-1^s^-1^ |
| Faradic efficiency (FE) | 95.88 % | 95.69 % |

**Table S4.** Summarized HER/OER and two electrode (2-E) overall water splitting (OWS) bifunctional performance of Cr/NiBP electrode at 600 mA/cm^2^ in alkaline 1 M KOH (pH ~ 14), acidic 0.5 M H_2_SO_4_ (pH ~ 0) and neutral 1 M PBS (pH ~ 7.4) different pH solutions. (Related to Figs. 4 and 5)

| Electrolyte | HER  Overpotential | OER  Overpotential | 2-E OWS  Potential |
| --- | --- | --- | --- |
| Alkaline  1 M KOH | 291 mV | 330 mV | 1.86 V |
| Acidic  0.5 M H_2_SO_4_ | 323 mV | 500 mV | 1.97 V |
| Neutral  1 M PBS | 345 mV | 560 mV | 2.16 V |

**Table S5.** 2-E OWS performance comparison with the state-of-the-art bifunctional electrodes at 50, 100 and 200 mA/cm^2^ current density in 1 M PBS neutral electrolytes.

| Electrode | OWS  Potential [V]  50 at mA/cm^2^ | OWS  Potential [V]  100 at mA/cm^2^ | OWS  Potential [V]  200 at mA/cm^2^ | Year | Reference |
| --- | --- | --- | --- | --- | --- |
| NiBP | 1.54 | 1.76 | 1.96 | 2023 | ^[106]^ |
| V/FeBP | 1.56 | 1.61 | 1.81 | 2022 | ^[107]^ |
| Cr/NiBP | **1.59** | **1.65** | **1.76** | **This work** | **This** |
| W_3_CoB_3_ | 1.60 | 1.74 | 1.96 | 2022 | ^[6]^ |
| Mo/NiS_0.5_Se_0.5_ | 1.61 | 1.68 | 1.73 | 2023 | ^[103]^ |
| Al/FeCoNi_2_ | 1.66 | 1.71 | 1.79 | 2022 | ^[108]^ |
| CoMnB | 1.66 | 1.82 | 2.05 | 2022 | ^[17]^ |
| Ru_0.5_Ir_0.5_ | 1.67 | 1.79 | NA | 2022 | ^[109]^ |
| NiMoB | 1.75 | 1.88 | 2.09 | 2022 | ^[40]^ |
| CoP/CoOOH | 1.79 | 1.95 | NA | 2022 | ^[110]^ |
| CuMoO4 | 1.79 | 1.96 | NA | 2022 | ^[111]^ |
| Co_9_S_4_P_4_ | 1.81 | 1.91 | NA | 2019 | ^[112]^ |
| Co3O4 | 2.05 | 2.22 | NA | 2021 | ^[113]^ |
| NiFeMo | 2.05 | 2.28 | NA | 2021 | ^[114]^ |

**Table S6.** Overall water splitting (OWS) performance comparison with the state-of-the-art advanced bifunctional electrodes at 100, 300 and 600 mA/cm^2^ current density in 1 M KOH.

| Electrode | OWS  Potential [V]  100 at mA/cm^2^ | OWS  Potential [V]  300 at mA/cm^2^ | OWS  Potential [V]  600 at mA/cm^2^ | Year | Reference |
| --- | --- | --- | --- | --- | --- |
| FeCoNiCuMo | 1.53 | 1.59 | 1.66 | 2023 | ^[24]^ |
| Cr/NiBP | **1.54** | **1.69** | **1.86** | **This work** | **This** |
| Fe_2_P/CO_2_N | 1.56 | 1.63 | 1.69 | 2022 | ^[88]^ |
| V/FeBP | 1.56 | 1.74 | 1.95 | 2022 | ^[107]^ |
| Zn-Ni_2_P/Fe | 1.60 | 1.73 | NA | 2024 | ^[115]^ |
| MnO-CoP | 1.60 | 1.71 | 1.78 | 2023 | ^[116]^ |
| W_3_CoB_3_ | 1.62 | NA | NA | 2022 | ^[6]^ |
| CoMnB | 1.63 | 1.92 | 2.18 | 2022 | ^[94]^ |
| NiCoP | 1.65 | 1.78 | 1.83 | 2023 | ^[117]^ |
| (Fe,Ni)_2_P@Ni_2_P | 1.65 | 1.79 | 1.88 | 2023 | ^[118]^ |
| Ni_2_P/FeP | 1.70 | NA | NA | 2023 | ^[119]^ |
| Li-V/Ni_3_S_2_ | 1.70 | 1.80 | 1.93 | 2023 | ^[120]^ |
| Cr/CoP | 1.73 | NA | Na | 2021 | ^[91]^ |
| MoNiFe | 1.82 | 1.98 | NA | 2023 | ^[121]^ |

**Table S7.** High current OWS stability test comparison with the reported state-of-the-art electrocatalysts in alkaline media (1 M and 6 M KOH).

| Electrocatalysts | Stability  current  [mA/cm^2^] | Stability  duration [hours] | Solutions  [alkaline] | Temp.  (^o^C) | Reference |
| --- | --- | --- | --- | --- | --- |
| NiCoP | 1,000 mA/cm^2^ | 600 | 1 M KOH | **25 ^o^C** | ^[117]^ |
| Ni-Co-S-FeO_X_ | 1,000 mA/cm^2^ | 300 | 1 M KOH | **25 ^o^C** | ^[122]^ |
| Cr/NiBP | **1,000 mA/cm^2^** | **240** | **1 M KOH** | **25 ^o^C** | **This work** |
| Cr/NiBP | **1,000 mA/cm^2^** | **240** | **6 M KOH** | **60 ^o^C** | **This work** |
| Li-V/Ni_3_S_2_ | 1,000 mA/cm^2^ | 200 | 1 M KOH | 25 ^o^C | ^[120]^ |
| Ni-Mo-B | 1,000 mA/cm^2^ | 170 | 1 M KOH | 25 ^o^C | ^[123]^ |
| WO_2_-Ni/Ni(OH)_x_ | 1,000 mA/cm^2^ | 120 | 6 M KOH | 60 ^o^C | ^[124]^ |
| NiBP | 1,000 mA/cm^2^ | 120 | 6 M KOH | 60 ^o^C | ^[106]^ |
| (Fe,Ni)_2_P | 1,000 mA/cm^2^ | 120 | 1 M KOH | 25 ^o^C | ^[125]^ |
| MnOx/NiFeP | 1,000 mA/cm^2^ | 70 | 6 M KOH | 60 ^o^C | ^[126]^ |
| NiMoN | 1,000 mA/cm^2^ | 40 | 6 M KOH | 80 ^o^C | ^[127]^ |
| Ni_0.96_Co_0.04_P | 500 mA/cm^2^ | 500 | 1 M KOH | 25 ^o^C | ^[90]^ |
| IrNi-FeNi_3_ | 500 mA/cm^2^ | 100 | 6 M KOH | 60 ^o^C | ^[128]^ |
| NiFe(OH)x | 500 mA/cm^2^ | 100 | 1 M KOH | 25 ^o^C | ^[129]^ |
| N-CoP/CeO_2_ | 400 mA/cm^2^ | 504 | 1 M KOH | 25 ^o^C | ^[130]^ |
| Fe/Ni_3_S_2_ | 400 mA/cm^2^ | 120 | 6 KOH | 85 ^o^C | ^[99]^ |
| Ni_2_P-NiMoOx | 400 mA/cm^2^ | 60 | 6 M KOH | 60 ^o^C | ^[131]^ |

**Table S8.** 2-E OWS performances of Cr/NiBP electrode at 600 mA/cm^2^ in different configurations as compared to the benchmarks in alkaline 1 M KOH (pH ~ 14), acidic 0.5 M H_2_SO_4_ (pH ~ 0) and neutral 1 M PBS (pH ~ 7.4) solutions. Configuration of systems: Anode (+) ‖ Cathode (-). (Related to Figs. 5 and 6)

| System | Alkaline  1 M KOH | Acidic  0.5 M H_2_SO_4_ | Neutral  1 M PBS |
| --- | --- | --- | --- |
| Bifunctional  Cr/NiBP (+) ‖ Cr/NiBP (-) | 1.86 V | 1.97 V | 2.16 V |
| Hybrid  Cr/NiBP (+) ‖ Pt/C (-) | 1.78 V | 2.01 V | 2.14 V |
| Benchmark  RuO_2_ (+) ‖ Pt/C (-) | 2.15 V | 2.40 V | 2.78 V |

**Table S9.** 2-E high-current OWS performance summary of Cr/NiBP configurations as compared with the benchmark in 1 M and 6 M KOH solutions.

| System | 2-E OWS  1,000 mA/cm^2^ | 2-E OWS  2,000 mA/cm^2^ | Solution and temp. |
| --- | --- | --- | --- |
| Bifunctional | 2.08 V | 2.63 V | 1 M KOH  at 25 ^o^C |
| Hybrid | 1.95 V | 2.33 V | 1 M KOH  at 25 ^o^C |
| Benchmark | 2.30 V | 2.99 V | 1 M KOH  at 25 ^o^C |
| Bifunctional | 1.98 V | 2.46 V | 6 M KOH  at 60 ^o^C |
| Hybrid | 1.89 V | 2.25 V | 6 M KOH  at 60 ^o^C |

**Table S10.** 2-E OWS performance comparison with the state-of-the-art bifunctional electrodes at 50, 100 and 200 mA/cm^2^ current density in alkaline seawater (SW + 1 M KOH).

| Electrode | OWS  Potential [V]  50 at mA/cm^2^ | OWS  Potential [V]  100 at mA/cm^2^ | OWS  Potential [V]  200 at mA/cm^2^ | Year | Reference |
| --- | --- | --- | --- | --- | --- |
| Fe/N_2_P | 1.63 | 1.74 | 1.94 | 2024 | ^[132]^ |
| B/MnFe_2_O_4_ | 1.64 | 1.84 | NA | 2023 | ^[133]^ |
| Hybrid | **1.69** | **1.80** | **1.95** | **This work** | **This** |
| Ni@MoxC/Ni_2_P | 1.71 | 1.82 | 1.92 | 2023 | ^[134]^ |
| Bifunctional | **1.74** | **1.84** | **2.01** | **This work** | **This** |
| NiTe@FeOOH | 1.77 | 1.86 | 1.89 | 2023 | ^[135]^ |
| NiBP | 1.78 | 1.90 | 2.25 | 2023 | ^[1]^ |
| Ru/NiMoP_2_ | 1.80 | 2.02 | NA | 2023 | ^[136]^ |
| NiMoB | 1.83 | 1.95 | 2.38 | 2022 | ^[40]^ |
| Co-N_3_-P_1_ | 1.84 | 1.98 | 2.10 | 2022 | ^[137]^ |
| Er/MoO_2_ | 1.87 | 2.20 | NA | 2023 | ^[138]^ |
| CoMnB | 1.89 | 2.05 | 2.40 | 2022 | ^[17]^ |
| Mo/CoP | 1.96 | 2.16 | NA | 2022 | ^[139]^ |
| Ir-Co_2_P/Co_2_P_2_O_7_ | 2.35 | 3.10 | NA | 2023 | ^[56]^ |

**S-5. SI references :**

[1] M. Ahasan Habib, R. Mandavkar, S. Lin, S. Burse, T. Khalid, M. Hasan Joni, J. H. Jeong, J. Lee, *Chemical Engineering Journal* **2023**, *462*, 142177.

[2] R. Mandavkar, A. Habib, S. Lin, R. Kulkarni, S. Burse, J. Jeong, J. Lee, *Applied Materials Today* **2022**, *29*, 101579.

[3] Y. Sun, D. Jiang, J. Wang, A. Zhang, C. Wang, H. Zong, J. Xu, J. Liu, *Small* **2023**, *5*, 1.

[4] L. Cheng, M. Xu, Q. Zhang, G. Li, J. Chen, Y. Lou, *Journal of Alloys and Compounds* **2019**, *781*, 245.

[5] A. Shukla, S. C. Singh, C. S. Saraj, G. Verma, C. Guo, *Materials Today Chemistry* **2022**, *23*, 100691.

[6] M. A. Habib, R. Mandavkar, S. Burse, S. Lin, R. Kulkarni, C. S. Patil, J. H. Jeong, J. Lee, *Materials Today Energy* **2022**, *26*, 101021.

[7] B. Liu, S. Yan, Y. He, T. He, X. Yin, R. Song, Z. Zhang, H. Li, L. Chen, *Colloids and Surfaces A: Physicochemical and Engineering Aspects* **2022**, *653*, DOI 10.1016/j.colsurfa.2022.129972.

[8] T. Zhang, F. Song, Y. Qian, H. Gao, J. Shaw, Y. Rao, *ACS Applied Energy Materials* **2021**, *4*, 5434.

[9] L. Luo, S. Xu, X. Yu, Z. Wang, W. Li, Y. Du, M. Ruan, Q. Wu, *Dalton Transactions* **2022**, *51*, 10160.

[10] X. Wu, J. Chen, Z. Wen, *ACS Sustainable Chemistry and Engineering* **2021**, *9*, 13225.

[11] M. Yang, T. Feng, Y. Chen, J. Liu, X. Zhao, B. Yang, *Applied Catalysis B: Environmental* **2020**, *267*, 118657.

[12] Q. Wang, R. He, F. Yang, X. Tian, H. Sui, L. Feng, *Chemical Engineering Journal* **2023**, *456*, 141056.

[13] A. M. Ibrahim, M. S. Abdel-wahab, M. A. K. Elfayoumi, W. Z. Tawfik, *International Journal of Hydrogen Energy* **2023**, *48*, 1863.

[14] Q. Zhou, G. Song, J. Zou, S. Luo, A. Meng, Z. Li, *International Journal of Hydrogen Energy* **2023**, *48*, 15921.

[15] W. Li, Y. Jiang, Y. Li, Q. Gao, W. Shen, Y. Jiang, R. He, M. Li, *Chemical Engineering Journal* **2021**, *425*, 130651.

[16] X. Mu, K. Wang, K. Lv, B. Feng, X. Yu, L. Li, X. Zhang, X. Yang, Z. Lu, *ACS Applied Materials and Interfaces* **2023**, *15*, 16552.

[17] S. Lin, M. A. Habib, R. Mandavkar, R. Kulkarni, S. Burse, Y. U. Chung, C. Liu, Z. Wang, S. Lin, J. H. Jeong, J. Lee, *Advanced Sustainable Systems* **2022**, *6*, 1.

[18] X. Guo, Y. Hou, R. Ren, J. Chen, *Nanoscale Research Letters* **2017**, *12*, DOI 10.1186/s11671-017-2248-9.

[19] S. Anantharaj, S. Noda, M. Driess, P. W. Menezes, *ACS Energy Letters* **2021**, *6*, 1607.

[20] N. Elgrishi, K. J. Rountree, B. D. McCarthy, E. S. Rountree, T. T. Eisenhart, J. L. Dempsey, *Journal of Chemical Education* **2018**, *95*, 197.

[21] B. Jing, S. You, Y. Ma, Z. Xing, H. Chen, Y. Dai, C. Zhang, N. Ren, J. Zou, *Applied Catalysis B: Environmental* **2019**, *244*, 465.

[22] Q. Zhou, G. Song, J. Zou, S. Luo, A. Meng, Z. Li, *International Journal of Hydrogen Energy* **2023**, *48*, 15921.

[23] M. P. Minadakis, R. Canton-Vitoria, C. Stangel, E. Klontzas, R. Arenal, J. Hernández-Ferrer, A. M. Benito, W. K. Maser, N. Tagmatarchis, *ChemSusChem* **2023**, *16*, DOI 10.1002/cssc.202202322.

[24] C. L. Huang, Y. G. Lin, C. L. Chiang, C. K. Peng, D. Senthil Raja, C. T. Hsieh, Y. A. Chen, S. Q. Chang, Y. X. Yeh, S. Y. Lu, *Applied Catalysis B: Environmental* **2023**, *320*, 122016.

[25] R. Martínez-Hincapié, J. Wegner, M. U. Anwar, A. Raza-Khan, S. Franzka, S. Kleszczynski, V. Čolić, *Electrochimica Acta* **2024**, *476*, 143663.

[26] A. Karmakar, S. Kundu, *Materials Today Energy* **2023**, *33*, 101259.

[27] S. Anantharaj, S. Kundu, *ACS Energy Letters* **2019**, *4*, 1260.

[28] Y. Huang, L. W. Jiang, B. Y. Shi, K. M. Ryan, J. J. Wang, *Advanced Science* **2021**, *8*, 1.

[29] Y. Han, P. Li, Z. Tian, C. Zhang, Y. Ye, X. Zhu, C. Liang, *ACS Applied Energy Materials* **2019**, *2*, 6302.

[30] S. Lin, R. Mandavkar, M. A. Habib, S. A. Dristy, M. H. Joni, J.-H. Jeong, J. Lee, *Journal of Colloid and Interface Science* **2024**.

[31] S. Sultan, M. Ha, D. Y. Kim, J. N. Tiwari, C. W. Myung, A. Meena, T. J. Shin, K. H. Chae, K. S. Kim, *Nature Communications* **2019**, *10*, 1.

[32] A. Habib, S. Burse, S. Lin, R. Mandavkar, M. H. Joni, J. Jeong, S. Lee, J. Lee, **2023**, *2307533*, 1.

[33] Y. J. Shih, Y. H. Huang, C. P. Huang, *Electrochimica Acta* **2018**, *263*, 261.

[34] S. Li, G. Zhang, X. Tu, J. Li, *ChemElectroChem* **2018**, *5*, 701.

[35] S. S. Jeon, P. W. Kang, M. Klingenhof, H. Lee, F. Dionigi, P. Strasser, *ACS Catalysis* **2023**, *13*, 1186.

[36] J. Kim, H. Kim, S. K. Kim, S. H. Ahn, *Journal of Materials Chemistry A* **2018**, *6*, 6282.

[37] Z. Qiu, Y. Ma, T. Edvinsson, *Nano Energy* **2019**, *66*, 104118.

[38] Y. Yang, X. Su, L. Zhang, P. Kerns, L. Achola, V. Hayes, R. Quardokus, S. L. Suib, J. He, *ChemCatChem* **2019**, *11*, 1689.

[39] M. Fazil, T. Ahmad, *Catalysts* **2023**, *13*, DOI 10.3390/catal13010093.

[40] R. Mandavkar, M. A. Habib, S. Lin, R. Kulkarni, S. Burse, J. H. Jeong, J. Lee, *Applied Materials Today* **2022**, *29*, 101579.

[41] J. Jiang, Y. Wu, H. Chen, Z. Wan, D. Ding, L. Xia, X. Guo, P. Yu, *Journal of Colloid and Interface Science* **2023**, *633*, 303.

[42] M. A. Sayeed, H. K. Rouf, K. Md Amjad Hussain, *Materials Research Express* **2021**, *8*, DOI 10.1088/2053-1591/ac1964.

[43] J. Li, H. Chen, Y. Liu, R. Gao, X. Zou, *Journal of Materials Chemistry A* **2019**, *7*, 5288.

[44] F. Razmjooei, C. Pak, J. S. Yu, *ChemElectroChem* **2018**, *5*, 1985.

[45] N. Mironova-Ulmane, A. Kuzmin, I. Sildos, M. Pärs, *Central European Journal of Physics* **2011**, *9*, 1096.

[46] B. Jansi Rani, N. Dhivya, G. Ravi, S. S. Zance, R. Yuvakkumar, S. I. Hong, *ACS Omega* **2019**, *4*, 10302.

[47] A. M. Bamuqaddam, S. A. Aladeemy, M. A. Ghanem, A. M. Al-Mayouf, N. H. Alotaibi, F. Marken, *Nanomaterials* **2022**, *12*, DOI 10.3390/nano12050879.

[48] J. Prywer, D. Kasprowicz, T. Runka, *Spectrochimica Acta - Part A: Molecular and Biomolecular Spectroscopy* **2016**, *158*, 18.

[49] G. Solomon, A. Landström, R. Mazzaro, M. Jugovac, P. Moras, E. Cattaruzza, V. Morandi, I. Concina, A. Vomiero, *Advanced Energy Materials* **2021**, *11*, DOI 10.1002/aenm.202101324.

[50] J. Hao, J. Li, Y. Zhu, S. Sun, S. Lu, M. Du, H. Zhu, *Chemical Communications* **2022**, *59*, 772.

[51] K. Bhunia, M. Chandra, S. Kumar Sharma, D. Pradhan, S. J. Kim, *Coordination Chemistry Reviews* **2023**, *478*, 214956.

[52] L. Meng, H. Xuan, J. Wang, X. Liang, Y. Li, J. Yang, P. Han, *International Journal of Hydrogen Energy* **2023**, DOI 10.1016/j.ijhydene.2023.08.143.

[53] B. Li, Z. Tian, L. Li, Y. H. Wang, Y. Si, H. Wan, J. Shi, G. F. Huang, W. Hu, A. Pan, W. Q. Huang, *ACS Nano* **2023**, *17*, 3465.

[54] Y. Dai, G. Xie, X. Jia, B. Guo, J. R. Gong, *Applied Surface Science* **2023**, *610*, 155501.

[55] Q. Wang, C. Q. Xu, W. Liu, S. F. Hung, H. Bin Yang, J. Gao, W. Cai, H. M. Chen, J. Li, B. Liu, *Nature Communications* **2020**, *11*, 1.

[56] V. H. Hoa, M. Austeria, H. Thi Dao, M. Mai, D. H. Kim, *Applied Catalysis B: Environmental* **2023**, *327*, 122467.

[57] X. Zheng, S. Sun, Y. Liu, D. Li, D. Tian, J. Zhu, D. Jiang, *Applied Surface Science* **2023**, *609*, DOI 10.1016/j.apsusc.2022.155406.

[58] V. H. Hoa, D. T. Tran, S. Prabhakaran, D. H. Kim, N. Hameed, H. Wang, N. H. Kim, J. H. Lee, *Nano Energy* **2021**, *88*, 106277.

[59] P. Cui, P. Fu, D. Wei, M. Li, D. Song, X. Yue, Y. Li, Z. Zhang, Y. Li, J. M. Mbengue, *RSC Advances* **2015**, *5*, 75622.

[60] Y. Luo, B. Wang, S. Smeets, J. Sun, W. Yang, X. Zou, *Nature Chemistry* **2023**, *15*, DOI 10.1038/s41557-022-01131-8.

[61] D. Zhang, J. Z. Soo, H. H. Tan, C. Jagadish, K. Catchpole, S. K. Karuturi, *Advanced Energy and Sustainability Research* **2021**, *2*, 2000071.

[62] X. Chen, R. Jiang, C. Dong, H. Liu, J. Yang, X. Du, *ChemElectroChem* **2022**, *9*, DOI 10.1002/celc.202101566.

[63] D. Briggs, *Handbook of Adhesion: Second Edition* **2005**, 621.

[64] Y. Li, Z. Zhu, Y. L. Zhong, Y. Jin, P. Saha, Q. Cheng, *Journal of Power Sources* **2024**, *614*, 234969.

[65] C. H. Chuang, P. H. Kang, Y. Y. Lai, C. H. Hou, W. C. Tseng, Y. J. Huang, M. H. Fang, J. J. Shyue, C. C. Kaun, Y. J. Cheng, *ChemSusChem* **2023**, *16*, DOI 10.1002/cssc.202300820.

[66] Y. Wu, X. Tao, Y. Qing, H. Xu, F. Yang, S. Luo, C. Tian, M. Liu, X. Lu, *Advanced Materials* **2019**, *31*, 1.

[67] N. Yao, P. Li, Z. Zhou, Y. Zhao, G. Cheng, S. Chen, W. Luo, *Advanced Energy Materials* **2019**, *9*, 1.

[68] Z. Jiang, S. Song, X. Zheng, X. Liang, Z. Li, H. Gu, Z. Li, Y. Wang, S. Liu, W. Chen, D. Wang, Y. Li, *Journal of the American Chemical Society* **2022**, *144*, 19619.

[69] N. Weidler, J. Schuch, F. Knaus, P. Stenner, S. Hoch, A. Maljusch, R. Schäfer, B. Kaiser, W. Jaegermann, *Journal of Physical Chemistry C* **2017**, *121*, 6455.

[70] N. N. Rao, C. Alex, M. Mukherjee, S. Roy, A. Tayal, A. Datta, N. S. John, *ACS Catalysis* **2024**, *14*, 981.

[71] S. Yang, J. Y. Zhu, X. N. Chen, M. J. Huang, S. H. Cai, J. Y. Han, J. Sen Li, *Applied Catalysis B: Environmental* **2022**, *304*, 120914.

[72] D. Zhang, J. Shi, Y. Qi, X. Wang, H. Wang, M. Li, S. Liu, C. Li, *Advanced Science* **2018**, *5*, 1.

[73] A. Kumar, J. Muhommad, S. K. Purkayastha, A. K. Guha, M. R. Das, S. Deka, *ACS Sustainable Chemistry and Engineering* **2023**, *11*, 2541.

[74] X. Feng, Y. Shi, J. Liang, W. Li, X. Gou, *Journal of Industrial and Engineering Chemistry* **2023**, *118*, 383.

[75] V. T. Nguyen, V. C. Nguyen, T. C. Phan, H. S. Choi, H. C. Kim, *International Journal of Hydrogen Energy* **2022**, *47*, 31614.

[76] J. Yan, L. Kong, Y. Ji, J. White, Y. Li, J. Zhang, P. An, S. Liu, S. T. Lee, T. Ma, *Nature Communications* **2019**, *10*, 1.

[77] N. Hales, T. J. Schmidt, E. Fabbri, *Current Opinion in Electrochemistry* **2023**, *38*, 101231.

[78] Y. Zeng, M. Zhao, Z. Huang, W. Zhu, J. Zheng, Q. Jiang, Z. Wang, H. Liang, *Advanced Energy Materials* **2022**, *12*, 1.

[79] Q. Zhang, C. Zhang, J. Liang, P. Yin, Y. Tian, *ACS Sustainable Chemistry and Engineering* **2017**, *5*, 3808.

[80] C. C. Lin, C. C. L. McCrory, *ACS Catalysis* **2017**, *7*, 443.

[81] Y. Fan, R. Li, C. Zhao, A. Hu, B. Zhou, Y. Pan, J. Chen, Z. Yan, M. Liu, M. He, *Journal of Colloid and Interface Science* **2023**, *645*, 439.

[82] D. Xu, M. B. Stevens, Y. Rui, G. DeLuca, S. W. Boettcher, E. Reichmanis, Y. Li, Q. Zhang, H. Wang, *Electrochimica Acta* **2018**, *265*, 10.

[83] A. Karmakar, S. Kundu, *Materials Today Energy* **2023**, *33*, 101259.

[84] T. Kim, S. B. Roy, S. Moon, S. H. Yoo, H. Choi, V. G. Parale, Y. Kim, J. Lee, S. C. Jun, K. Kang, S. H. Chun, K. Kanamori, H. H. Park, *ACS Nano* **2022**, *16*, 1625.

[85] S. Sultan, M. Ha, D. Y. Kim, J. N. Tiwari, C. W. Myung, A. Meena, T. J. Shin, K. H. Chae, K. S. Kim, *Nature communications* **2019**, *10*, 1.

[86] M. Juthathan, T. Chantarojsiri, K. Chainok, T. Butburee, P. Thamyongkit, T. Tuntulani, P. Leeladee, *Dalton Transactions* **2023**, *52*, 11407.

[87] D. Liu, R. Tong, Y. Qu, Q. Zhu, X. Zhong, M. Fang, K. Ho Lo, F. Zhang, Y. Ye, Y. Tang, S. Chen, G. Xing, H. Pan, *Applied Catalysis B: Environmental* **2020**, *267*, DOI 10.1016/j.apcatb.2020.118721.

[88] X. Zhou, Y. Mo, F. Yu, L. Liao, X. Yong, F. Zhang, D. Li, Q. Zhou, T. Sheng, H. Zhou, *Advanced Functional Materials* **2022**, DOI 10.1002/adfm.202209465.

[89] K. Wang, S. Wang, K. S. Hui, J. Li, C. Zha, D. A. Dinh, Z. Shao, B. Yan, Z. Tang, K. N. Hui, *Advanced Functional Materials* **2023**, *33*, 1.

[90] X. Lv, S. Wan, T. Mou, X. Han, Y. Zhang, Z. Wang, X. Tao, *Advanced Functional Materials* **2023**, *33*, DOI 10.1002/adfm.202205161.

[91] W. Li, Y. Jiang, Y. Li, Q. Gao, W. Shen, Y. Jiang, R. He, M. Li, *Chemical Engineering Journal* **2021**, *425*, 130651.

[92] Y. Li, X. Yu, J. Gao, Y. Ma, *Chemical Engineering Journal* **2023**, *470*, DOI 10.1016/j.cej.2023.144373.

[93] Y. Chen, Y. Liu, W. Zhai, H. Liu, T. Sakthivel, S. Guo, Z. Dai, *Advanced Energy Materials* **2024**, *2400059*, 1.

[94] S. Lin, A. Habib, R. Mandavkar, R. Kulkarni, S. Burse, Y. Chung, C. Liu, Z. Wang, S. Lin, J. Jeong, J. Lee, **2022**, *2200213*, 1.

[95] X. Zhai, Q. Yu, J. Chi, X. Wang, B. Li, B. Yang, Z. Li, J. Lai, L. Wang, *Nano Energy* **2023**, *105*, 108008.

[96] S. Ma, J. Huang, C. Zhang, G. Chen, W. Chen, T. Shao, T. Li, X. Zhang, T. Gong, K. K. Ostrikov, *Chemical Engineering Journal* **2022**, *435*, 134859.

[97] H. Fan, J. Jia, D. Wang, J. Fan, J. Wu, J. Zhao, X. Cui, *Chemical Engineering Journal* **2023**, *455*, 140908.

[98] H. Du, T. Wang, S. He, B. Li, K. Wang, Q. Chen, Z. Du, W. Ai, W. Huang, *Advanced Functional Materials* **2024**, *34*, 1.

[99] B. Zhong, B. Cheng, Y. Zhu, R. Ding, P. Kuang, J. Yu, *Journal of Colloid and Interface Science* **2023**, *629*, 846.

[100] W. Wu, L. Peng, B. Min, J. Huang, S. Liu, K. Lu, S. Lu, D. Jing, M. Zheng, M. Liu, *Applications in Energy and Combustion Science* **2023**, *14*, 100148.

[101] B. Wang, G. Zhao, Q. Yan, N. Wang, B. Duolihong, H. Xie, X. Xia, *Chemical Engineering Journal* **2023**, *462*, 142138.

[102] F. Jia, X. Zou, X. Wei, W. Bao, T. Ai, W. Li, Y. Guo, *Materials* **2023**, *16*, DOI 10.3390/ma16093411.

[103] Y. Wang, X. Li, Z. Huang, H. Wang, Z. Chen, J. Zhang, X. Zheng, Y. Deng, W. Hu, *Angewandte Chemie - International Edition* **2023**, *62*, DOI 10.1002/anie.202215256.

[104] G. Zhao, B. Wang, Q. Yan, X. Xia, *Journal of Alloys and Compounds* **2022**, *902*, 163738.

[105] J. Ren, Y. Du, Y. Wang, S. Zhao, B. Yang, B. Li, L. Wang, *Chemical Engineering Journal* **2023**, *469*, 143993.

[106] M. Ahasan Habib, R. Mandavkar, S. Lin, S. Burse, T. Khalid, M. Hasan Joni, J. H. Jeong, J. Lee, *Chemical Engineering Journal* **2023**, *462*, 142177.

[107] S. Burse, R. Kulkarni, R. Mandavkar, M. A. Habib, S. Lin, Y.-U. Chung, J.-H. Jeong, J. Lee, *Nanomaterials* **2022**, *12*, 3283.

[108] F. Te Tsai, Y. Y. Chuang, H. H. Hsieh, Y. H. Chen, C. W. Pao, J. L. Chen, C. Y. Lu, C. K. Hao, W. F. Liaw, *ACS Applied Energy Materials* **2022**, DOI 10.1021/acsaem.2c00238.

[109] Y. Jiang, Y. Mao, Y. Jiang, H. Liu, W. Shen, M. Li, R. He, *Chemical Engineering Journal* **2022**, *450*, 137909.

[110] B. Zhang, J. Shan, W. Wang, P. Tsiakaras, Y. Li, *Small* **2022**, *18*, DOI 10.1002/smll.202106012.

[111] F. F. Dai, Y. X. Xue, D. L. Gao, Y. X. Liu, J. H. Chen, Q. J. Lin, W. W. Lin, Q. Yang, *Dalton Transactions* **2022**, *51*, 12736.

[112] Y. Tan, M. Luo, P. Liu, C. Cheng, J. Han, K. Watanabe, M. Chen, *ACS Applied Materials and Interfaces* **2019**, *11*, 3880.

[113] L. Zhang, B. Liu, N. Zhang, M. Ma, *Nano Research* **2018**, *11*, 323.

[114] X. Cao, R. Fan, J. Zhou, C. Chen, S. Xu, S. Zou, W. Dong, X. Su, S. Ju, M. Shen, *Chemical Communications* **2022**, *58*, 1569.

[115] J. Nie, J. Shi, T. Huang, M. Y. Xie, Z. Y. Ouyang, M. H. Xian, G. F. Huang, H. Wan, W. Hu, W. Q. Huang, *Advanced Functional Materials* **2024**, *2314172*, 1.

[116] Y. Dong, Z. Deng, H. Zhang, G. Liu, X. Wang, *Nano Letters* **2023**, *23*, 9087.

[117] D. Chen, H. Bai, J. Zhu, C. Wu, H. Zhao, D. Wu, J. Jiao, P. Ji, S. Mu, *Advanced Energy Materials* **2023**, *13*, 1.

[118] Y. Li, X. Yu, J. Gao, Y. Ma, *Chemical Engineering Journal* **2023**, *470*, 144373.

[119] M. Jiang, H. Zhai, L. Chen, L. Mei, P. Tan, K. Yang, J. Pan, *Advanced Functional Materials* **2023**, *33*, DOI 10.1002/adfm.202302621.

[120] Q. N. Ha, N. Susanto Gultom, C. H. Yeh, D. H. Kuo, *Chemical Engineering Journal* **2023**, *472*, 144931.

[121] N. S. Gultom, T. S. Chen, M. Z. Silitonga, D. H. Kuo, *Applied Catalysis B: Environmental* **2023**, *322*, 122103.

[122] S. Li, Y. Liu, K. Feng, C. Li, J. Xu, C. Lu, H. Lin, Y. Feng, D. Ma, J. Zhong, *Angewandte Chemie* **2023**, *135*, DOI 10.1002/ange.202308670.

[123] H. Liu, X. Li, L. Chen, X. Zhu, P. Dong, M. O. L. Chee, M. Ye, Y. Guo, J. Shen, *Advanced Functional Materials* **2022**, *32*, DOI 10.1002/adfm.202107308.

[124] J. Liu, G. Qian, T. Yu, J. Chen, C. Zhu, Y. Li, J. He, L. Luo, S. Yin, *Chemical Engineering Journal* **2022**, *431*, DOI 10.1016/j.cej.2021.134247.

[125] Y. Li, X. Yu, J. Gao, Y. Ma, *Chemical Engineering Journal* **2023**, *470*, 144373.

[126] P. Wang, Y. Luo, G. Zhang, M. Wu, Z. Chen, S. Sun, Z. Shi, *Small* **2022**, *18*, 1.

[127] Y. Liu, B. Zhou, Y. Zhang, W. Xiao, B. Li, Z. Wu, L. Wang, *Journal of Colloid and Interface Science* **2023**, *637*, 104.

[128] Y. Wang, G. Qian, Q. Xu, H. Zhang, F. Shen, L. Luo, S. Yin, *Applied Catalysis B: Environmental* **2021**, *286*, 119881.

[129] H. Zhang, Y. Zhou, M. Xu, A. Chen, Z. Ni, O. Akdim, T. Wågberg, X. Huang, G. Hu, *ACS Nano* **2023**, *17*, 636.

[130] L. Zhang, Y. Lei, W. Xu, D. Wang, Y. Zhao, W. Chen, X. Xiang, X. Pang, B. Zhang, H. Shang, *Chemical Engineering Journal* **2023**, *460*, 141119.

[131] J. T. Ren, L. Chen, H. Y. Wang, W. W. Tian, X. L. Song, Q. H. Kong, Z. Y. Yuan, *ACS Catalysis* **2023**, *13*, 9792.

[132] A. Muthurasu, T. H. Ko, T. W. Kim, K. Chhetri, H. Y. Kim, *Advanced Functional Materials* **2024**, *2404254*, 1.

[133] M. Chen, N. Kitiphatpiboon, C. Feng, Q. Zhao, A. Abudula, Y. Ma, K. Yan, G. Guan, *Applied Catalysis B: Environmental* **2023**, *330*, 122577.

[134] J. Wang, D. T. Tran, K. Chang, S. Prabhakaran, J. Zhao, D. H. Kim, N. H. Kim, J. H. Lee, *Nano Energy* **2023**, *111*, 108440.

[135] X. Gao, J. Chen, Y. Yu, F. Wang, X. Wu, X. Wang, W. Mao, J. Li, W. Huang, Q. Chen, R. Li, C. You, S. Wang, X. Tian, Z. Kang, *Chemical Engineering Journal* **2023**, *474*, 145568.

[136] H. K. Sadhanala, A. Gupta, A. Gedanken, *Sustainable Energy and Fuels* **2023**, 4677.

[137] X. Wang, X. Zhou, C. Li, H. Yao, C. Zhang, J. Zhou, R. Xu, L. Chu, H. Wang, M. Gu, H. Jiang, M. Huang, *Advanced Materials* **2022**, *34*, 1.

[138] T. Yang, H. Lv, Q. Quan, X. Li, H. Lu, X. Cui, G. Liu, L. Jiang, *Applied Surface Science* **2023**, *615*, 156360.

[139] Y. Yu, J. Li, J. Luo, Z. Kang, C. Jia, Z. Liu, W. Huang, Q. Chen, P. Deng, Y. Shen, X. Tian, *Materials Today Nano* **2022**, *18*, DOI 10.1016/j.mtnano.2022.100216.
